# Human Milk and Infant Gut Microbiome in Association With Infant Fecal Metabolome and Child Blood Pressure

**DOI:** 10.1001/jamanetworkopen.2025.59385

**Published:** 2026-02-13

**Authors:** Tiange Liu, Charisse Petersen, Ni Zhao, Theo J. Moraes, Padmaja Subbarao, Elinor Simons, Meghan B. Azad, Kozeta Miliku, Lars Bode, Brianna Moore, Stuart Turvey, Noel T. Mueller

**Affiliations:** 1 Department of Epidemiology, Johns Hopkins Bloomberg School of Public Health, Baltimore, Maryland; 2 Division of Women’s Health, Department of Medicine, Brigham and Women’s Hospital and Harvard Medical School, Boston, Massachusetts; 3 Department of Pediatrics, British Columbia Children’s Hospital, University of British Columbia, Vancouver, British Columbia, Canada; 4 Department of Biostatistics, Johns Hopkins Bloomberg School of Public Health, Baltimore, Maryland; 5 Department of Pediatrics, Hospital for Sick Children, University of Toronto, Toronto, Ontario, Canada; 6 Department of Translational Medicine, Hospital for Sick Children, University of Toronto, Toronto, Ontario, Canada; 7 Section of Allergy and Immunology, University of Manitoba, Winnipeg, Manitoba, Canada; 8 Department of Pediatrics and Child Health, University of Manitoba, Winnipeg, Manitoba, Canada; 9 Manitoba Interdisciplinary Lactation Centre, Children’s Hospital Research Institute of Manitoba, Winnipeg, Manitoba, Canada; 10 Department of Pediatrics and Child Health, University of Manitoba, Winnipeg, Manitoba, Canada; 11 Department of Nutritional Sciences, University of Toronto, Toronto, Ontario, Canada; 12 Department of Pediatrics, Larsson-Rosenquist Foundation Mother-Milk-Infant Center of Research Excellence, University of California, San Diego, La Jolla; 13 Department of Pediatrics, University of Colorado Anschutz Medical Campus, Aurora; 14 Lifecourse Epidemiology of Adiposity and Diabetes Center, University of Colorado Anschutz Medical Campus, Aurora; 15 Human Milk Institute, University of California, San Diego, La Jolla

## Abstract

**Importance:**

Infant milk feeding type (eg, human milk vs formula) and infant gut microbes have each been associated with differences in microbial metabolites and childhood blood pressure; however, evidence remains limited regarding how specific infant gut microbes, at a species or strain level, in combination with milk feeding type, shape microbial metabolites and blood pressure.

**Objective:**

To investigate whether human milk feeding and infant gut microbes, including *Bifidobacterium longum *subsp* infantis* (*B infantis*) and other milk-degrading microbes, are associated with infant fecal metabolites and childhood systolic blood pressure (SBP).

**Design, Setting, and Participants:**

This cohort study was part of the Canadian Healthy Infant Longitudinal Development (CHILD) cohort study, a prospective multicenter, contemporary, population-based cohort of pregnant mothers and their offspring recruited between 2009 and 2012. Data were collected from 2009 to 2018 and analyzed from January to December 2024. Participants included a subset of children born at 35 weeks of gestation or later without congenital abnormalities or respiratory distress syndrome and with available data on gut microbiome, fecal metabolome, SBP, and covariates.

**Exposures:**

Gut microbiome, fecal metabolome, and human milk feeding status at ages 3 months and 1 year.

**Main Outcomes and Measures:**

Age-, sex-, and height-specific SBP percentile, measured at ages 3 and 5 years.

**Results:**

A total of children (610 [46.1%] girls; 982 children [74.2%] delivered vaginally; mean [SD] maternal age at delivery, 33.3 [4.5] years) were included. At age 3 months, but not at age 1 year, human milk feeding and presence of *B infantis* showed interactive associations with infant fecal metabolites at ages 3 months and 1 year and SBP at ages 3 and 5 years. Among infants harboring *B infantis* at age 3 months, mixed feeding (difference, −14.81 [95% CI, −27.05 to −2.56] percentile) and exclusive human milk feeding (difference, −17.16 [95% CI, −29.48 to −4.83] percentile) were associated with a lower childhood SBP, whereas no association was observed among infants without *B infantis*. Several additional infant gut microbes (eg, *Eggerthella lenta*, *Veillonella dispar*) and fecal metabolites (eg, creatinine, succinic acid) also demonstrated feeding- or *B infantis*–dependent associations with childhood SBP.

**Conclusions and Relevance:**

In this cohort study, early-life interactions between human milk feeding and *B infantis*, among other bacteria, were associated with the infant fecal metabolome and childhood SBP, underscoring the potential importance of early-life nutrition-microbe interplay in cardiometabolic health.

## Key Points

**Question:**

Does human milk feeding interact with infant gut microbes, particularly *Bifidobacterium longum* subsp *infantis *and other milk-degrading microbes, in association with infant fecal metabolome and childhood blood pressure?

**Findings:**

In this cohort study of 1324 Canadian children, any human milk feeding, compared with no human milk feeding, at age 3 months was associated with lower child systolic blood pressure at ages 3 and 5 years among infants harboring *B infantis* at age 3 months, but not among infants who did not harbor *B infantis*. An interaction was also observed between human milk feeding and several other microbes at age 3 months (but not age 12 months), including other species of *Bifidobacterium*, in relation to child blood pressure and fecal metabolites.

**Meaning:**

These findings suggest that early-life interactions between human milk feeding and infant gut microbes may shape the infant fecal metabolome and child blood pressure.

## Teaser

This cohort study investigates associations of human milk feeding and infant gut microbes, including *Bifidobacterium longum *subsp* infantis*, with infant fecal metabolites and childhood systolic blood pressure.

## Introduction

High blood pressure (BP) in childhood is an increasing but underrecognized problem. Recent estimates among children aged 6 years and older indicate that 2% to 5% have hypertension and 13% to 18% have elevated BP,^1^ but BP in early childhood (age ≤5 years) has received less research attention. Although hypertension may be rarely diagnosed at these early ages, higher BP levels in childhood track into adulthood,^2,3^ where hypertension is the leading modifiable risk factor for cardiovascular disease.^4^ Despite its considerable importance to public health, early-life risk factors for high childhood BP remain understudied, which limits opportunities for primary prevention.

Human milk feeding and the infant gut microbiome represent modifiable early-life factors associated with child BP.^5,6,7,8,9,10,11,12^ There is evidence that the health benefits of human milk may vary by infant gut microbiome composition. For example, infants lacking the bacterium *Bifidobacterium longum *subsp* infantis* (*B infantis*) may not fully benefit from human milk, as *B infantis* possesses 1 of the largest known gene clusters (43 genes) that allow it to use otherwise indigestible human milk oligosaccharides (HMOs)^13^—the third most abundant component of human milk—and carbon and energy sources and to bioconvert HMOs into BP-lowering metabolites, like acetate.^14^ However, to our knowledge, no human studies have examined the interactions between breastfeeding and gut microbes at the species or strain level with respect to child BP.

Germ-free murine models demonstrate causal links between the gut microbiome and BP,^15^ as well as the role of microbially produced acetate in regulating BP via G protein coupled receptors.^16^ However, longitudinal studies in children are needed to understand microbiome-metabolome-BP links, as findings in mice may not be translatable to humans, particularly children, due to differences in microbes, metabolic pathways, and nutrition.^17^

To address these research gaps, we used data from a large longitudinal birth cohort with rich data on infant fecal microbial metagenomics, fecal metabolomics, and repeated child BP measurements. We focused our investigation on how the interaction between human milk feeding and the infant gut microbiome, particularly *B infantis*, is prospectively associated with metabolites and childhood BP. We hypothesized that the BP-lowering effect of human milk is greater among infants harboring *B infantis*. We focused on the gut microbiome and metabolome in infancy because they are highly modifiable in early life but become relatively stable later in childhood.^18,19,20^ If these early-life exposures are associated with subsequent BP, they may represent promising targets for early prevention.

## Methods

### Study Design and Population

This cohort study was conducted as part of the Canadian Healthy Infant Longitudinal Development (CHILD) cohort study, a multicenter, prospective birth cohort that followed children from infancy through age 13 years.^21,22^ The study was approved by the local research ethics boards all study sites. All participating parents provided informed consent. This study followed the Strengthening the Reporting of Observational Studies in Epidemiology (STROBE) reporting guideline for cohort studies.

From 2008 to 2012, pregnant mothers were recruited from the general population in Vancouver, British Columbia; Edmonton, Alberta; Winnipeg and Morden/Winkler, Manitoba; and Toronto, Ontario. Infants born at 35 weeks of gestation or later without congenital abnormalities or respiratory distress syndrome were enrolled after informed consent was obtained from their parents.

### Shotgun Metagenomic Sequencing and Metabolite Quantification

Infant fecal samples were collected from diapers at approximate ages 3 months and 1 year.^23^ We conducted metagenomic sequencing following DNA extraction from fecal samples and used MetaPhlAn 4 version 4.1.0^24^ for microbiome taxonomic profiling. We estimated the relative abundances of *B longum* subspecies by adapting the approach by Ennis et al.^25^ Metabolites from the same sequenced fecal samples were measured using targeted nuclear magnetic resonance (NMR)^26,27^ and targeted liquid chromatography with tandem mass spectrometry (LC-MS/MS).^28^ To mitigate the impacts of outliers and increase comparability across assays, we performed a rank-based inverse normal transformation of metabolite levels separately for each time point (ie, 3 months and 1 year) and each assay (ie, NMR and LC-MS/MS) before downstream analyses. Detailed methods are described in the eMethods in Supplement 1.

### BP Measurement

Child BP was measured during 2 scheduled clinic visits at approximate ages 3 and 5 years using an automatic sphygmomanometer (Carescape Dinamap) on the right brachial artery with the child sitting quietly, feet flat on the floor, back and right arm supported^7^; approximately 80% of children attended both visits. BP measurement was repeated if the systolic BP (SBP) reading was greater than 105 mm Hg. We considered SBP instead of diastolic BP as the outcome of interest because it is more strongly associated with adult hypertension and cardiovascular disease.^29,30^ We calculated age-, sex-, and height-specific SBP percentiles based on the 2017 American Academy of Pediatrics Clinical Practice Guideline.^31^ We excluded 68 children with SBP readings exceeding 4 SD from the mean or with SBP percentiles not computable due to missing or extreme height values (eFigure 1 in Supplement 1).

### Feeding Status and Other Covariates Assessment

Parents self-reported feeding status at fecal sample collection (ie, ages 3 months and 1 year).^7^ We categorized human milk feeding status at 3 months as exclusive (human milk only without any other fluids or solid foods since birth), mixed (human milk plus any other fluids or solid foods), or none (no human milk). We categorized human milk feeding status at 1 year as any or none, as by this age few infants were exclusively human milk–fed, solid foods had been introduced in nearly all infants, and some had begun consuming cow’s milk. Maternal race and ethnicity, age at delivery, highest educational achievement, prenatal (by 18 weeks of gestation) and intrapartum antibiotic use, and hypertension (both chronic and during pregnancy) were self-reported at enrollment or delivery. Race and ethnicity were categorized as Asian, White, or other (eg, Black, Hispanic, Indigenous, Middle Eastern, multiracial, or other). Race and ethnicity were self-reported and collected to characterize the sociodemographic composition of the cohort and to provide context for the interpretation and generalizability of the results. We calculated maternal prepregnancy body mass index (BMI) based on measured height and self-reported prepregnancy weight. Information on delivery mode, child sex, and birth weight was extracted from medical records. We measured child height and weight at ages 3 and 5 years.

### Statistical Analysis

Because the exposures of interest in our study (ie, gut microbiome, metabolome, and feeding practices) change rapidly during infancy, we constructed models separately for exposures measured at ages 3 months and 1 year to evaluate potential critical windows. The outcome, SBP, was measured at ages 3 and 5 years and was analyzed as a repeated outcome using mixed-effects models with random intercepts for participants because results for SBP at the 2 time points were largely consistent when examined separately. When model structures did not permit repeated outcomes, SBP at ages 3 and 5 years was analyzed in separate models. Study site was additionally accounted for using a separate random intercept. As all exposures were assessed prior to the outcome, analyses reflect a prospective evaluation of infant exposures in relation to later SBP. This modeling structure was applied across all analyses.

We examined associations of *Bifidobacterium* species and human milk feeding status in infancy with childhood SBP. We focused on *Bifidobacterium* species present in at least 10% of samples (eTable 1 in Supplement 1): *B adolescentis*, *B animalis*, *B bifidum*, *B breve*, *B dentium*, *B longum* (including subsp* longum*, subsp* infantis*, and subsp unclassified), and *B pseudocatenulatum*. For each species, we modeled both presence or absence and centered log ratio (CLR)–transformed relative abundance. We obtained *P* for interaction between *Bifidobacterium* species and human milk feeding using likelihood ratio tests, defining a significant interaction at *P* < .10, given lower statistical power for interaction tests.^32^

Given our a priori interest in the interaction of *B infantis* and human milk feeding, we evaluated their joint associations with overall microbial community composition quantified by Bray-Curtis distance using permutational analysis of variance and visualized the results using principal coordinate analysis plot. We used the Bray-Curtis distance because it incorporates both presence or absence and relative abundance of taxa, thus providing a balanced measure of community composition.^33^ Furthermore, we examined the interaction between *B infantis* and feeding status in relation to metabolites produced by the degradation of human milk by *B infantis*, focusing on acetate and other short-chain fatty acids (SCFAs; eg, butyrate, propionate, valerate, isobutyrate, and isovalerate) and indolelactic acid.^34,35^

We used elastic net regression to identify infant fecal metabolites associated with SBP at ages 3 or 5 years, overall and stratified by human milk feeding status. We used SBP percentile residuals as the dependent variables and accounted for study site and confounding variables. We applied 10-fold nested cross-validation with 10-fold inner cross-validation and considered metabolites selected in more than 50% of outer folds as SBP-related. We calculated the mean for the coefficients for each selected metabolite obtained across folds as the final estimates. We additionally examined interactions between *B infantis* and human milk feeding in relation to these SBP-related metabolites. Lastly, we evaluated associations of non-*Bifidobacterium* microbes with SBP, non-*Bifidobacterium* microbes with SBP-related metabolites, and microbial diversity with SBP (eMethods in Supplement 1).

All models adjusted for maternal age at delivery, prepregnancy BMI, maternal educational achievement, maternal hypertension, antibiotic use during pregnancy, delivery mode, child sex, birth weight, and age and feeding status at fecal sample collection. We additionally adjusted for time between sample collection and freezing in statistical analyses involving metabolites to improve estimate precision, following the approach used in a previous published CHILD study.^28^ Sensitivity analyses included adding gestational age, maternal smoking during pregnancy, child BMI at BP measurement in adjustment, and repeating elastic net regression separately among metabolites quantified by NMR and LC-MS/MS. *P* values were 2-sided, and statistical significance was set at *P* ≤ .05, unless otherwise noted. We conducted all analyses in R software version 4.4.1 (R Project for Statistical Computing) (eMethods in Supplement 1). Data were analyzed from January to December 2024.

## Results

### Participant Characteristics

Our study included 1324 children (610 [46.1%] girls; 982 children [74.2%] delivered vaginally; mean [SD] maternal age at delivery, 33.3 [4.5] years) (eFigure 1 in Supplement 1; Table). There were 187 Asian mothers (14.1%), 980 White mothers (74.0%), and 157 mothers (11.9%) of other race or ethnicity. Most mothers had a college degree (979 mothers [73.9%]) or higher (258 mothers [19.5%]). Hypertension (chronic or during pregnancy) was uncommon (110 mothers [8.3%]), and 635 mothers (48.0%) used antibiotics by 18 weeks of gestation or intrapartum. At age 3 months, most infants received human milk (799 infants [60.4%] exclusively; 349 infants [26.4%] mixed), and 611 infants (47.3%) were still receiving any human milk at 1 year (eTable 2 in Supplement 1). Infants were more likely to receive human milk for a longer duration if they were born to mothers with higher educational achievement, lower prepregnancy BMI, and older maternal age at delivery. The mean (SD) SBP percentile was 74.6 (21.3) at age 3 years and 73.4 (22.0) at age 5 years.

**Table.** Characteristics of the Study Population

Characteristic	Individuals, No (%)			
	Overall (n = 1324)	Human milk feeding status at age 3 mo		
		Exclusive (n = 799)	Mixed (n = 349)	None (n = 174)
Maternal characteristics				
Race and ethnicity				
Asian	187 (14.1)	106 (13.3)	55 (15.8)	25 (14.4)
White	980 (74.0)	606 (75.8)	249 (71.3)	124 (71.3)
Other^a^	157 (11.9)	87 (10.9)	45 (12.9)	25 (14.4)
Highest education				
≤High school	87 (6.6)	33 (4.1)	19 (5.4)	35 (20.1)
College and university level	979 (73.9)	586 (73.3)	262 (75.1)	129 (74.1)
Graduate	258 (19.5)	180 (22.5)	68 (19.5)	10 (5.7)
Prepregnancy BMI, mean (SD)	24.4 (5.1)	23.6 (4.2)	24.9 (5.4)	27.4 (7.0)
Age at delivery, mean (SD), y	33.3 (4.5)	33.6 (4.3)	33.7 (4.3)	31.4 (5.3)
Hypertension				
Chronic	65 (4.9)	34 (4.3)	19 (5.4)	12 (6.9)
During pregnancy	45 (3.4)	27 (3.4)	14 (4.0)	4 (2.3)
No	1214 (91.7)	738 (92.4)	316 (90.5)	158 (90.8)
Antibiotic use				
Prenatal by 18 wk of gestation	42 (3.2)	27 (3.4)	11 (3.2)	4 (2.3)
Intrapartum	593 (44.8)	355 (44.4)	159 (45.6)	79 (45.4)
No	689 (52.0)	417 (52.2)	179 (51.3)	91 (52.3)
Child’s characteristics				
Sex				
Boy	714 (53.9)	410 (51.3)	214 (61.3)	89 (51.1)
Girl	610 (46.1)	389 (48.7)	135 (38.7)	85 (48.9)
Delivery mode				
Vaginal	982 (74.2)	604 (75.6)	252 (72.2)	124 (71.3)
Cesarean section with labor	180 (13.6)	114 (14.3)	42 (12.0)	24 (13.8)
Cesarean section without labor	162 (12.2)	81 (10.1)	55 (15.8)	26 (14.9)
Birth weight, mean (SD), kg	3.5 (0.5)	3.5 (0.5)	3.4 (0.4)	3.5 (0.5)
Gestational age, mean (SD), wk	39.6 (1.3)	39.7 (1.3)	39.4 (1.5)	39.5 (1.2)
Anthropometry and BP measures				
At age 3 y				
BMI, mean (SD)	16.3 (1.3)	16.2 (1.3)	16.3 (1.3)	16.7 (1.7)
SBP percentile, mean (SD)	74.6 (21.3)	74.1 (21.5)	75.1 (21.2)	75.9 (20.0)
DBP percentile, mean (SD)	80.6 (14.7)	79.9 (15.0)	81.7 (14.7)	81.6 (13.6)
At age 5 y				
BMI, mean (SD), kg/m^2^	15.8 (1.5)	15.6 (1.4)	15.8 (1.4)	16.3 (1.8)
SBP percentile, mean (SD)	73.4 (22.0)	73.3 (21.7)	72.9 (22.6)	74.8 (21.9)
DBP percentile, mean (SD)	65.3 (19.1)	64.7 (19.1)	64.2 (18.7)	70.7 (18.8)

Abbreviations: BMI, body mass index (calculated as weight in kilograms divided by height in meters squared); DBP, diastolic blood pressure; SBP, systolic blood pressure.

^a^ Including Black, Hispanic, Indigenous, Middle Eastern, multiracial, or other.

### Interaction of* Bifidobacterium* and Human Milk at Age 3 Months in Association With SBP

**Figure 1.**
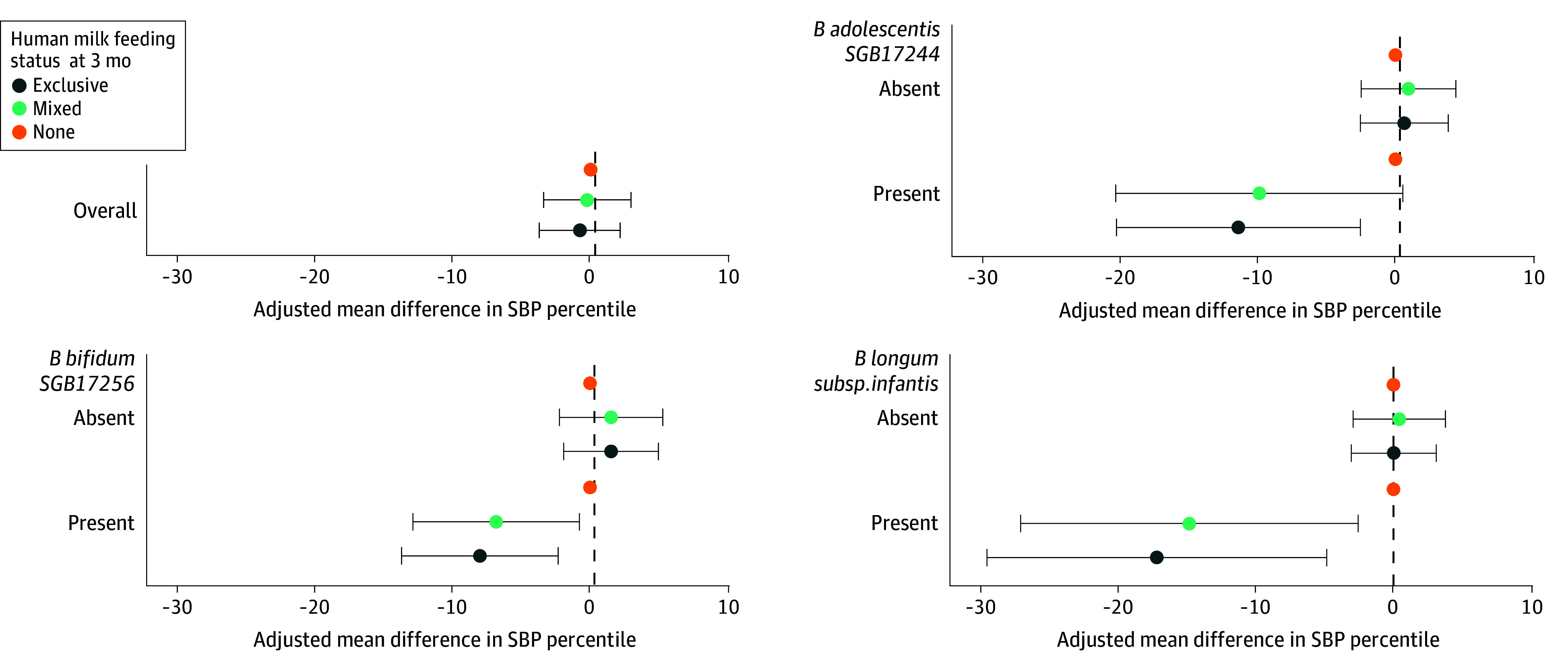
Associations of Human Milk Feeding Status at Age 3 Months With Systolic Blood Pressure (SBP) Percentile in Childhood, Overall and Stratified by Presence or Absence of Selected *Bifidobacterium* Species Estimates are from mixed-effect linear regression with SBP percentiles measured at ages 3 and 5 years, modeled as a repeated outcome. Models adjusted for maternal age at delivery (continuous), prepregnancy body mass index (continuous), educational achievement (≤high school, college and university level, graduate degree), hypertension (chronic, during pregnancy, no), antibiotic use (prenatal, intrapartum, no), delivery mode (vaginal, cesarean section with labor, cesarean section without labor), child sex (boy, girl), birth weight (continuous), and exact age in months (continuous) at fecal sample collection, with study site (Edmonton, Toronto, Vancouver, Winnipeg) as a random intercept. Results shown reflect associations of human milk feeding status at age 3 months with SBP percentile, stratified by selected *Bifidobacterium* species for which statistically significant associations were observed. Results stratifying by the remaining *Bifidobacterium* species at age 3 months and by all *Bifidobacterium* species at age 1 year are provided in eTable 2 in Supplement 1.

The association between human milk feeding at 3 months and childhood SBP differed by the presence or absence of *B infantis*, *B adolescentis*, or *B bifidum* (Figure 1; eTable 3 in Supplement 1). For example, among infants harboring *B infantis* at age 3 months, mixed (difference, −14.81 [95% CI, −27.05, −2.56] percentiles) or exclusive (difference, −17.16 [95% CI, −29.48 to −4.83] percentiles) human milk feeding at 3 months was associated with lower SBP percentile, whereas no association was observed among infants without *B infantis*. In the reverse stratification, presence or absence (eTable 4 in Supplement 1) or CLR-transformed relative abundance (eTable 5 in Supplement 1) of *B infantis* and *B bifidum* at age 3 months were not associated with childhood SBP when infants were mixed-fed or exclusively human milk–fed at age 3 months. However, among infants who did not receive human milk at age 3 months, both the presence and higher CLR-transformed relative abundance of *B infantis* and *B bifidum* at age 3 months were associated with higher SBP. No significant associations or interactions were observed for analyses of *Bifidobacterium* and feeding measured at age 1 year.

### Collective Association of* B infantis* and Human Milk Feeding With Fecal Microbiome

At age 3 months, Bray-Curtis distances revealed significant differences in gut microbial composition by *B infantis* presence or absence (eFigure 2 in Supplement 1), with the largest effects in exclusively human milk–fed infants (*R*^2^ = 12.60%; *P* = .001), followed by mixed-fed infants (*R*^2^ = 6.61%; *P* = .001), and no significant separation in infants not having human milk (*R*^2^ = 0.67%; *P* = .27). Human milk feeding alone explained less but still significant variation in microbial composition at ages 3 months (*R*^2^ = 1.08%; *P* = .001) and 1 year (*R*^2^ = 1.16%; *P* = .001) (eFigure 2 in Supplement 1). The association of human milk feeding was more pronounced among infants with *B infantis* vs those without at age 3 months (*R*^2^ = 5.23%; *P* = .003 vs *R*^2^ = 1.05%; *P* = .001) than at age 1 year (*R*^2^ = 1.66%; *P* = .001 vs *R*^2^ = 0.73%; *P* = .001).

### Fecal Metabolites Associated With SBP

Overall, 20 metabolites at age 3 months and 11 metabolites at age 1 year were significantly associated with childhood SBP, with most associations observed with SBP at age 5 years (eTable 6 in Supplement 1). For metabolites measured at age 3 months, the strongest positive associations with SBP at 5 years were observed for CE(20:0) (lipid), succinate, creatinine, PC ae C30:1 (lipid), and N-Acetyl-Asp (acetylated amino acid). In contrast, PC ae C40:4, Cer(d18:1/24:0), DG(16:1_20:0), CE(18:1), and CE(18:1) (all lipids) showed the strongest negative associations with SBP at age 5 years. For metabolites measured at age 1 year, none were associated with SBP at age 3 years; PC ae C44:4 (lipid) had the strongest positive association with SBP at age 5 years, while CE(18:3) (lipid) had the strongest negative association. Feeding-specific associations between metabolites and SBP were also identified (eTable 6 in Supplement 1). For example, succinic acid at age 3 months was positively associated with SBP at age 5 years among infants who did not receive human milk at age 3 months but not among infants who did.

### Interaction of *B infantis* and Human Milk in Association With SBP-Related Metabolites

**Figure 2.**
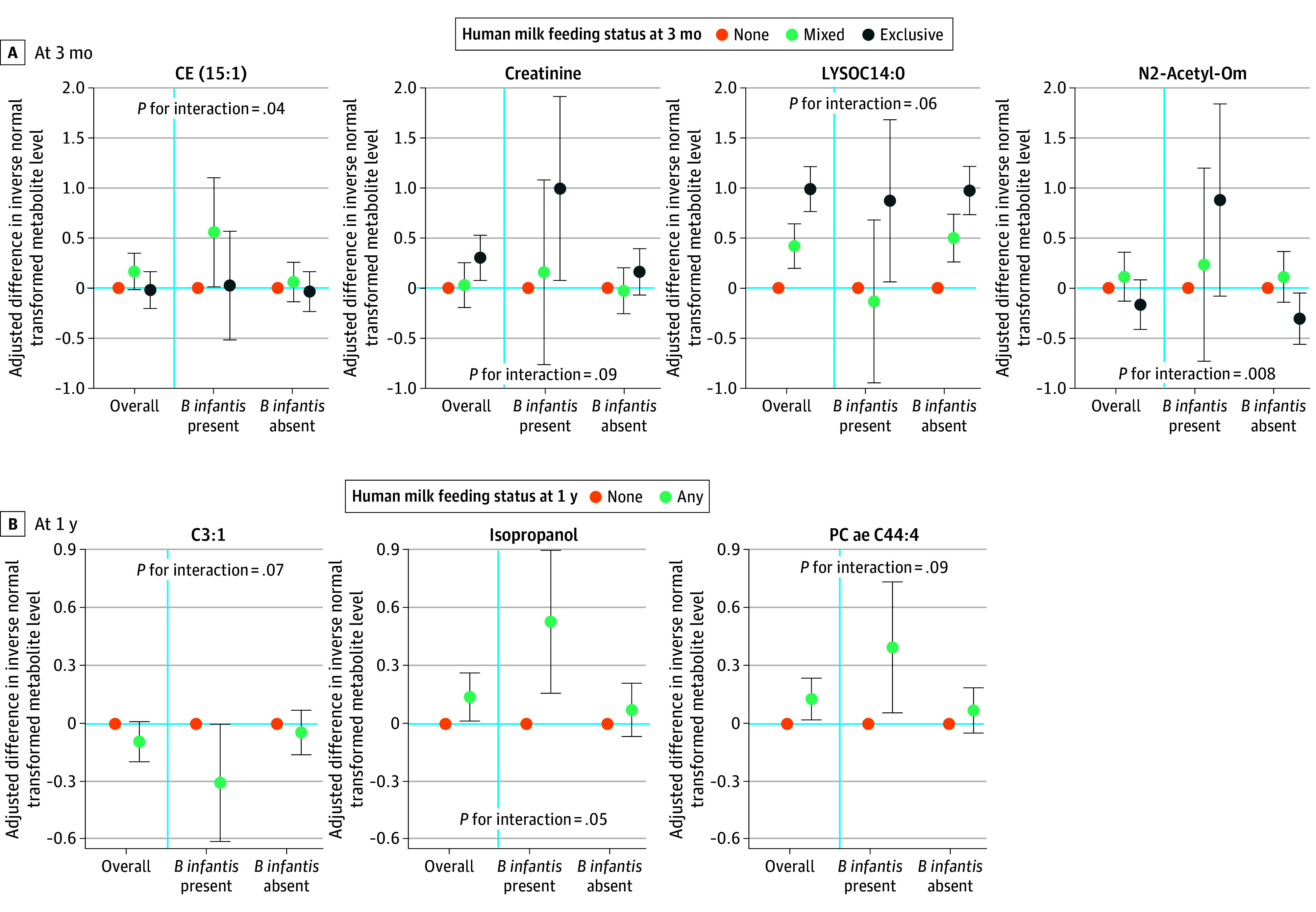
Significant Interactions Between *B infantis* and Human Milk Feeding Status in Relation to Systolic Blood Pressure–Associated Fecal Metabolites Estimates are based on mixed-effect linear regressions with metabolite levels transformed by rank-based inverse normal transformation as the outcome. Systolic blood pressure–associated metabolites are identified by elastic net regression in the overall sample or within each stratum of feeding status. Significant interactions are shown here and eFigure 3 in Supplement 1. Results of all metabolites are in eTable 6 and eTable 7 in Supplement 1. Models adjusted for maternal age at delivery (continuous), prepregnancy body mass index (continuous), educational achievement (≤high school, college and university level, graduate degree), hypertension (chronic, during pregnancy, no), antibiotic use (prenatal, intrapartum, no), delivery mode (vaginal, cesarean section with labor, cesarean section without labor), child sex (boy, girl), birth weight (continuous), exact age in months (continuous) at fecal sample collection, and time between sample collection and freezing (hours), with study site (Edmonton, Toronto, Vancouver, Winnipeg) as a random intercept.

The* B infantis*-human milk interaction was associated with several SBP-related metabolites (Figure 2; eFigure 3, eTable 7, and eTable 8 in Supplement 1), including CE(15:1) (lipid), creatinine, LYSOC14:0 (lipid), and N2-Acetyl-Orn (acetylated amino acid) at age 3 months, and C3:1 (acylcarnitine), isopropanol, and PC ae C44:4 (lipid) at age 1 year. For example, at age 3 months, exclusive human milk feeding was associated with higher creatinine (change in inverse normal transformed value, 1.00 [95% CI, 0.08 to 1.92] SDs) only among infants harboring *B infantis*. As for SCFAs (eTable 9 and eTable 10 in Supplement 1), at 3 months, there was no statistically significant difference in acetate in infants harboring *B infantis* who were exclusively human milk–fed (change in inverse normal transformed value, 0.71 [95% CI, −0.35 to 1.78] SDs) or mixed-fed (change in inverse normal transformed value, 0.13 [95% CI, −0.90 to 1.15] SDs) compared with those who did not receive human milk. No similar trends were observed with acetate at age 1 year or with other SCFAs.

### Sensitivity Analyses and Other Findings

Further adjustment for gestational age, maternal smoking during pregnancy (prevalence: 103 mothers [7.8%]), or child BMI at BP measurement did not alter the results, and overall conclusions remained unchanged. SBP-related metabolites identified in the assay-specific elastic net regressions (eTable 11 in Supplement 1) were highly consistent with those from the main combined-assay analysis (eResults in Supplement 1).

Furthermore, we found that a small number of non-*Bifidobacterium* microbes in infancy (eg, *Eggerthella lenta*, *Veillonella dispar*) also showed feeding-dependent associations with childhood SBP (eTable 12 and eTable 13 in Supplement 1). Additionally, several SBP-related microbes correlated with SBP-related metabolites (eTable 14 and eFigure 4 in Supplement 1). Finally, infant fecal microbial diversity metrics showed largely null associations with childhood SBP (eTable 15 and eFigure 5 in Supplement 1).

## Discussion

In this population-based cohort study of 1324 Canadian children, presence of key gut microbes at age 3 months, including *B infantis* and some other Bifidobacteria, interacted with human milk feeding in association with the infant fecal metabolome and childhood SBP. These findings build on our earlier research in a Danish cohort of 526 children, where breastfeeding combined with certain *Bifidobacterium* microbes (not identified to the species level) was associated with lower SBP at ages 3 and 6 years.^12^ In this study, metagenomic sequencing allowed us to pinpoint *B infantis* as a key *Bifidobacterium* species in this interaction. Taken together, these findings provide additional evidence that the cardiovascular benefits of human milk may depend on presence of HMO-degrading microbes, like *B infantis*. Furthermore, geographic variability in the prevalence of *B infantis*, which ranges from 0% to 83.6% in breastfed infants during the first 2 months,^36^ may partly explain inconsistent findings in prior studies on associations of breastfeeding with childhood SBP, where both protective^5,6,7,8^ and null associations^9,10,11^ have been reported.

The interplay among *B infantis*, human milk feeding, and childhood SBP appeared to be age-specific, emerging only when *B infantis* and feeding status were assessed in infants at age 3 months, when human milk remained the primary nutritional source, rather than at age 1 year. Metabolite byproducts of HMO metabolism may contribute to this pattern. Consistent with a 2024 ex vivo study,^35^ infants who received human milk and harbored *B infantis* had higher acetate levels at age 3 months, although acetate itself was not associated with childhood SBP in our study. Other metabolites also showed age-specific patterns in association with *B infantis*, human milk feeding, and SBP. Succinate, a microbial metabolite associated with adverse cardiometabolic traits in adults,^37^ was positively associated with SBP in infants who did not receive human milk, but not among those who did, in whom *B infantis* was associated with lower succinate levels, suggesting a potential mitigating role of *B infantis*. Creatinine, a marker of muscle metabolism and kidney function, has been associated with adverse cardiovascular outcomes in young adults^38^ and is present in both human milk and formula, presumably as a nitrogen source for gut microbiota, like *Bifidobacterium*.^39^ We found higher creatinine in exclusively human milk–fed infants harboring *B infantis* at age 3 months, whereas an Italian neonatal study reported a negative association.^40^ This discrepancy may reflect age-related metabolic changes or enhanced protein turnover, or kidney function mediated by *B infantis* in exclusively human milk–fed infants.^41^

The correlations between SBP-related microbes and SBP-related metabolites support the hypothesis that metabolites may partly underlie the microbiome-BP association. Many infant fecal metabolites identified in our study align with metabolite findings from other sample types in infants and children. For example, infant fecal acylcarnities (C18, C16OH, C14, C2), key metabolites in fatty acid oxidation, were positively associated with childhood SBP, consistent with a cord blood study in children.^42^ Infant fecal lipids, such as cholesteryl esters, phosphatidylcholines, and N-acetylated amino acids, were prospectively associated with childhood SBP in our study, echoing plasma metabolite findings in adolescents.^43^ Although cord blood, plasma, and feces reflect different physiological compartments with distinct metabolic processing, the recurrence of similar metabolic pathways across these matrices strengthens the biological plausibility of our results, suggesting that fatty acid oxidation and lipid and amino acid metabolism may be relevant BP-related pathways from infancy through adolescence.

Our study is strengthened by our longitudinal design, which included assessment of both microbial metagenomics and metabolomics at 2 time points in infancy, ascertainment of BP at 2 time points in childhood, and collection of and adjustment for multiple potential confounders from pregnancy through childhood. This design allowed us to characterize early-life microbial and metabolic profiles across infancy and examine their prospective associations with SBP repeatedly measured in childhood.

### Limitations

This study has some limitations. First, BP was generally measured once at the 3- and 5-year visits, introducing potential nondifferential measurement error that likely biased observed associations toward the null. Second, as an observational study, causal inference is limited; although we controlled for multiple maternal and child factors, residual and unmeasured confounding remains possible. Third, because mothers in this cohort were relatively highly educated, the generalizability of our findings to more socioeconomically diverse populations may be limited, and replication in other settings is warranted. Fourth, we were unable to examine associations between microbiome or metabolome profiles measured closer to BP assessment in childhood due to data unavailability; this represents a separate research question for future studies.

## Conclusions

In this cohort study of 1324 Canadian children, we identified early-life interactions between human milk feeding and several gut microbes, including *B infantis*, with respect to the infant metabolome and childhood SBP. Our results support the hypothesis that the presence of HMO-degrading microbes, like *B infantis,* in early life enhances the SBP-lowering associations of human milk. Human milk feeding also modified associations of gut microbes, including *Eggerthella lenta* and *Veillonella dispar*, with SBP, suggesting microbes may have differential cardiovascular effects depending on nutrient availability.

Because the gut microbiome and fecal metabolome continue to evolve beyond infancy, our findings should be interpreted as prospective, observational associations of early-life microbial and metabolic exposures with subsequent SBP. These findings highlight translational potential opportunities for precision nutrition strategies aimed at optimizing early gut microbiome to support cardiovascular health. Factors arising after infancy, such as later child diet, may modify these associations and warrant further investigation.

## Notes

Supplement 1. eMethods. eResults. eReferences eFigure 1. Flow diagram of study design and sample selection eFigure 2. *B infantis* colonization and human milk feeding status shape infant microbiome composition eFigure 3. Associations between *B infantis* presence/absence and SBP-related fecal metabolites, overall and by human milk feeding status (significant interactions) eFigure 4. Correlation matrix between microbes (other than *Bifidobacterium*) and metabolites associated with SBP eFigure 5. Associations of Bray-Curtis distances at 3 month and 1 year with SBP percentiles at 3 and 5 years, overall and stratified by human milk feeding status eTable 1. Prevalence and relative abundance of *Bifidobacterium* Species at 3 months and 1 year eTable 2. Characteristics of study population from the CHILD cohort study by human milk feeding status at 1 year eTable 3. Associations between human milk feeding status in infancy and SBP percentile in childhood, overall and stratified by presence/absence of Bifidobacterium species eTable 4. Associations between the presence/absence of *Bifidobacterium* species in infancy with SBP percentile in childhood, overall and stratified by human milk feeding status eTable 5. Associations between relative abundance of *Bifidobacterium* species in infancy with SBP percentile in childhood, overall and stratified by human milk feeding status eTable 6. Fecal metabolites in infancy that are associated with SBP percentile in childhood, overall and within each stratum of human milk feeding status eTable 7. Associations between human milk feeding status and SBP-related fecal metabolites at 3 months and 1 year, overall and by presence/absence of *B infantis* eTable 8. Associations between presence/absence of *B infantis* and SBP-related fecal metabolites at 3 months and 1 year, overall and by human milk feeding status eTable 9. Associations of human milk feeding status with indolelactic acid and short chain fatty acids at 3 months and 1 year, overall and by presence/absence of *B infantis* eTable 10. Associations of presence/absence of *B infantis* with indolelactic acid and short chain fatty acids at 3 months and 1 year, overall and by human milk feeding status eTable 11. Fecal metabolites in infancy that are associated with SBP percentile in childhood, overall and within each stratum of human milk feeding status, identified by assay-specific elastic net regression eTable 12. Differentially abundant fecal microbes at 3 month and 1 year in association with SBP percentiles at 3 and 5 years, identified by MaAsLin2 eTable 13. Associations between relative abundances of differentially abundant microbes in infancy identified by MaAsLin2 and SBP percentile in childhood, overall and stratified by human milk feeding status eTable 14. Correlation matrix between SBP-related microbes and SBP-related metabolites at 3 months and 1 year eTable 15. Association between Shannon index in infancy and SBP percentile in childhood, overall and stratified by human milk feeding status

Supplement 2. Data Sharing Statement
