# Supplementary material for: Human Milk and Infant Gut Microbiome in Association With Infant Fecal Metabolome and Child Blood Pressure
Source: JAMA Netw Open. 2026 Feb 13;9(2):e2559385. doi: 10.1001/jamanetworkopen.2025.59385 (PMC12905658; doi:10.1001/jamanetworkopen.2025.59385)
Supplement: Supplement 1. — eMethods. eResults. eReferences eFigure 1. Flow diagram of study design and sample selection eFigure 2. B infantis colonization and human milk feeding status shape infant microbiome composition eFigure 3. Associations between B infantis presence/absence and SBP-related fecal metabolites, overall and by human milk feeding status (significant interactions) eFigure 4. Correlation matrix between microbes (other than Bifidobacterium) and metabolites associated with SBP eFigure 5. Associations of Bray-Curtis distances at 3 month and 1 year with SBP percentiles at 3 and 5 years, overall and stratified by human milk feeding status eTable 1. Prevalence and relative abundance of Bifidobacterium Species at 3 months and 1 year eTable 2. Characteristics of study population from the CHILD cohort study by human milk feeding status at 1 year eTable 3. Associations between human milk feeding status in infancy and SBP percentile in childhood, overall and stratified by presence/absence of Bifidobacterium species eTable 4. Associations between the presence/absence of Bifidobacterium species in infancy with SBP percentile in childhood, overall and stratified by human milk feeding status eTable 5. Associations between relative abundance of Bifidobacterium species in infancy with SBP percentile in childhood, overall and stratified by human milk feeding status eTable 6. Fecal metabolites in infancy that are associated with SBP percentile in childhood, overall and within each stratum of human milk feeding status eTable 7. Associations between human milk feeding status and SBP-related fecal metabolites at 3 months and 1 year, overall and by presence/absence of B infantis eTable 8. Associations between presence/absence of B infantis and SBP-related fecal metabolites at 3 months and 1 year, overall and by human milk feeding status eTable 9. Associations of human milk feeding status with indolelactic acid and short chain fatty acids at 3 months and 1 year, overall and by presence/absence of B i [file jamanetwopen-e2559385-s001.pdf]

Supplemental Online Content

Liu T, Petersen C, Zhao N, et al. Human Milk and Infant Gut Microbiome in Association With Infant Fecal Metabolome and Child Blood Pressure. JAMA Netw Open. 2026;9(2):e2559385. doi:10.1001/jamanetworkopen.2025.59385

eMethods.
eResults.
eReferences.
eFigure 1. Flow diagram of study design and sample selection
eFigure 2. *B infantis* colonization and human milk feeding status shape infant microbiome composition
eFigure 3. Associations between *B infantis* presence/absence and SBP-related fecal metabolites, overall and by human milk feeding status (significant interactions)
eFigure 4. Correlation matrix between microbes (other than Bifidobacterium) and metabolites associated with SBP
eFigure 5. Associations of Bray-Curtis distances at 3 month and 1 year with SBP percentiles at 3 and 5 years, overall and stratified by human milk feeding status
eTable 1. Prevalence and relative abundance of *Bifidobacterium* Species at 3 months and 1 year
eTable 2. Characteristics of study population from the CHILD cohort study by human milk feeding status at 1 year
eTable 3. Associations between human milk feeding status in infancy and SBP percentile in childhood, overall and stratified by presence/absence of *Bifidobacterium* species
eTable 4. Associations between the presence/absence of *Bifidobacterium* species in infancy with SBP percentile in childhood, overall and stratified by human milk feeding status
eTable 5. Associations between relative abundance of *Bifidobacterium* species in infancy with SBP percentile in childhood, overall and stratified by human milk feeding status
eTable 6. Fecal metabolites in infancy that are associated with SBP percentile in childhood, overall and within each stratum of human milk feeding status
eTable 7. Associations between human milk feeding status and SBP-related fecal metabolites at 3 months and 1 year, overall and by presence/absence of *B infantis*
eTable 8. Associations between presence/absence of *B infantis* and SBP-related fecal metabolites at 3 months and 1 year, overall and by human milk feeding status
eTable 9. Associations of human milk feeding status with indolelactic acid and short chain fatty acids at 3 months and 1 year, overall and by presence/absence of *B infantis*
eTable 10. Associations of presence/absence of *B infantis* with indolelactic acid and short chain fatty acids at 3 months and 1 year, overall and by human milk feeding status

eTable 11. Fecal metabolites in infancy that are associated with SBP percentile in childhood, overall and within each stratum of human milk feeding status, identified by assay-specific elastic net regression
eTable 12. Differentially abundant fecal microbes at 3 month and 1 year in association with SBP percentiles at 3 and 5 years, identified by MaAsLin2
eTable 13. Associations between relative abundances of differentially abundant microbes in infancy identified by MaAsLin2 and SBP percentile in childhood, overall and stratified by human milk feeding status
eTable 14. Correlation matrix between SBP-related microbes and SBP-related metabolites at 3 months and 1 year
eTable 15. Association between Shannon index in infancy and SBP percentile in childhood, overall and stratified by human milk feeding status

This supplemental material has been provided by the authors to give readers additional information about their work.

eMethods

Details on Fecal Sample Collection and Processing

Infant fecal samples were collected by trained research assistants at 3 months [16.6±4.9 (standard deviation; SD) weeks] during scheduled home visits and at 1 year (1.1±0.17 years) in an outpatient clinic.[1] Considering that infant stooling patterns vary, parents were asked to place a provided Tegaderm liner in the diaper starting at least three days before the visit to ensure that a sample could be collected before the visit. The diaper with stool was then kept in a sealed plastic bag in the refrigerator until transfer. If multiple samples were obtained, the most recent one was used. Upon transfer to the laboratory on ice in a cooler, samples were aliquoted into four 2mL cryovials using a stainless steel depyrogenated spatula and frozen at -80°C. The time between sample collection and freezing was recorded.

Details on Shotgun Metagenomic Sequencing and Bioinformatics

DNA was extracted from fecal samples using the MO Bio PowerSoil Pro automated system for high throughput on a QiaCube, with bead beating in 0.1mm glass bead plates, and quantified with the Quant-iT PicoGreen dsDNA Assay. Libraries were prepared using a procedure adapted from the Nextera Library Prep kit and sequenced either on an Illumina NextSeq with single-end 1x150 reads using a NextSeq 500/550 High Output v2 kit or an Illumina NovaSeq with single-end 1x100 reads. Low quality (Q-Score<30) and short-length (<50) DNA sequences were removed, and adapter sequences were trimmed using Cutadapt[2]. After removing human reads using KneadData (v0.10.0), cleaned reads were concatenated, and samples with ≥300,000 remaining reads were retained for further processing (**eFigure 1**). We used MetaPhlAn 4 (v4.1.0)[3] for taxonomic profiling. This tool maps metagenomic sequences, classifies them into various taxonomic levels including species and subspecies/strain levels, and calculates their relative abundances.[3] To quantify relative abundances of *B. longum* subspecies, we modified MetaPhlAn's latest database (mpa_vJun23_CHOCOPhlAnSGB_202307) to separate clade markers associated with individual *B. longum* subspecies by adapting the approach described in Ennis et al. (2024).[4]

Details on Metabolite Quantification and Transformation

Targeted nuclear magnetic resonance (NMR) analysis quantified 31 metabolites across 62 batches,[5,6] and targeted liquid chromatography with tandem mass spectrometry (LC-MS/MS) quantified 365 metabolites across 27 batches.[7] We excluded samples with no results due to insufficient samples or missing quality information, samples with a local outlier factor >6 as determined by their distance to the nearest neighbor, and metabolites with a prevalence of <20% (**eFigure 1**). Both NMR and LC-MS/MS profiling in CHILD were run with an extensive library of reference standards, yielding calibrated quantitative concentrations rather than raw signal intensities. Metabolite levels were normalized to stool water content and analyzed using the ratio (μmol/g). Levels below the limit of detection were assigned a value of half the minimum concentration. To mitigate the impacts of outliers and further improve comparability across assays we performed a rank-based inverse normal transformation for metabolite levels separately for each time point (i.e., 3 months, 1 year) and each assay (i.e., NMR, LC-MS/MS). This transformation yields a mean of 0 and SD of 1 for each metabolite, thereby improving comparability across assays. We estimated the batch effects on transformed metabolite levels using permutational multivariable analysis of variance (PERMANOVA) on Euclidean distances. Since batches accounted for only about 0.1% of the metabolite variability, no further correction for batch effects was performed. We modeled NMR and LC-MS/MS metabolites jointly to allow the elastic net regression to account for the cross-platform correlation structure of the metabolites when selecting features. A similar merged-analysis strategy of outputs from NMR and LC-MS/MS after pre-processing has been used previously in the CHILD cohort.[7]

Statistical Analysis on Microbes Other Than Bifidobacterium

We examined associations of human milk feeding status and non-*Bifidobacterium* microbes with SBP. Because analyses for these microbes were exploratory and involved hundreds of species, to appropriately account for the compositional and high-dimensional structure of microbiome data while controlling for false discovery rates (FDR) using the Benjamini-Hochberg method,[8] we used MaAsLin2[9] (with default options) to identify species associated with SBP at 3 or 5 years, separately, as a screening step. We tested microbes present in ⩾10% of samples. Significant microbes from MaAsLin2 were then analyzed using mixed-effect linear regression models in the same way as for *Bifidobacterium* species, with SBP modeled as a repeated outcome. We conducted these analyses both overall and stratified by feeding status. We quantified correlations between microbes (other than *Bifidobacterium*) and metabolites that were associated with SBP using Spearman correlation coefficients. We defined a significant correlation at a two-sided FDR-P<0.05.[8]

Statistical Analysis on Microbial Diversity Associations with SBP
We calculated the Shannon index[10], which indicates a combination of microbiome richness and evenness, as a measure of α diversity (i.e., within-individual diversity). We used Bray-Curtis,[11] which quantifies microbiome dissimilarity by considering presence/absence as well as abundances of microbes, as a measure of β diversity (i.e., between-individual diversity). We did not rarefy metagenomic sequencing for diversity estimation. We estimated the associations of Shannon index with SBP using mixed effect linear regression. We used PERMANOVA to assess the associations of Bray-Curtis distances with SBP and visualized these associations using PCoA plots.

Details on R packages Used in the Analysis
We used the *phyloseq*[12] package to manage microbiome data and make PCoA plots; *nlme*[13] and *lme4*[14] packages to conduct mixed-effects linear regressions; *microbiome*[15] package to perform CLR transformation of microbe relative abundance; *rbiom*[16] package to estimate Bray-Curtis distances; *vegan*[17] package to estimate Shannon index and perform PERMANOVA; *glmnet*[18,19] and *caret*[20] packages to employ elastic net regression with cross-validation; *Maaslin2*[21] package to identify species associated with SBP; *dbscan*[22] to quantify the distance between a metabolite and its nearest neighbor for outlier identification; and *ggplot2*[23] and *ggpubr*[24] packages for visualization.

eResults

Other Microbes and their Correlation with SBP-associated Metabolites
MaAsLin2 identified 7 microbes at 3 months and 4 microbes at 1 year that were associated with SBP at either 3 or 5 years (**eTable 11**), and some of these associations were modified by human milk feeding status (**eTable 12**). For example, higher relative abundance of *Eggerthella lenta* at 3 months was associated with lower SBP in mixed- or formula-fed infants but not in exclusively human milk-fed infants (*P-interaction*=0.007). Conversely, higher relative abundance of *Veillonella dispar* at 1 year was associated with higher SBP in formula-fed infants, but not in human milk-fed infants (*P-interaction*=0.026).

Metabolites and microbes associated with SBP in the same direction were often positively correlated (**eFigure 4**). For example, at 3 months histamine was negatively associated with SBP, positively correlated with *Veillonella dispar* (ρ=0.13, *FDR-P*<0.05), which was also negatively associated with SBP. Conversely, metabolites and microbes associated with SBP in opposite directions tended to be negatively correlated. For instance, creatinine, positively associated with SBP, was negatively correlated with *Eggerthella lenta* (ρ<-0.21, *FDR-P*<0.05), which was negatively associated with SBP.

Microbial Diversity and SBP
Overall, microbial α diversity, measured by Shannon index, was not associated with SBP percentiles (**eTable 14**). However, at 1 year, feeding status modified this association (*P-interaction*=0.052). Specifically, among formula-fed infants at 1 year, higher Shannon index was associated with lower SBP percentiles (-2.67 [95% CI: -5.04, -0.29]), while no association was found in human milk-fed infants (0.56 [95% CI: -1.72, 2.84]).

For microbial β diversity, Bray-Curtis distances at 3 months were significantly associated with SBP at 5 years (R^2=0.18%, *P*=0.046), Bray-Curtis distances at 1 year were significantly associated with SBP at 3 years (R^2=0.16%, *P*=0.039), and no evidence of modification by feeding status was found (**eFigure 5)**.

Assay-Specific Elastic Net Regression Results
In the main analysis combing metabolites quantified by NMR an LC-MS/MS, elastic net regression selected 75 metabolites (**eTable 5**), whereas the assay-specific sensitivity analysis selected 88 metabolites (**eTable 10**). Across all metabolite–SBP associations (3-month/1-year metabolites predicting 3-/5-year SBP), evaluated both overall and within human milk feeding categories, 41 metabolites were identified in both analyses. All overlapping metabolites had coefficients in the same direction, demonstrating strong robustness of the selected metabolites. Partial differences in the selected sets are expected, as running the elastic net separately for NMR and LC-MS/MS alters the underlying correlation structure and, consequently, feature selection.

References

1. Moraes TJ, Lefebvre DL, Chooniedass R, et al. The Canadian healthy infant longitudinal development birth cohort study: biological samples and biobanking. *Paediatr Perinat Epidemiol*. Jan 2015;29(1):84-92. doi:10.1111/ppe.12161
2. Martin M. Cutadapt removes adapter sequences from high-throughput sequencing reads. *EMBnetjournal*. 2011;17(1):10. doi:10.14806/ej.17.1.200
3. Blanco-Miguez A, Beghini F, Cumbo F, et al. Extending and improving metagenomic taxonomic profiling with uncharacterized species using MetaPhlAn 4. *Nat Biotechnol*. Nov 2023;41(11):1633-1644. doi:10.1038/s41587-023-01688-w
4. Ennis D, Shmorak S, Jantscher-Krenn E, Yassour M. Longitudinal quantification of Bifidobacterium longum subsp. infantis reveals late colonization in the infant gut independent of maternal milk HMO composition. *Nat Commun*. Jan 30 2024;15(1):894. doi:10.1038/s41467-024-45209-y
5. Drall KM, Tun HM, Morales-Lizcano NP, et al. Clostridioides difficile Colonization Is Differentially Associated With Gut Microbiome Profiles by Infant Feeding Modality at 3-4 Months of Age. *Front Immunol*. 2019;10:2866. doi:10.3389/fimmu.2019.02866
6. Bridgman SL, Malmuthuge N, Mandal R, et al. Childhood body mass index and associations with infant gut metabolites and secretory IgA: findings from a prospective cohort study. *Int J Obes (Lond)*. Sep 2022;46(9):1712-1719. doi:10.1038/s41366-022-01183-3
7. Hoskinson C, Dai DLY, Del Bel KL, et al. Delayed gut microbiota maturation in the first year of life is a hallmark of pediatric allergic disease. *Nat Commun*. Aug 29 2023;14(1):4785. doi:10.1038/s41467-023-40336-4
8. Benjamini Y, Hochberg Y. Controlling the false discovery rate: a practical and powerful approach to multiple testing. *Journal of the Royal statistical society: series B (Methodological)*. 1995;57(1):289-300.
9. Mallick H, Rahnavard A, McIver LJ, et al. Multivariable association discovery in population-scale meta-omics studies. *PLoS Comput Biol*. Nov 2021;17(11):e1009442. doi:10.1371/journal.pcbi.1009442
10. Shannon CE. A mathematical theory of communication. *The Bell system technical journal*. 1948;27(3):379-423.
11. Bray JR, Curtis JT. An ordination of the upland forest communities of southern Wisconsin. *Ecological monographs*. 1957;27(4):326-349.
12. McMurdie PJ, Holmes S. phyloseq: an R package for reproducible interactive analysis and graphics of microbiome census data. *PLoS One*. 2013;8(4):e61217. doi:10.1371/journal.pone.0061217
13. *nlme: Linear and Nonlinear Mixed Effects Models*. 2025. https://CRAN.R-project.org/package=nlme
14. Bates D, Mächler M, Bolker B, Walker S. Fitting linear mixed-effects models using lme4. *Journal of statistical software*. 2015;67:1-48.
15. *microbiome R package*. 2012-2019. https://bioconductor.org/packages/release/bioc/html/microbiome.html
16. *rbiom: Read/Write, Transform, and Summarize 'BIOM' Data*. 2021. https://CRAN.R-project.org/package=rbiom
17. *vegan: Community Ecology Package*. 2024. https://CRAN.R-project.org/package=vegan
18. Friedman J, Hastie T, Tibshirani R. Regularization Paths for Generalized Linear Models via Coordinate Descent. *J Stat Softw*. 2010;33(1):1-22.
19. Tay JK, Narasimhan B, Hastie T. Elastic Net Regularization Paths for All Generalized Linear Models. *J Stat Softw*. 2023;106:1-31. doi:10.18637/jss.v106.i01
20. Kuhn M. Building predictive models in R using the caret package. *Journal of statistical software*. 2008;28:1-26.
21. *MaAsLin 2: Multivariable Association in Population-scale Meta-omics Studies*. 2020. http://huttenhower.sph.harvard.edu/maaslin2
22. Hahsler M, Piekenbrock M, Doran D. dbscan: Fast density-based clustering with R. *Journal of Statistical Software*. 2019;91:1-30.
23. *ggplot2: Elegant Graphics for Data Analysis*. 2016. https://ggplot2.tidyverse.org
24. *ggpubr: 'ggplot2' Based Publication Ready Plots*. 2023. https://CRAN.R-project.org/package=ggpubr

eFigure 1. Flow diagram of study design and sample selection.

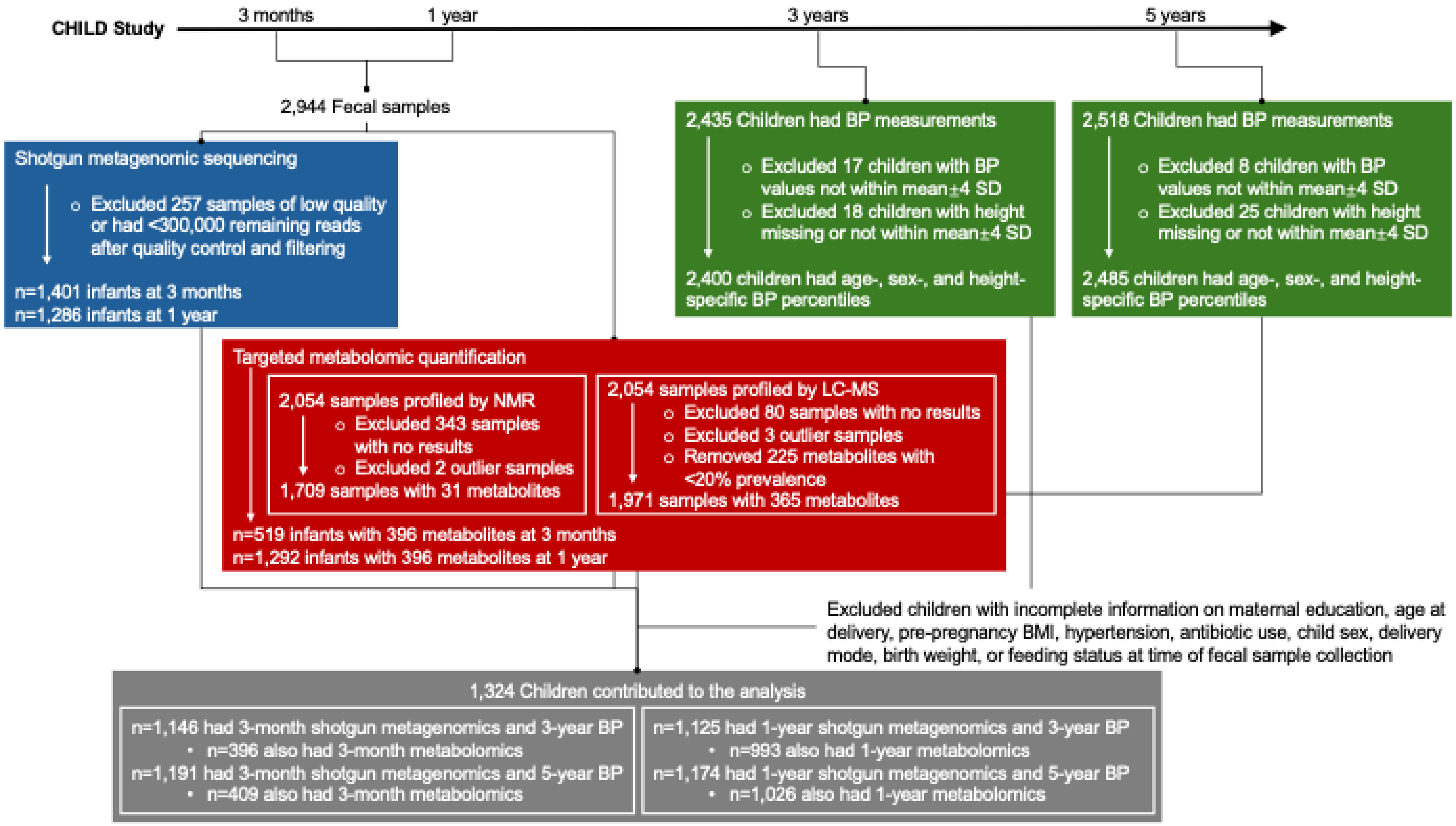

eFigure 2. *B. infantis* colonization and human milk feeding status shape infant microbiome composition.

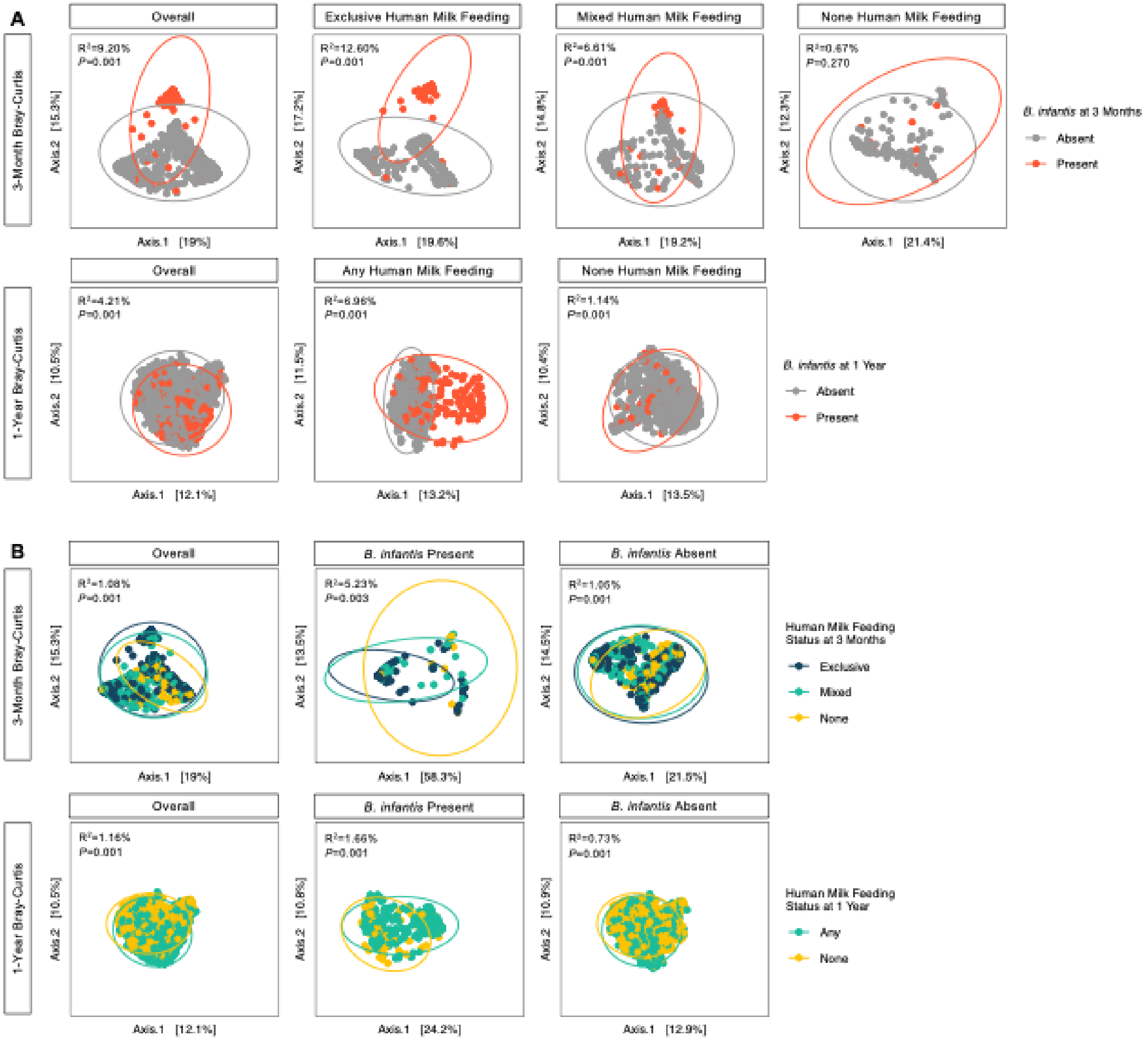

A shows the percentage of variation (R^2) in Bray-Curtis distances that are explained by *B .infantis* colonization, overall and stratified by feeding status. B shows the percentage of variation that are explained by feeding status, overall and stratified by the *B. infantis* colonization. Estimates are based on permutational multivariable analysis of variance (PERMANOVA). Models adjusted for maternal age at delivery (continuous), pre-pregnancy body mass index (continuous), educational achievement (high school or below, college and university level, graduate degree), hypertension (chronic, during pregnancy, no), antibiotic use (prenatal, intrapartum, no), delivery mode (vaginal, C-section with labor, C-section without labor), child sex (boy, girl), birth weight (continuous), and exact age in months (continuous) at fecal sample collection, with study site (Edmonton, Toronto, Vancouver, Winnipeg) as a random intercept.

eFigure 3. Associations between *B. infantis* presence/absence and SBP-related fecal metabolites, overall and by human milk feeding status (significant interactions).

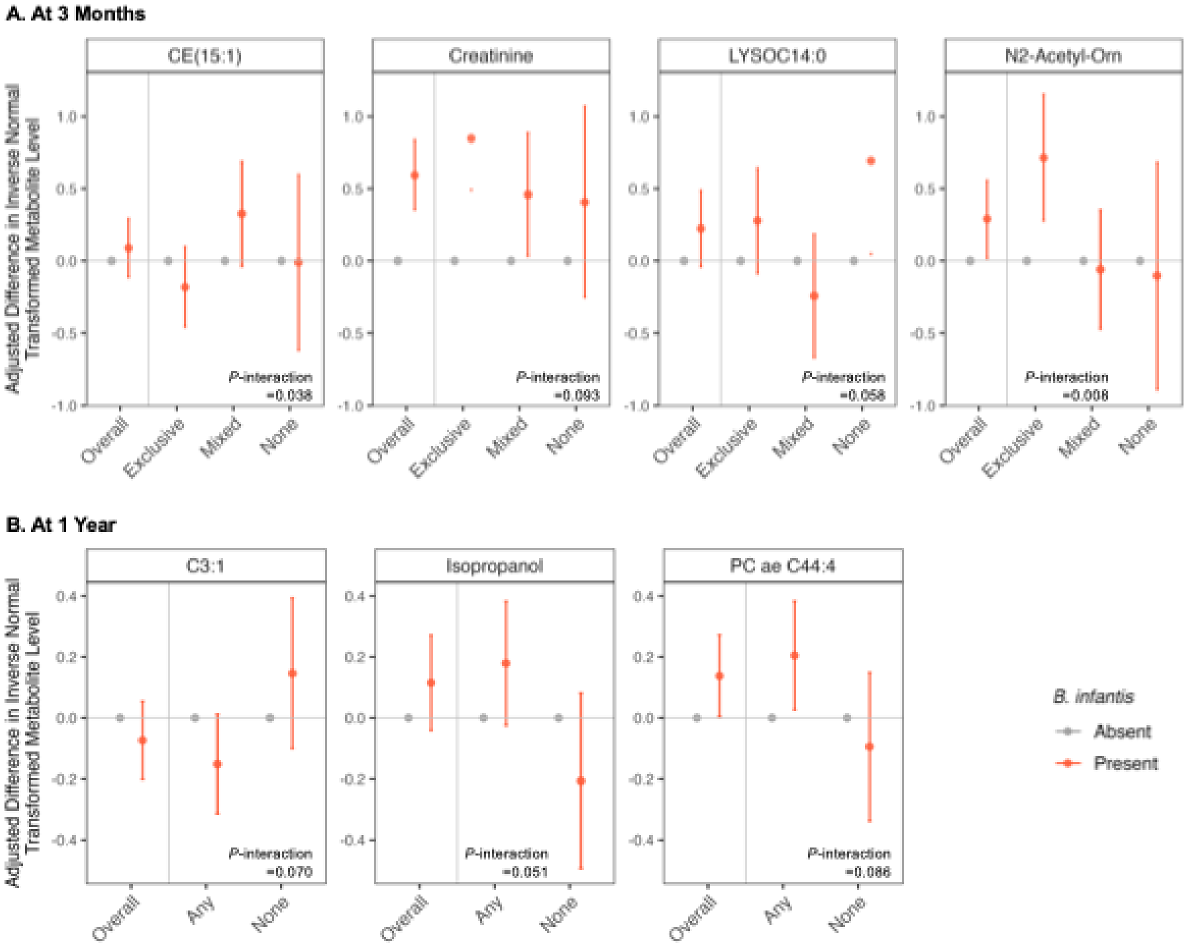

Estimates are based on mixed effect linear regressions with metabolite levels transformed by rank-based inverse normal transformation as the outcome. SBP-related metabolites are identified by elastic net regression in the overall sample or within each stratum of breastfeeding status. Significant interactions are shown here and Figure 2. Results for all metabolites are in eTables 6 and 7. Models adjusted for maternal age at delivery (continuous), pre-pregnancy body mass index (continuous), educational achievement (high school or below, college and university level, graduate degree), hypertension (chronic, during pregnancy, no), antibiotic use (prenatal, intrapartum, no), delivery mode (vaginal, C-section with labor, C-section without labor), child sex (boy, girl), birth weight (continuous), exact age in months (continuous) at fecal sample collection, time between sample collection and freezing (hours), with study site (Edmonton, Toronto, Vancouver, Winnipeg) as a random intercept.

eFigure 4. Correlation matrix between microbes (other than *Bifidobacterium*) and metabolites associated with SBP.

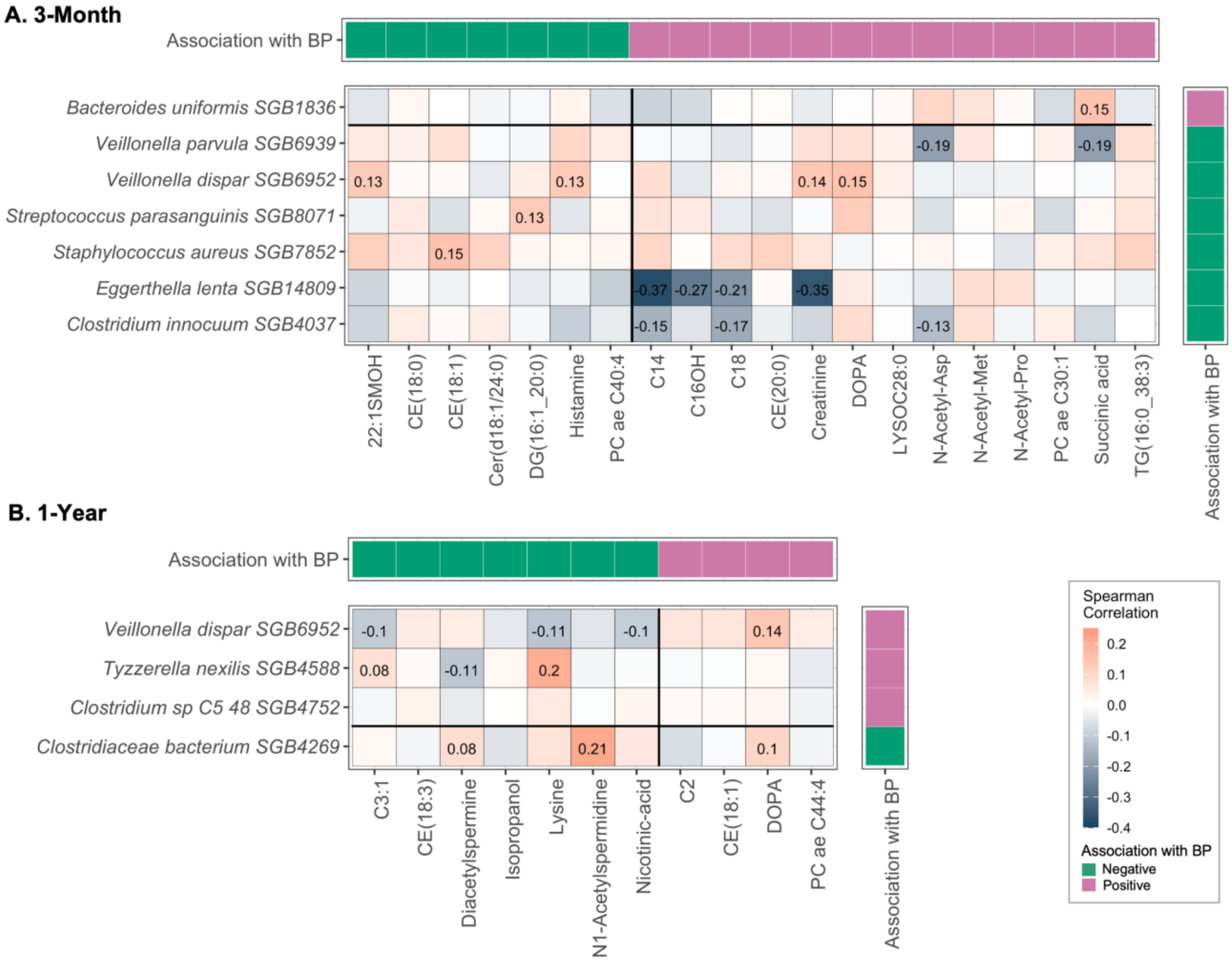

Correlation heatmaps between SBP-associated metabolites and relative abundances of SBP-associated microbes at 3 months (B1) and 1 year (B2) are based on the Spearman correlation coefficient. Cells marked with numbers indicate statistically significant correlations after controlling for multiple comparisons using the Benjamini-Hochberg method (*FDR-P*<0.05).

eFigure 5. Associations of Bray-Curtis distances at 3 month (A) and 1 year (B) with SBP percentiles at 3 and 5 years, overall and stratified by human milk feeding status.

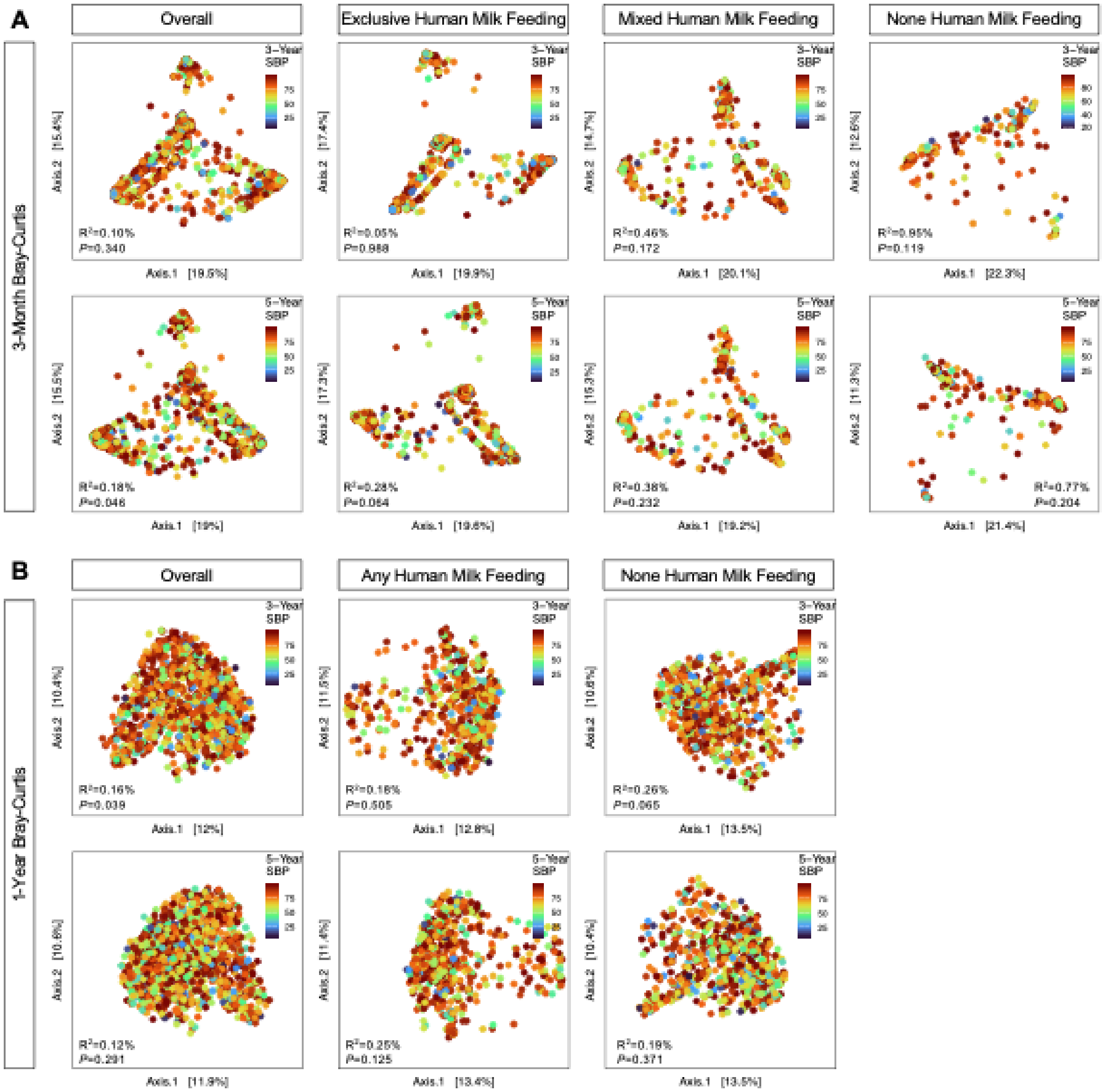

Percentage of variation (R^2) in Bray-Curtis distances that are explained by systolic blood pressure (SBP) percentiles are estimated by permutational multivariable analysis of variance (PERMANOVA), adjusting for maternal age at delivery (continuous), pre-pregnancy body mass index (continuous), educational achievement (high school or below, college and university level, graduate degree), hypertension (chronic, during pregnancy, no), antibiotic use (prenatal, intrapartum, no), delivery mode (vaginal, C-section with labor, C-section without labor), child sex (boy, girl), birth weight (continuous), and exact age in months (continuous) at fecal sample collection, with study site (Edmonton, Toronto, Vancouver, Winnipeg) as a random intercept.

eTable 1. Prevalence and relative abundance of *Bifidobacterium* Species at 3 months and 1 year.

Bifidobacterium Species	Timing of *Bifidobacterium* Measurement	Prevalence	Relative Abundance (%) Among Colonized Participants		
			25th Percentile	Median	75th Percentile
B. adolescentis SGB17244	3 Months	9.46%	1.01	11.79	26.93
	1 Year	24.65%	1.92	7.20	17.76
B. animalis SGB17278	3 Months	11.65%	0.17	1.30	4.56
	1 Year	42.06%	0.25	1.06	3.66
B. bifidum SGB17256	3 Months	24.22%	14.65	28.36	50.39
	1 Year	35.81%	4.80	10.80	20.15
B. breve SGB17247	3 Months	34.82%	7.31	49.36	77.24
	1 Year	49.93%	0.70	3.54	14.19
B. dentium SGB17234	3 Months	17.09%	0.20	2.37	20.00
	1 Year	23.95%	0.09	0.48	1.88
B. longum subsp.unclassified	3 Months	44.92%	0.94	3.37	7.32
	1 Year	64.61%	0.49	1.16	2.64
B. longum subsp.infantis	3 Months	13.42%	59.92	88.53	95.50
	1 Year	18.47%	2.58	15.34	42.97
B. longum subsp.longum	3 Months	62.85%	1.35	13.11	42.90
	1 Year	81.25%	3.02	7.79	16.80
B. pseudocatenulatum	3 Months	14.69%	4.35	22.24	46.55
	1 Year	29.14%	4.58	12.14	21.79

Prevalence is defined as the percentage of participants in whom the species is detected. Only Bifidobacterium species with ≥10% prevalence in the overall sample are examined in this study. Relative abundance summary statistics are calculated among participants in whom the species is present.

eTable 2. Characteristics of study population from the CHILD cohort study by human milk feeding status at 1 year.

	No (%)	
	Human milk feeding status at 1 year	
	Any (n=611)	None (n=680)
Maternal Characteristics		
White	452 (74.0)	508 (74.7)
Asian	89 (14.6)	90 (13.2)
Other	70 (11.5)	82 (12.1)
Highest education		
High school or below	21 (3.4)	65 (9.6)
College and university level	429 (70.2)	524 (77.1)
Graduate	161 (26.4)	91 (13.4)
Pre-pregnancy BMI, kg/m^2, mean (SD)	23.5 (4.0)	25.3 (5.8)
Age at delivery, years, mean (SD)	34.1 (4.2)	32.6 (4.6)
Hypertension		
Chronic	20 (3.3)	45 (6.6)
During pregnancy	17 (2.8)	26 (3.8)
No	574 (93.9)	609 (89.6)
Antibiotic use		
Prenatal by 18 weeks of gestation	18 (2.9)	23 (3.4)
Intrapartum	283 (46.3)	295 (43.4)
No	310 (50.7)	362 (53.2)
Child's Characteristics		
Boy	313 (51.2)	381 (56.0)
Girl	298 (48.8)	299 (44.0)
Delivery mode		
Vaginal	464 (75.9)	493 (72.5)
C-section with labor	83 (13.6)	93 (13.7)
C-section without labor	64 (10.5)	94 (13.8)
Birth weight, kg, mean (SD)	3.5 (0.5)	3.5 (0.5)
Gestational age, weeks, mean (SD)	39.7 (1.4)	39.5 (1.3)
Anthropometry and BP measures		
At 3 years of age		
BMI, kg/m^2, mean (SD)	16.1 (1.2)	16.4 (1.4)
SBP percentile, mean (SD)	74.9 (21.4)	74.6 (21.1)
DBP percentile, mean (SD)	80.8 (14.8)	80.6 (14.7)
At 5 years of age		
BMI, kg/m^2, mean (SD)	15.5 (1.3)	16.0 (1.6)
SBP percentile, mean (SD)	74.5 (20.8)	72.4 (23.1)
DBP percentile, mean (SD)	65.0 (18.4)	65.7 (19.7)

Abbreviations: BMI, body mass index; SBP, systolic blood pressure; DBP, diastolic blood pressure; SD, standard deviation.

eTable 3. Associations between human milk feeding status in infancy and SBP percentile in childhood, overall and stratified by presence/absence of *Bifidobacterium* species.

Timing of Feeding and *Bifidobacterium* Measurement	Overall and Stratification by Presence/Absence of *Bifidobacterium*		Human Milk Feeding Status	Adjusted Difference in SBP Percentile β (95% CI)	*P*	*P for interaction* between Feeding and *Bifidobacterium*
3 Months	Overall		None	Reference		
			Mixed	-0.26 (-3.44, 2.92)	0.873	
			Exclusive	-0.80 (-3.74, 2.14)	0.593	
3 Months	*B. adolescentis SGB17244*	Present	None	Reference		0.100
			Mixed	-9.88 (-20.29, 0.54)	0.063	
			Exclusive	-11.39 (-20.24, -2.55)	0.012	
		Absent	None	Reference		
			Mixed	0.94 (-2.49, 4.38)	0.590	
			Exclusive	0.64 (-2.55, 3.83)	0.694	
3 Months	*B. animalis SGB17278*	Present	None	Reference		0.786
			Mixed	-2.18 (-9.28, 4.91)	0.544	
			Exclusive	-5.16 (-13.83, 3.51)	0.241	
		Absent	None	Reference		
			Mixed	-0.10 (-3.81, 3.60)	0.958	
			Exclusive	-0.42 (-3.83, 3.00)	0.811	
3 Months	*B. bifidum SGB17256*	Present	None	Reference		0.032
			Mixed	-6.81 (-12.84, -0.77)	0.027	
			Exclusive	-7.99 (-13.67, -2.31)	0.006	
		Absent	None	Reference		
			Mixed	1.53 (-2.22, 5.28)	0.423	
			Exclusive	1.52 (-1.92, 4.96)	0.385	
3 Months	*B. breve SGB17247*	Present	None	Reference		0.332
			Mixed	-2.12 (-7.49, 3.25)	0.438	
			Exclusive	-3.66 (-8.75, 1.42)	0.157	
		Absent	None	Reference		
			Mixed	0.42 (-3.59, 4.43)	0.838	
			Exclusive	0.48 (-3.18, 4.15)	0.795	
3 Months	*B. dentium SGB17234*	Present	None	Reference		0.224
			Mixed	-3.52 (-10.38, 3.34)	0.313	
			Exclusive	-1.86 (-8.37, 4.64)	0.573	
		Absent	None	Reference		
			Mixed	1.02 (-2.58, 4.62)	0.579	
			Exclusive	-0.07 (-3.38, 3.25)	0.969	
3 Months	*B. longum subsp.infantis*	Present	None	Reference		0.107
			Mixed	-14.81 (-27.05, -2.56)	0.018	
			Exclusive	-17.16 (-29.48, -4.83)	0.007	
		Absent	None	Reference		
			Mixed	0.42 (-2.93, 3.77)	0.806	
			Exclusive	0.02 (-3.06, 3.10)	0.988	
3 Months	*B. longum subsp.longum*	Present	None	Reference		0.167
			Mixed	-1.56 (-5.49, 2.38)	0.438	
			Exclusive	-2.82 (-6.51, 0.86)	0.133	
		Absent	None	Reference		
			Mixed	2.29 (-3.25, 7.84)	0.417	
			Exclusive	3.04 (-2.03, 8.12)	0.239	
3 Months	*B. longum subsp.unclassified*	Present	None	Reference		0.306
			Mixed	-2.62 (-7.09, 1.85)	0.250	
			Exclusive	-3.05 (-7.26, 1.16)	0.155	
		Absent	None	Reference		
			Mixed	2.29 (-2.40, 6.97)	0.338	
			Exclusive	1.40 (-2.93, 5.74)	0.526	

Timing of Feeding and *Bifidobacterium* Measurement	Overall and Stratification by Presence/Absence of *Bifidobacterium*		Human Milk Feeding Status	Adjusted Difference in SBP Percentile β (95% CI)	*P*	*P for interaction* between Feeding and *Bifidobacterium*
3 Months	*B. pseudocatenulatum SGB17237*	Present	None	Reference		0.161
			Mixed	-5.02 (-14.11, 4.06)	0.276	
			Exclusive	-6.81 (-15.31, 1.68)	0.115	
		Absent	None	Reference		
			Mixed	0.59 (-2.82, 4.00)	0.734	
			Exclusive	0.12 (-3.03, 3.27)	0.940	
1 Year	Overall		None	Reference		
			Any	0.34 (-1.59, 2.27)	0.728	
1 Year	*B. adolescentis SGB17244*	Present	None	Reference		0.128
			Any	-2.88 (-6.78, 1.01)	0.146	
		Absent	None	Reference		
			Any	1.29 (-0.95, 3.53)	0.260	
1 Year	*B. animalis SGB17278*	Present	None	Reference		0.300
			Any	2.31 (-0.62, 5.25)	0.122	
		Absent	None	Reference		
			Any	-1.10 (-3.71, 1.50)	0.406	
1 Year	*B. bifidum SGB17256*	Present	None	Reference		0.856
			Any	-0.31 (-3.52, 2.91)	0.850	
		Absent	None	Reference		
			Any	0.75 (-1.72, 3.21)	0.552	
1 Year	*B. breve SGB17247*	Present	None	Reference		0.907
			Any	0.22 (-2.52, 2.96)	0.875	
		Absent	None	Reference		
			Any	0.16 (-2.60, 2.91)	0.912	
1 Year	*B. dentium SGB17234*	Present	None	Reference		0.499
			Any	0.21 (-3.87, 4.30)	0.918	
		Absent	None	Reference		
			Any	0.23 (-2.04, 2.50)	0.844	
1 Year	*B. longum subsp.infantis*	Present	None	Reference		0.385
			Any	-3.04 (-8.24, 2.16)	0.250	
		Absent	None	Reference		
			Any	0.34 (-1.84, 2.52)	0.760	
1 Year	*B. longum subsp.longum*	Present	None	Reference		0.926
			Any	0.64 (-1.54, 2.81)	0.564	
		Absent	None	Reference		
			Any	-0.48 (-4.92, 3.96)	0.832	
1 Year	*B. longum subsp.unclassified*	Present	None	Reference		0.334
			Any	-0.23 (-2.72, 2.26)	0.856	
		Absent	None	Reference		
			Any	1.03 (-2.14, 4.19)	0.524	
1 Year	*B. pseudocatenulatum SGB17237*	Present	None	Reference		0.128
			Any	2.86 (-0.81, 6.53)	0.126	
		Absent	None	Reference		
			Any	-0.68 (-2.99, 1.63)	0.563	

Estimates are from mixed-effect linear regression with systolic blood pressure (SBP) percentiles measured at 3 and 5 years modeled as a repeated outcome. Models adjusted for maternal age at delivery (continuous), pre-pregnancy body mass index (continuous), educational achievement (high school or below, college and university level, graduate degree), hypertension (chronic, during pregnancy, no), antibiotic use (prenatal, intrapartum, no), delivery mode (vaginal, C-section with labor, C-section without labor), child sex (boy, girl), birth weight (continuous), and exact age in months (continuous) at fecal sample collection, with study site (Edmonton, Toronto, Vancouver, Winnipeg) as a random intercept. Only *Bifidobacterium* species with ≥10% prevalence in the overall sample are examined. Results for human milk feeding status measured at 3 months stratified by presence/absence of *B. adolescentis*, *B. bifidum*, and *B. infantis* are visualized in Figure 1. Abbreviation: CI, confidence interval.

eTable 4. Associations between the presence/absence of *Bifidobacterium* species in infancy with SBP percentile in childhood, overall and stratified by human milk feeding status.

Timing of *Bifidobacterium* and Feeding Measurement	Overall and Stratification by Human Feeding Status	*Bifidobacterium* Species (Presence/Absence)		Adjusted Difference in SBP Percentile β (95% CI)	*P*	*P for interaction* between Feeding and *Bifidobacterium*
3 Months	Overall	*B. adolescentis SGB17244*	Absent	Reference		0.100
			Present	-0.21 (-3.35, 2.94)	0.898	
	Exclusive		Absent	Reference		
			Present	-3.12 (-7.57, 1.33)	0.170	
	Mixed		Absent	Reference		
			Present	-0.95 (-7.53, 5.63)	0.777	
	None		Absent	Reference		
			Present	6.19 (-0.54, 12.93)	0.071	
3 Months	Overall	*B. animalis SGB17278*	Absent	Reference		0.786
			Present	-0.08 (-2.92, 2.76)	0.955	
	Exclusive		Absent	Reference		
			Present	-1.68 (-7.74, 4.37)	0.585	
	Mixed		Absent	Reference		
			Present	-0.21 (-4.76, 4.33)	0.927	
	None		Absent	Reference		
			Present	-1.33 (-7.07, 4.41)	0.648	
3 Months	Overall	*B. bifidum SGB17256*	Absent	Reference		0.032
			Present	2.92 (0.79, 5.05)	0.007	
	Exclusive		Absent	Reference		
			Present	1.38 (-1.41, 4.18)	0.332	
	Mixed		Absent	Reference		
			Present	2.79 (-1.33, 6.92)	0.183	
	None		Absent	Reference		
			Present	9.92 (4.05, 15.79)	0.001	
3 Months	Overall	*B. breve SGB17247*	Absent	Reference		0.332
			Present	-0.09 (-1.98, 1.81)	0.926	
	Exclusive		Absent	Reference		
			Present	-1.20 (-3.66, 1.26)	0.340	
	Mixed		Absent	Reference		
			Present	1.11 (-2.59, 4.82)	0.555	
	None		Absent	Reference		
			Present	3.31 (-2.31, 8.93)	0.246	
3 Months	Overall	*B. dentium SGB17234*	Absent	Reference		0.224
			Present	-0.17 (-2.62, 2.29)	0.893	
	Exclusive		Absent	Reference		
			Present	1.20 (-2.13, 4.54)	0.480	
	Mixed		Absent	Reference		
			Present	-3.22 (-7.82, 1.37)	0.168	
	None		Absent	Reference		
			Present	3.84 (-2.88, 10.56)	0.260	
3 Months	Overall	*B. longum subsp.infantis*	Absent	Reference		0.107
			Present	1.97 (-0.69, 4.62)	0.147	
	Exclusive		Absent	Reference		
			Present	1.19 (-2.02, 4.39)	0.468	
	Mixed		Absent	Reference		
			Present	2.36 (-3.15, 7.86)	0.400	
	None		Absent	Reference		
			Present	16.93 (4.57, 29.28)	0.008	
3 Months	Overall	*B. longum subsp.longum*	Absent	Reference		0.167
			Present	-0.68 (-2.56, 1.20)	0.477	
	Exclusive		Absent	Reference		

Timing of *Bifidobacterium* and Feeding Measurement	Overall and Stratification by Human Feeding Status	*Bifidobacterium* Species (Presence/Absence)		Adjusted Difference in SBP Percentile β (95% CI)	*P*	*P for interaction* between Feeding and *Bifidobacterium*
			Present	-2.13 (-4.50, 0.25)	0.079	
	Mixed		Absent	Reference		
			Present	0.04 (-3.83, 3.91)	0.984	
	None		Absent	Reference		
			Present	3.64 (-1.92, 9.21)	0.198	
3 Months	Overall	*B. longum subsp.unclassified*	Absent	Reference		0.306
			Present	-1.18 (-3.02, 0.66)	0.208	
	Exclusive		Absent	Reference		
			Present	-2.21 (-4.60, 0.18)	0.069	
	Mixed		Absent	Reference		
			Present	-1.94 (-5.57, 1.68)	0.293	
	None		Absent	Reference		
			Present	2.88 (-2.61, 8.36)	0.302	
3 Months	Overall	*B. pseudocatenulatum SGB17237*	Absent	Reference		0.161
			Present	-1.15 (-3.69, 1.40)	0.378	
	Exclusive		Absent	Reference		
			Present	-2.08 (-5.39, 1.23)	0.218	
	Mixed		Absent	Reference		
			Present	-1.71 (-6.70, 3.28)	0.501	
	None		Absent	Reference		
			Present	4.51 (-2.74, 11.76)	0.221	
1 Year	Overall	*B. adolescentis SGB17244*	Absent	Reference		0.128
			Present	1.02 (-1.10, 3.15)	0.346	
	Any		Absent	Reference		
			Present	-0.99 (-4.04, 2.06)	0.523	
	None		Absent	Reference		
			Present	2.61 (-0.47, 5.69)	0.097	
1 Year	Overall	*B. animalis SGB17278*	Absent	Reference		0.300
			Present	-0.06 (-1.89, 1.78)	0.950	
	Any		Absent	Reference		
			Present	1.31 (-1.38, 3.99)	0.339	
	None		Absent	Reference		
			Present	-1.08 (-3.69, 1.54)	0.420	
1 Year	Overall	*B. bifidum SGB17256*	Absent	Reference		0.856
			Present	0.39 (-1.50, 2.27)	0.689	
	Any		Absent	Reference		
			Present	0.38 (-2.30, 3.07)	0.779	
	None		Absent	Reference		
			Present	0.28 (-2.52, 3.07)	0.846	
1 Year	Overall	*B. breve SGB17247*	Absent	Reference		0.907
			Present	1.37 (-0.44, 3.18)	0.138	
	Any		Absent	Reference		
			Present	1.51 (-1.13, 4.16)	0.262	
	None		Absent	Reference		
			Present	1.71 (-0.88, 4.30)	0.195	
1 Year	Overall	*B. dentium SGB17234*	Absent	Reference		0.499
			Present	0.76 (-1.39, 2.90)	0.489	
	Any		Absent	Reference		
			Present	0.93 (-1.89, 3.74)	0.518	
	None		Absent	Reference		
			Present	-0.55 (-4.15, 3.05)	0.765	
1 Year	Overall	*B. longum subsp.infantis*	Absent	Reference		0.385
			Present	2.33 (-0.02, 4.67)	0.052	

Timing of *Bifidobacterium* and Feeding Measurement	Overall and Stratification by Human Feeding Status	*Bifidobacterium* Species (Presence/Absence)		Adjusted Difference in SBP Percentile β (95% CI)	*P*	*P for interaction* between Feeding and *Bifidobacterium*
	Any		Absent	Reference		
			Present	1.58 (-1.33, 4.49)	0.287	
	None		Absent	Reference		
			Present	3.71 (-0.98, 8.39)	0.121	
1 Year	Overall	*B. longum subsp.longum*	Absent	Reference		0.926
			Present	-0.52 (-2.83, 1.79)	0.660	
	Any		Absent	Reference		
			Present	-0.83 (-4.37, 2.71)	0.646	
	None		Absent	Reference		
			Present	-0.75 (-3.94, 2.44)	0.645	
1 Year	Overall	*B. longum subsp.unclassified*	Absent	Reference		0.334
			Present	-1.08 (-2.99, 0.84)	0.269	
	Any		Absent	Reference		
			Present	-2.48 (-5.21, 0.24)	0.074	
	None		Absent	Reference		
			Present	-0.22 (-3.08, 2.64)	0.879	
1 Year	Overall	*B. pseudocatenulatum SGB17237*	Absent	Reference		0.128
			Present	-0.75 (-2.75, 1.24)	0.460	
	Any		Absent	Reference		
			Present	0.60 (-2.31, 3.52)	0.684	
	None		Absent	Reference		
			Present	-2.75 (-5.62, 0.12)	0.061	

Estimates are from mixed-effect linear regression with systolic blood pressure (SBP) percentile modeled as a repeated outcome. Only Bifidobacterium species with ≥10% prevalence in the overall sample were examined. Models adjusted for maternal age at delivery (continuous), pre-pregnancy body mass index (continuous), educational achievement (high school or below, college and university level, graduate degree), hypertension (chronic, during pregnancy, no), antibiotic use (prenatal, intrapartum, no), delivery mode (vaginal, C-section with labor, C-section without labor), child sex (boy, girl), birth weight (continuous), and exact age in months (continuous) at fecal sample collection, with study site (Edmonton, Toronto, Vancouver, Winnipeg) as a random intercept. Abbreviation: CI, confidence interval.

eTable 5. Associations between relative abundance of *Bifidobacterium* species in infancy with SBP percentile in childhood, overall and stratified by human milk feeding status.

Timing of *Bifidobacterium* and Feeding Measurement	Overall and Stratification by Human Feeding Status	*Bifidobacterium* Species (CLR Transformed Relative Abundance)	Adjusted Difference in SBP Percentile β (95% CI)	*P*	*P for interaction* between Feeding and *Bifidobacterium*
3 Months	Overall	*B. adolescentis SGB17244*	0.02 (-0.26, 0.29)	0.903	0.120
	Exclusive		-0.28 (-0.68, 0.13)	0.178	
	Mixed		0.02 (-0.57, 0.60)	0.958	
	None		0.46 (-0.08, 0.99)	0.095	
3 Months	Overall	*B. animalis SGB17278*	-0.04 (-0.32, 0.25)	0.807	0.955
	Exclusive		-0.07 (-0.71, 0.56)	0.822	
	Mixed		-0.12 (-0.57, 0.34)	0.619	
	None		-0.14 (-0.68, 0.41)	0.624	
3 Months	Overall	*B. bifidum SGB17256*	0.21 (0.04, 0.37)	0.015	0.016
	Exclusive		0.06 (-0.16, 0.28)	0.587	
	Mixed		0.23 (-0.08, 0.55)	0.141	
	None		0.79 (0.33, 1.25)	0.001	
3 Months	Overall	*B. breve SGB17247*	-0.02 (-0.16, 0.13)	0.827	0.432
	Exclusive		-0.08 (-0.27, 0.11)	0.414	
	Mixed		0.05 (-0.23, 0.34)	0.710	
	None		0.24 (-0.21, 0.69)	0.302	
3 Months	Overall	*B. dentium SGB17234*	-0.01 (-0.24, 0.22)	0.946	0.240
	Exclusive		0.10 (-0.20, 0.40)	0.530	
	Mixed		-0.29 (-0.74, 0.16)	0.202	
	None		0.40 (-0.26, 1.07)	0.233	
3 Months	Overall	*B. longum subsp.infantis*	0.15 (-0.06, 0.35)	0.154	0.063
	Exclusive		0.08 (-0.16, 0.32)	0.503	
	Mixed		0.24 (-0.20, 0.68)	0.283	
	None		1.84 (0.59, 3.09)	0.004	
3 Months	Overall	*B. longum subsp.longum*	-0.05 (-0.21, 0.10)	0.494	0.266
	Exclusive		-0.15 (-0.35, 0.05)	0.136	
	Mixed		-0.07 (-0.38, 0.24)	0.653	
	None		0.31 (-0.14, 0.75)	0.173	
3 Months	Overall	*B. longum subsp.unclassified*	-0.12 (-0.29, 0.05)	0.158	0.337
	Exclusive		-0.21 (-0.43, 0.02)	0.068	
	Mixed		-0.21 (-0.55, 0.13)	0.219	
	None		0.25 (-0.26, 0.77)	0.327	
3 Months	Overall	*B. pseudocatenulatum SGB17237*	-0.12 (-0.33, 0.09)	0.270	0.124
	Exclusive		-0.20 (-0.48, 0.07)	0.149	
	Mixed		-0.18 (-0.58, 0.23)	0.393	
	None		0.38 (-0.21, 0.96)	0.204	
1 Year	Overall	*B. adolescentis SGB17244*	0.13 (-0.06, 0.31)	0.192	0.117
	Any		-0.05 (-0.32, 0.22)	0.705	
	None		0.28 (0.00, 0.55)	0.050	
1 Year	Overall	*B. animalis SGB17278*	0.01 (-0.18, 0.19)	0.955	0.209
	Any		0.17 (-0.11, 0.46)	0.227	
	None		-0.12 (-0.38, 0.14)	0.378	
1 Year	Overall	*B. bifidum SGB17256*	0.03 (-0.13, 0.20)	0.682	0.911
	Any		0.01 (-0.21, 0.23)	0.933	
	None		0.05 (-0.20, 0.30)	0.691	
1 Year	Overall	*B. breve SGB17247*	0.15 (-0.02, 0.31)	0.082	0.498
	Any		0.12 (-0.12, 0.35)	0.326	
	None		0.23 (-0.02, 0.48)	0.066	
1 Year	Overall	*B. dentium SGB17234*	0.05 (-0.19, 0.29)	0.658	0.737
	Any		0.05 (-0.27, 0.36)	0.775	
	None		-0.03 (-0.43, 0.37)	0.885	

Timing of *Bifidobacterium* and Feeding Measurement	Overall and Stratification by Human Feeding Status	*Bifidobacterium* Species (CLR Transformed Relative Abundance)	Adjusted Difference in SBP Percentile β (95% CI)	*P*	*P for interaction* between Feeding and *Bifidobacterium*
1 Year	Overall	*B. longum subsp.infantis*	0.21 (0.01, 0.41)	0.043	0.525
	Any		0.17 (-0.07, 0.41)	0.156	
	None		0.30 (-0.15, 0.75)	0.192	
1 Year	Overall	*B. longum subsp.longum*	-0.06 (-0.25, 0.14)	0.551	0.789
	Any		-0.13 (-0.43, 0.17)	0.389	
	None		-0.05 (-0.32, 0.22)	0.727	
1 Year	Overall	*B. longum subsp.unclassified*	-0.17 (-0.36, 0.03)	0.088	0.274
	Any		-0.32 (-0.60, -0.05)	0.023	
	None		-0.07 (-0.36, 0.22)	0.638	
1 Year	Overall	*B. pseudocatenulatum SGB17237*	-0.05 (-0.22, 0.12)	0.554	0.148
	Any		0.06 (-0.19, 0.30)	0.650	
	None		-0.21 (-0.46, 0.03)	0.089	

Estimates are from mixed-effect linear regression with systolic blood pressure (SBP) percentile measured at 3 and 5 years modeled as a repeated outcome. Only *Bifidobacterium* species with ≥10% prevalence in the overall sample were examined. Models adjusted for maternal age at delivery (continuous), pre-pregnancy body mass index (continuous), educational achievement (high school or below, college and university level, graduate degree), hypertension (chronic, during pregnancy, no), antibiotic use (prenatal, intrapartum, no), delivery mode (vaginal, C-section with labor, C-section without labor), child sex (boy, girl), birth weight (continuous), and exact age in months (continuous) at fecal sample collection, with study site (Edmonton, Toronto, Vancouver, Winnipeg) as a random intercept. Abbreviations: CLR, centered log ratio; CI, confidence interval.

eTable 6. Fecal metabolites in infancy that are associated with SBP percentile in childhood, overall and within each stratum of human milk feeding status.

Timing of Metabolites and Feeding Measurement	Overall and Stratification by Human Feeding Status	Metabolite	Metabolite Class	Outcome	Elastic Net Coefficients
3 Months	Overall	N-Acetyl-Pro	Acetylated amino acid	SBP at 3 years	0.61
	Exclusive	C12DC	Acylcarnitine		0.83
		N-Acetyl-Pro	Acetylated amino acid		0.76
		LYSOC16:0	Lipid		-0.48
		LYSOC14:0	Lipid		-0.86
		PC38:0AA	Lipid		-0.91
	Mixed	Methylmalonic acid	Dicarboxylic acid		1.4
		PC aa C42:2	Lipid		1.08
		Hex2Cer(d18:1/14:0)	Lipid		0.98
3 Months	Overall	CE(20:0)	Lipid	SBP at 5 years	1.6
		Succinic acid	Dicarboxylic acid		1.21
		Creatinine	Heterocyclic compound		1.12
		PC ae C30:1	Lipid		0.98
		N-Acetyl-Asp	Acetylated amino acid		0.9
		LYSOC28:0	Lipid		0.9
		N-Acetyl-Met	Acetylated amino acid		0.75
		TG(16:0_38:3)	Lipid		0.65
		C18	Acylcarnitine		0.58
		C16OH	Acylcarnitine		0.46
		DOPA	Amino acid derivative		0.43
		C14	Acylcarnitine		0.42
		22:1SMOH	Lipid		-0.33
		Histamine	Biogenic amine		-0.39
		PC ae C40:4	Lipid		-0.4
		Cer(d18:1/24:0)	Lipid		-0.89
		DG(16:1_20:0)	Lipid		-0.98
		CE(18:1)	Lipid		-1.07
		CE(18:0)	Lipid		-1.16
	Exclusive	C14	Acylcarnitine		1.73
		HexCer(d18:1/24:0)	Lipid		-1.53
	None	Arabinose	Monosaccharide		1.53
		Oxalacetic acid	Dicarboxylic acid		1.4
		N-Acetyl-Met	Acetylated amino acid		1.38
		Guanidinopropionic acid	Guanidine derivative		1.33
		CE(15:1)	Lipid		1.32
		trans-4-Hydroxyproline	Hydroxy amino acid		0.92
		C12DC	Acylcarnitine		0.88
		N-Acetyl-His	Acetylated amino acid		0.87
		C0	Acylcarnitine		0.86
		3-Hydroxyisobutyric acid	Hydroxy acid		0.79
		2-Hydroxy-3-methylvaleric acid	Hydroxy acid		0.76
		N2-Acetyl-Orn	Acetylated amino acid		0.74
		CE(20:0)	Lipid		0.72
		C4OH	Acylcarnitine		0.72

Timing of Metabolites and Feeding Measurement	Overall and Stratification by Human Feeding Status	Metabolite	Metabolite Class	Outcome	Elastic Net Coefficients
		4-Hydroxybenzoic acid	Phenolic acid		0.68
		4-Hydroxyphenylacetic acid	Phenolic acid		0.68
		TG(16:0_37:3)	Lipid		0.63
		5-Hydroxylysine	Hydroxy amino acid		0.56
		C5	Acylcarnitine		0.51
		C5OH	Acylcarnitine		0.36
		Succinic acid	Dicarboxylic acid		0.3
		N1-Acetyl-Lys	Acetylated amino acid		0.22
		16:1SMOH	Lipid		-0.67
		PC ae C36:2	Lipid		-0.75
		Cer(d18:1/16:0)	Lipid		-0.78
		22:1SMOH	Lipid		-0.79
		22:2SMOH	Lipid		-0.92
		Agmatine	Biogenic amine		-1.04
1 Year	Overall	PC ae C44:4	Lipid	SBP at 5 years	0.59
		CE(18:1)	Lipid		0.45
		DOPA	Amino acid derivative		0.25
		C2	Acylcarnitine		0.21
		Isopropanol	Alcohol		-0.19
		N1-Acetylspermidine	Acetylated polyamine		-0.22
		Lysine	Amino acid		-0.24
		Diacetylspermine	Acetylated polyamine		-0.28
		C3:1	Acylcarnitine		-0.31
		Nicotinic-acid	Vitamin		-0.35
		CE(18:3)	Lipid		-0.38
	None	2-Hydroxyphenylacetic acid	Phenolic acid		-0.22
		Glutamic acid	Amino acid		-0.39
		Nicotinic-acid	Vitamin		-0.41
		Hex3Cer(d18:1/18:0)	Lipid		-0.45
		C3:1	Acylcarnitine		-0.76
		Argininic acid	Amino acid derivative		-0.86

Significant metabolites are identified by elastic net regression conducted overall and within each category of human milk feeding status assessed at the time of metabolite measurement. Models accounted for maternal age at delivery (continuous), pre-pregnancy body mass index (continuous), educational achievement (high school or below, college and university level, graduate degree), hypertension (chronic, during pregnancy, no), antibiotic use (prenatal, intrapartum, no), delivery mode (vaginal, C-section with labor, C-section without labor), child sex (boy, girl), birth weight (continuous), exact age in months (continuous) at fecal sample collection, time between sample collection and freezing (hours), and study site (Edmonton, Toronto, Vancouver, Winnipeg).

eTable 7. Associations between human milk feeding status and SBP-related fecal metabolites at 3 months and 1 year, overall and by presence/absence of *B. infantis*.

Timing of Feeding and *B. infantis* Measurement	Overall and Stratification by Presence/Absence of *B. infantis*	Human Milk Feeding Status	Metabolite	Adjusted Difference in Inverse Normal Transformed Metabolite Level β (95% CI)	*P*	*P for interaction* between Feeding and *B. infantis*
3 Months	Overall	None	16:1SMOH	Reference		0.528
		Mixed		0.33 (0.10, 0.56)	0.005	
		Exclusive		0.63 (0.40, 0.86)	0.000	
	B. infantis Present	None		Reference		
		Mixed		-0.00 (-0.96, 0.95)	0.998	
		Exclusive		0.54 (-0.41, 1.49)	0.260	
	B. infantis Absent	None		Reference		
		Mixed		0.37 (0.13, 0.62)	0.003	
		Exclusive		0.62 (0.38, 0.87)	0.000	
3 Months	Overall	None	2-Hydroxy-3-methylvaleric acid	Reference		0.619
		Mixed		0.35 (0.09, 0.60)	0.007	
		Exclusive		0.06 (-0.20, 0.31)	0.668	
	B. infantis Present	None		Reference		
		Mixed		-0.23 (-1.18, 0.72)	0.631	
		Exclusive		-0.61 (-1.55, 0.34)	0.202	
	B. infantis Absent	None		Reference		
		Mixed		0.37 (0.10, 0.64)	0.007	
		Exclusive		0.10 (-0.17, 0.37)	0.466	
3 Months	Overall	None	22:1SMOH	Reference		0.336
		Mixed		0.09 (-0.16, 0.33)	0.488	
		Exclusive		0.47 (0.23, 0.72)	0.000	
	B. infantis Present	None		Reference		
		Mixed		-0.31 (-1.34, 0.72)	0.544	
		Exclusive		0.49 (-0.53, 1.52)	0.335	
	B. infantis Absent	None		Reference		
		Mixed		0.14 (-0.12, 0.40)	0.290	
		Exclusive		0.47 (0.21, 0.73)	0.000	
3 Months	Overall	None	22:2SMOH	Reference		0.927
		Mixed		0.27 (0.05, 0.50)	0.018	
		Exclusive		0.51 (0.29, 0.74)	0.000	
	B. infantis Present	None		Reference		
		Mixed		0.54 (-0.36, 1.44)	0.234	
		Exclusive		0.71 (-0.18, 1.61)	0.114	
	B. infantis Absent	None		Reference		
		Mixed		0.26 (0.02, 0.50)	0.036	
		Exclusive		0.50 (0.26, 0.74)	0.000	
3 Months	Overall	None	3-Hydroxyisobutyric acid	Reference		0.853
		Mixed		-0.20 (-0.43, 0.03)	0.095	
		Exclusive		-0.68 (-0.92, -0.45)	0.000	
	B. infantis Present	None		Reference		
		Mixed		-0.55 (-1.47, 0.37)	0.236	
		Exclusive		-1.06 (-1.97, -0.14)	0.025	
	B. infantis Absent	None		Reference		
		Mixed		-0.16 (-0.42, 0.09)	0.201	
		Exclusive		-0.63 (-0.88, -0.37)	0.000	
3 Months	Overall	None	4-Hydroxybenzoic acid	Reference		0.870
		Mixed		0.00 (-0.23, 0.24)	0.975	
		Exclusive		-0.31 (-0.54, -0.08)	0.010	
	B. infantis Present	None		Reference		
		Mixed		-0.33 (-1.18, 0.53)	0.442	

Timing of Feeding and *B. infantis* Measurement	Overall and Stratification by Presence/Absence of *B. infantis*	Human Milk Feeding Status	Metabolite	Adjusted Difference in Inverse Normal Transformed Metabolite Level β (95% CI)	*P*	*P for interaction between Feeding and B. infantis*
		Exclusive		-0.57 (-1.42, 0.28)	0.185	
	B. infantis Absent	None		Reference		
		Mixed		0.04 (-0.21, 0.29)	0.749	
		Exclusive		-0.26 (-0.51, -0.01)	0.041	
3 Months	Overall	None	4-Hydroxyphenylacetic acid	Reference		0.307
		Mixed		-0.17 (-0.40, 0.07)	0.166	
		Exclusive		-0.66 (-0.90, -0.42)	0.000	
	B. infantis Present	None		Reference		
		Mixed		0.09 (-0.85, 1.03)	0.852	
		Exclusive		-0.74 (-1.68, 0.20)	0.118	
	B. infantis Absent	None		Reference		
		Mixed		-0.21 (-0.46, 0.04)	0.094	
		Exclusive		-0.63 (-0.88, -0.38)	0.000	
3 Months	Overall	None	5-Hydroxylysine	Reference		0.851
		Mixed		-0.50 (-0.73, -0.27)	0.000	
		Exclusive		-0.98 (-1.21, -0.75)	0.000	
	B. infantis Present	None		Reference		
		Mixed		-0.46 (-1.39, 0.46)	0.320	
		Exclusive		-1.07 (-1.99, -0.14)	0.025	
	B. infantis Absent	None		Reference		
		Mixed		-0.52 (-0.76, -0.27)	0.000	
		Exclusive		-0.97 (-1.22, -0.72)	0.000	
3 Months	Overall	None	Agmatine	Reference		0.682
		Mixed		0.31 (0.07, 0.55)	0.011	
		Exclusive		0.60 (0.35, 0.84)	0.000	
	B. infantis Present	None		Reference		
		Mixed		0.14 (-0.84, 1.11)	0.777	
		Exclusive		0.61 (-0.36, 1.58)	0.213	
	B. infantis Absent	None		Reference		
		Mixed		0.35 (0.09, 0.61)	0.008	
		Exclusive		0.63 (0.36, 0.89)	0.000	
3 Months	Overall	None	Arabinose	Reference		0.103
		Mixed		0.21 (-0.06, 0.47)	0.121	
		Exclusive		0.32 (0.03, 0.61)	0.033	
	B. infantis Present	None		Reference		
		Mixed		-0.41 (-1.40, 0.58)	0.404	
		Exclusive		0.66 (-0.37, 1.70)	0.201	
	B. infantis Absent	None		Reference		
		Mixed		0.28 (-0.01, 0.56)	0.059	
		Exclusive		0.27 (-0.05, 0.59)	0.102	
3 Months	Overall	None	C0	Reference		0.341
		Mixed		0.04 (-0.21, 0.28)	0.779	
		Exclusive		0.21 (-0.04, 0.45)	0.104	
	B. infantis Present	None		Reference		
		Mixed		-0.45 (-1.44, 0.54)	0.365	
		Exclusive		-0.03 (-1.02, 0.96)	0.950	
	B. infantis Absent	None		Reference		
		Mixed		0.08 (-0.17, 0.34)	0.523	
		Exclusive		0.21 (-0.05, 0.47)	0.111	
3 Months	Overall	None	C12DC	Reference		0.830
		Mixed		-0.36 (-0.60, -0.11)	0.004	
		Exclusive		-0.45 (-0.70, -0.21)	0.000	
	B. infantis Present	None		Reference		

Timing of Feeding and *B. infantis* Measurement	Overall and Stratification by Presence/Absence of *B. infantis*	Human Milk Feeding Status	Metabolite	Adjusted Difference in Inverse Normal Transformed Metabolite Level β (95% CI)	*P*	*P for interaction* between Feeding and *B. infantis*
		Mixed		-0.35 (-1.17, 0.46)	0.388	
		Exclusive		-0.15 (-0.96, 0.67)	0.718	
	B. infantis Absent	None		Reference		
		Mixed		-0.35 (-0.62, -0.09)	0.008	
		Exclusive		-0.46 (-0.72, -0.19)	0.001	
3 Months	Overall	None	C14	Reference		0.437
		Mixed		0.03 (-0.20, 0.26)	0.812	
		Exclusive		0.50 (0.27, 0.74)	0.000	
	B. infantis Present	None		Reference		
		Mixed		-0.28 (-1.07, 0.52)	0.485	
		Exclusive		0.33 (-0.46, 1.12)	0.404	
	B. infantis Absent	None		Reference		
		Mixed		0.09 (-0.16, 0.33)	0.498	
		Exclusive		0.56 (0.31, 0.81)	0.000	
3 Months	Overall	None	C16OH	Reference		0.474
		Mixed		-0.04 (-0.24, 0.17)	0.721	
		Exclusive		0.06 (-0.15, 0.27)	0.559	
	B. infantis Present	None		Reference		
		Mixed		-0.67 (-1.49, 0.16)	0.109	
		Exclusive		-0.19 (-1.01, 0.63)	0.640	
	B. infantis Absent	None		Reference		
		Mixed		-0.00 (-0.22, 0.21)	0.966	
		Exclusive		0.07 (-0.15, 0.29)	0.513	
3 Months	Overall	None	C18	Reference		0.329
		Mixed		0.16 (-0.08, 0.39)	0.192	
		Exclusive		0.66 (0.42, 0.89)	0.000	
	B. infantis Present	None		Reference		
		Mixed		-0.36 (-1.14, 0.42)	0.353	
		Exclusive		0.34 (-0.44, 1.11)	0.384	
	B. infantis Absent	None		Reference		
		Mixed		0.23 (-0.03, 0.49)	0.080	
		Exclusive		0.70 (0.44, 0.96)	0.000	
3 Months	Overall	None	C4OH	Reference		0.101
		Mixed		-0.16 (-0.39, 0.08)	0.203	
		Exclusive		-0.58 (-0.82, -0.33)	0.000	
	B. infantis Present	None		Reference		
		Mixed		-0.43 (-1.44, 0.57)	0.389	
		Exclusive		-0.98 (-1.98, 0.01)	0.053	
	B. infantis Absent	None		Reference		
		Mixed		-0.11 (-0.36, 0.14)	0.401	
		Exclusive		-0.45 (-0.71, -0.20)	0.001	
3 Months	Overall	None	C5	Reference		0.778
		Mixed		0.03 (-0.22, 0.27)	0.833	
		Exclusive		-0.11 (-0.36, 0.14)	0.374	
	B. infantis Present	None		Reference		
		Mixed		-0.37 (-1.50, 0.75)	0.506	
		Exclusive		-0.36 (-1.48, 0.76)	0.523	
	B. infantis Absent	None		Reference		
		Mixed		0.07 (-0.19, 0.33)	0.584	
		Exclusive		-0.07 (-0.33, 0.19)	0.580	
3 Months	Overall	None	C5OH	Reference		0.636
		Mixed		0.00 (-0.25, 0.25)	0.983	
		Exclusive		-0.15 (-0.40, 0.10)	0.236	

Timing of Feeding and *B. infantis* Measurement	Overall and Stratification by Presence/Absence of *B. infantis*	Human Milk Feeding Status	Metabolite	Adjusted Difference in Inverse Normal Transformed Metabolite Level β (95% CI)	*P*	*P for interaction* between Feeding and *B. infantis*
	B. infantis Present	None		Reference		
		Mixed		-0.30 (-1.50, 0.90)	0.618	
		Exclusive		-0.46 (-1.66, 0.73)	0.437	
	B. infantis Absent	None		Reference		
		Mixed		0.02 (-0.23, 0.28)	0.859	
		Exclusive		-0.11 (-0.37, 0.15)	0.393	
3 Months	Overall	None	CE(15:1)	Reference		0.038
		Mixed		0.16 (-0.02, 0.35)	0.077	
		Exclusive		-0.02 (-0.20, 0.16)	0.827	
	B. infantis Present	None		Reference		
		Mixed		0.56 (0.01, 1.10)	0.046	
		Exclusive		0.02 (-0.52, 0.57)	0.932	
	B. infantis Absent	None		Reference		
		Mixed		0.06 (-0.14, 0.26)	0.557	
		Exclusive		-0.04 (-0.24, 0.16)	0.725	
3 Months	Overall	None	CE(18:0)	Reference		0.429
		Mixed		-0.02 (-0.24, 0.20)	0.853	
		Exclusive		-0.00 (-0.22, 0.22)	0.998	
	B. infantis Present	None		Reference		
		Mixed		0.66 (-0.06, 1.38)	0.073	
		Exclusive		0.86 (0.14, 1.57)	0.020	
	B. infantis Absent	None		Reference		
		Mixed		-0.09 (-0.32, 0.15)	0.459	
		Exclusive		-0.06 (-0.30, 0.18)	0.612	
3 Months	Overall	None	CE(18:1)	Reference		0.415
		Mixed		-0.05 (-0.26, 0.16)	0.639	
		Exclusive		-0.07 (-0.28, 0.14)	0.523	
	B. infantis Present	None		Reference		
		Mixed		0.74 (0.02, 1.46)	0.044	
		Exclusive		0.56 (-0.15, 1.28)	0.121	
	B. infantis Absent	None		Reference		
		Mixed		-0.13 (-0.36, 0.09)	0.248	
		Exclusive		-0.12 (-0.35, 0.11)	0.313	
3 Months	Overall	None	CE(20:0)	Reference		0.400
		Mixed		0.18 (-0.02, 0.37)	0.080	
		Exclusive		0.06 (-0.14, 0.26)	0.531	
	B. infantis Present	None		Reference		
		Mixed		0.55 (-0.08, 1.18)	0.087	
		Exclusive		0.26 (-0.37, 0.89)	0.418	
	B. infantis Absent	None		Reference		
		Mixed		0.12 (-0.09, 0.34)	0.251	
		Exclusive		0.06 (-0.16, 0.28)	0.586	
3 Months	Overall	None	Cer(d18:1/16:0)	Reference		0.515
		Mixed		0.05 (-0.20, 0.30)	0.705	
		Exclusive		0.03 (-0.22, 0.28)	0.804	
	B. infantis Present	None		Reference		
		Mixed		-0.54 (-1.44, 0.36)	0.230	
		Exclusive		-0.45 (-1.34, 0.44)	0.315	
	B. infantis Absent	None		Reference		
		Mixed		0.06 (-0.21, 0.33)	0.649	
		Exclusive		0.07 (-0.20, 0.34)	0.617	
3 Months	Overall	None	Cer(d18:1/24:0)	Reference		0.526
		Mixed		0.09 (-0.16, 0.33)	0.487	

Timing of Feeding and *B. infantis* Measurement	Overall and Stratification by Presence/Absence of *B. infantis*	Human Milk Feeding Status	Metabolite	Adjusted Difference in Inverse Normal Transformed Metabolite Level β (95% CI)	*P*	*P for interaction* between Feeding and *B. infantis*
		Exclusive		0.03 (-0.22, 0.28)	0.797	
	B. infantis Present	None		Reference		
		Mixed		-0.15 (-1.02, 0.73)	0.738	
		Exclusive		-0.20 (-1.07, 0.67)	0.642	
	B. infantis Absent	None		Reference		
		Mixed		0.14 (-0.12, 0.40)	0.294	
		Exclusive		0.11 (-0.16, 0.38)	0.422	
3 Months	Overall	None	Creatinine	Reference		0.093
		Mixed		0.03 (-0.20, 0.25)	0.806	
		Exclusive		0.30 (0.08, 0.53)	0.009	
	B. infantis Present	None		Reference		
		Mixed		0.16 (-0.77, 1.08)	0.734	
		Exclusive		1.00 (0.08, 1.92)	0.035	
	B. infantis Absent	None		Reference		
		Mixed		-0.03 (-0.26, 0.20)	0.807	
		Exclusive		0.16 (-0.07, 0.39)	0.172	
3 Months	Overall	None	DG(16:1_20:0)	Reference		0.569
		Mixed		0.14 (-0.08, 0.36)	0.209	
		Exclusive		0.02 (-0.20, 0.24)	0.852	
	B. infantis Present	None		Reference		
		Mixed		0.62 (-0.30, 1.53)	0.182	
		Exclusive		0.30 (-0.61, 1.22)	0.505	
	B. infantis Absent	None		Reference		
		Mixed		0.11 (-0.12, 0.35)	0.332	
		Exclusive		0.02 (-0.21, 0.26)	0.849	
3 Months	Overall	None	DOPA	Reference		0.268
		Mixed		-0.20 (-0.41, 0.02)	0.072	
		Exclusive		-0.33 (-0.55, -0.11)	0.003	
	B. infantis Present	None		Reference		
		Mixed		-0.51 (-1.36, 0.34)	0.235	
		Exclusive		-0.11 (-0.96, 0.74)	0.794	
	B. infantis Absent	None		Reference		
		Mixed		-0.15 (-0.38, 0.08)	0.196	
		Exclusive		-0.35 (-0.58, -0.12)	0.003	
3 Months	Overall	None	Guanidinopropionic acid	Reference		0.909
		Mixed		-0.05 (-0.29, 0.20)	0.709	
		Exclusive		0.27 (0.02, 0.51)	0.032	
	B. infantis Present	None		Reference		
		Mixed		-0.25 (-1.18, 0.67)	0.588	
		Exclusive		0.31 (-0.61, 1.23)	0.502	
	B. infantis Absent	None		Reference		
		Mixed		-0.05 (-0.31, 0.21)	0.716	
		Exclusive		0.24 (-0.02, 0.50)	0.069	
3 Months	Overall	None	Hex2Cer(d18:1/14:0)	Reference		0.279
		Mixed		0.45 (0.21, 0.69)	0.000	
		Exclusive		0.51 (0.27, 0.75)	0.000	
	B. infantis Present	None		Reference		
		Mixed		-0.39 (-1.37, 0.58)	0.422	
		Exclusive		-0.05 (-1.02, 0.92)	0.918	
	B. infantis Absent	None		Reference		
		Mixed		0.48 (0.23, 0.74)	0.000	
		Exclusive		0.49 (0.23, 0.75)	0.000	
3 Months	Overall	None	HexCer(d18:1/24:0)	Reference		0.276

Timing of Feeding and *B. infantis* Measurement	Overall and Stratification by Presence/Absence of *B. infantis*	Human Milk Feeding Status	Metabolite	Adjusted Difference in Inverse Normal Transformed Metabolite Level β (95% CI)	*P*	*P for interaction between Feeding and B. infantis*
		Mixed		0.57 (0.34, 0.81)	0.000	
		Exclusive		0.67 (0.43, 0.91)	0.000	
	B. infantis Present	None		Reference		
		Mixed		0.01 (-0.82, 0.84)	0.985	
		Exclusive		0.34 (-0.49, 1.17)	0.414	
	B. infantis Absent	None		Reference		
		Mixed		0.62 (0.37, 0.88)	0.000	
		Exclusive		0.73 (0.47, 0.99)	0.000	
3 Months	Overall	None	Histamine	Reference		0.990
		Mixed		0.02 (-0.22, 0.27)	0.848	
		Exclusive		0.32 (0.07, 0.57)	0.013	
	B. infantis Present	None		Reference		
		Mixed		-0.18 (-1.22, 0.86)	0.729	
		Exclusive		0.07 (-0.96, 1.11)	0.887	
	B. infantis Absent	None		Reference		
		Mixed		-0.02 (-0.28, 0.24)	0.865	
		Exclusive		0.29 (0.02, 0.55)	0.033	
3 Months	Overall	None	LYSOC14:0	Reference		0.058
		Mixed		0.42 (0.20, 0.64)	0.000	
		Exclusive		0.99 (0.77, 1.21)	0.000	
	B. infantis Present	None		Reference		
		Mixed		-0.13 (-0.95, 0.68)	0.740	
		Exclusive		0.87 (0.06, 1.68)	0.036	
	B. infantis Absent	None		Reference		
		Mixed		0.50 (0.26, 0.74)	0.000	
		Exclusive		0.97 (0.73, 1.22)	0.000	
3 Months	Overall	None	LYSOC16:0	Reference		0.156
		Mixed		0.57 (0.36, 0.77)	0.000	
		Exclusive		1.34 (1.13, 1.55)	0.000	
	B. infantis Present	None		Reference		
		Mixed		0.15 (-0.60, 0.89)	0.697	
		Exclusive		1.29 (0.55, 2.03)	0.001	
	B. infantis Absent	None		Reference		
		Mixed		0.60 (0.37, 0.82)	0.000	
		Exclusive		1.28 (1.05, 1.50)	0.000	
3 Months	Overall	None	LYSOC28:0	Reference		0.361
		Mixed		0.02 (-0.22, 0.25)	0.892	
		Exclusive		-0.21 (-0.45, 0.03)	0.080	
	B. infantis Present	None		Reference		
		Mixed		-0.74 (-1.63, 0.15)	0.102	
		Exclusive		-0.82 (-1.71, 0.07)	0.071	
	B. infantis Absent	None		Reference		
		Mixed		0.07 (-0.19, 0.33)	0.595	
		Exclusive		-0.17 (-0.43, 0.09)	0.188	
3 Months	Overall	None	Methylmalonic acid	Reference		0.999
		Mixed		0.54 (0.29, 0.78)	0.000	
		Exclusive		0.52 (0.27, 0.77)	0.000	
	B. infantis Present	None		Reference		
		Mixed		0.33 (-0.67, 1.33)	0.506	
		Exclusive		0.32 (-0.67, 1.31)	0.519	
	B. infantis Absent	None		Reference		
		Mixed		0.55 (0.29, 0.81)	0.000	
		Exclusive		0.55 (0.28, 0.81)	0.000	

Timing of Feeding and *B. infantis* Measurement	Overall and Stratification by Presence/Absence of *B. infantis*	Human Milk Feeding Status	Metabolite	Adjusted Difference in Inverse Normal Transformed Metabolite Level β (95% CI)	*P*	*P for interaction* between Feeding and *B. infantis*
3 Months	Overall	None	N-Acetyl-Asp	Reference		0.719
		Mixed		0.36 (0.12, 0.61)	0.004	
		Exclusive		0.38 (0.12, 0.63)	0.004	
	B. infantis Present	None		Reference		
		Mixed		0.03 (-0.72, 0.78)	0.938	
		Exclusive		0.48 (-0.27, 1.22)	0.203	
	B. infantis Absent	None		Reference		
		Mixed		0.40 (0.13, 0.68)	0.004	
		Exclusive		0.39 (0.11, 0.67)	0.006	
3 Months	Overall	None	N-Acetyl-His	Reference		0.197
		Mixed		-0.01 (-0.26, 0.24)	0.912	
		Exclusive		0.09 (-0.16, 0.34)	0.485	
	B. infantis Present	None		Reference		
		Mixed		-0.32 (-1.17, 0.53)	0.449	
		Exclusive		0.13 (-0.72, 0.97)	0.764	
	B. infantis Absent	None		Reference		
		Mixed		0.06 (-0.21, 0.33)	0.645	
		Exclusive		0.10 (-0.18, 0.37)	0.486	
3 Months	Overall	None	N-Acetyl-Met	Reference		0.925
		Mixed		-0.23 (-0.47, 0.01)	0.059	
		Exclusive		-0.61 (-0.85, -0.37)	0.000	
	B. infantis Present	None		Reference		
		Mixed		-0.79 (-1.56, -0.02)	0.043	
		Exclusive		-1.28 (-2.05, -0.52)	0.002	
	B. infantis Absent	None		Reference		
		Mixed		-0.17 (-0.43, 0.09)	0.188	
		Exclusive		-0.54 (-0.80, -0.28)	0.000	
3 Months	Overall	None	N-Acetyl-Pro	Reference		0.194
		Mixed		0.02 (-0.23, 0.26)	0.904	
		Exclusive		-0.16 (-0.41, 0.09)	0.206	
	B. infantis Present	None		Reference		
		Mixed		-0.52 (-1.34, 0.31)	0.214	
		Exclusive		-0.61 (-1.43, 0.21)	0.142	
	B. infantis Absent	None		Reference		
		Mixed		0.07 (-0.20, 0.34)	0.625	
		Exclusive		-0.12 (-0.40, 0.15)	0.372	
3 Months	Overall	None	N1-Acetyl-Lys	Reference		0.106
		Mixed		-0.11 (-0.35, 0.13)	0.368	
		Exclusive		-0.71 (-0.95, -0.46)	0.000	
	B. infantis Present	None		Reference		
		Mixed		-0.24 (-1.17, 0.68)	0.602	
		Exclusive		-0.39 (-1.31, 0.54)	0.404	
	B. infantis Absent	None		Reference		
		Mixed		-0.09 (-0.35, 0.18)	0.520	
		Exclusive		-0.77 (-1.03, -0.50)	0.000	
3 Months	Overall	None	N2-Acetyl-Orn	Reference		0.008
		Mixed		0.11 (-0.13, 0.36)	0.368	
		Exclusive		-0.17 (-0.41, 0.08)	0.185	
	B. infantis Present	None		Reference		
		Mixed		0.23 (-0.73, 1.20)	0.630	
		Exclusive		0.88 (-0.08, 1.84)	0.072	
	B. infantis Absent	None		Reference		
		Mixed		0.11 (-0.14, 0.36)	0.393	

Timing of Feeding and *B. infantis* Measurement	Overall and Stratification by Presence/Absence of *B. infantis*	Human Milk Feeding Status	Metabolite	Adjusted Difference in Inverse Normal Transformed Metabolite Level β (95% CI)	*P*	*P for interaction* between Feeding and *B. infantis*
		Exclusive		-0.31 (-0.56, -0.05)	0.019	
3 Months	Overall	None	Oxalacetic acid	Reference		0.159
		Mixed		-0.01 (-0.25, 0.23)	0.923	
		Exclusive		-0.13 (-0.38, 0.11)	0.283	
	B. infantis Present	None		Reference		
		Mixed		-0.48 (-1.44, 0.48)	0.323	
		Exclusive		-0.78 (-1.74, 0.17)	0.105	
	B. infantis Absent	None		Reference		
		Mixed		0.04 (-0.22, 0.29)	0.779	
		Exclusive		-0.07 (-0.33, 0.19)	0.578	
3 Months	Overall	None	PC aa C42:2	Reference		0.276
		Mixed		0.22 (-0.01, 0.45)	0.061	
		Exclusive		0.10 (-0.13, 0.33)	0.398	
	B. infantis Present	None		Reference		
		Mixed		0.33 (-0.61, 1.28)	0.481	
		Exclusive		0.48 (-0.46, 1.42)	0.306	
	B. infantis Absent	None		Reference		
		Mixed		0.19 (-0.06, 0.43)	0.133	
		Exclusive		0.02 (-0.22, 0.27)	0.846	
3 Months	Overall	None	PC ae C30:1	Reference		0.638
		Mixed		0.30 (0.08, 0.52)	0.007	
		Exclusive		0.25 (0.03, 0.47)	0.028	
	B. infantis Present	None		Reference		
		Mixed		0.08 (-0.91, 1.08)	0.869	
		Exclusive		0.10 (-0.89, 1.09)	0.840	
	B. infantis Absent	None		Reference		
		Mixed		0.32 (0.09, 0.55)	0.008	
		Exclusive		0.23 (-0.01, 0.46)	0.058	
3 Months	Overall	None	PC ae C36:2	Reference		0.520
		Mixed		0.18 (-0.05, 0.42)	0.130	
		Exclusive		0.58 (0.34, 0.82)	0.000	
	B. infantis Present	None		Reference		
		Mixed		-0.06 (-1.07, 0.94)	0.902	
		Exclusive		0.64 (-0.36, 1.64)	0.203	
	B. infantis Absent	None		Reference		
		Mixed		0.19 (-0.06, 0.45)	0.132	
		Exclusive		0.54 (0.29, 0.80)	0.000	
3 Months	Overall	None	PC ae C40:4	Reference		0.356
		Mixed		-0.10 (-0.32, 0.12)	0.361	
		Exclusive		-0.09 (-0.31, 0.13)	0.424	
	B. infantis Present	None		Reference		
		Mixed		-0.31 (-1.31, 0.68)	0.530	
		Exclusive		-0.09 (-1.08, 0.90)	0.860	
	B. infantis Absent	None		Reference		
		Mixed		-0.08 (-0.31, 0.15)	0.495	
		Exclusive		-0.14 (-0.37, 0.10)	0.249	
3 Months	Overall	None	PC38:0AA	Reference		0.724
		Mixed		0.10 (-0.11, 0.31)	0.352	
		Exclusive		0.13 (-0.08, 0.35)	0.229	
	B. infantis Present	None		Reference		
		Mixed		0.28 (-0.69, 1.25)	0.569	
		Exclusive		0.14 (-0.82, 1.11)	0.766	
	B. infantis Absent	None		Reference		

Timing of Feeding and *B. infantis* Measurement	Overall and Stratification by Presence/Absence of *B. infantis*	Human Milk Feeding Status	Metabolite	Adjusted Difference in Inverse Normal Transformed Metabolite Level β (95% CI)	*P*	*P for interaction* between Feeding and *B. infantis*
		Mixed		0.05 (-0.17, 0.28)	0.633	
		Exclusive		0.10 (-0.12, 0.33)	0.364	
3 Months	Overall	None	Succinic acid	Reference		0.106
		Mixed		0.46 (0.22, 0.70)	0.000	
		Exclusive		0.42 (0.18, 0.66)	0.001	
	B. infantis Present	None		Reference		
		Mixed		-0.17 (-0.95, 0.62)	0.673	
		Exclusive		-0.24 (-1.02, 0.54)	0.539	
	B. infantis Absent	None		Reference		
		Mixed		0.56 (0.30, 0.82)	0.000	
		Exclusive		0.55 (0.29, 0.81)	0.000	
3 Months	Overall	None	TG(16:0_37:3)	Reference		0.341
		Mixed		0.04 (-0.13, 0.21)	0.672	
		Exclusive		-0.14 (-0.31, 0.03)	0.118	
	B. infantis Present	None		Reference		
		Mixed		0.12 (-0.30, 0.54)	0.567	
		Exclusive		-0.11 (-0.53, 0.31)	0.595	
	B. infantis Absent	None		Reference		
		Mixed		0.04 (-0.15, 0.22)	0.703	
		Exclusive		-0.08 (-0.27, 0.10)	0.381	
3 Months	Overall	None	TG(16:0_38:3)	Reference		0.158
		Mixed		-0.02 (-0.20, 0.17)	0.874	
		Exclusive		0.02 (-0.17, 0.21)	0.826	
	B. infantis Present	None		Reference		
		Mixed		0.09 (-0.49, 0.66)	0.764	
		Exclusive		-0.16 (-0.73, 0.41)	0.573	
	B. infantis Absent	None		Reference		
		Mixed		-0.04 (-0.24, 0.17)	0.713	
		Exclusive		0.07 (-0.14, 0.27)	0.516	
3 Months	Overall	None	trans-4-Hydroxyproline	Reference		0.125
		Mixed		0.20 (-0.04, 0.44)	0.099	
		Exclusive		0.56 (0.32, 0.80)	0.000	
	B. infantis Present	None		Reference		
		Mixed		0.01 (-0.94, 0.97)	0.976	
		Exclusive		0.28 (-0.66, 1.23)	0.549	
	B. infantis Absent	None		Reference		
		Mixed		0.21 (-0.04, 0.46)	0.101	
		Exclusive		0.58 (0.32, 0.83)	0.000	
1-Year	Overall	None	2-Hydroxyphenylacetic acid	Reference		0.820
		Any		-0.13 (-0.25, -0.02)	0.022	
	B. infantis Present	None		Reference		
		Any		-0.27 (-0.60, 0.05)	0.096	
	B. infantis Absent	None		Reference		
		Any		-0.12 (-0.25, 0.01)	0.062	
1-Year	Overall	None	Argininic acid	Reference		0.419
		Any		-0.13 (-0.26, -0.01)	0.031	
	B. infantis Present	None		Reference		
		Any		-0.25 (-0.62, 0.13)	0.192	
	B. infantis Absent	None		Reference		
		Any		-0.09 (-0.22, 0.04)	0.183	
1-Year	Overall	None	C2	Reference		0.865
		Any		-0.07 (-0.19, 0.05)	0.245	
	B. infantis Present	None		Reference		

Timing of Feeding and *B. infantis* Measurement	Overall and Stratification by Presence/Absence of *B. infantis*	Human Milk Feeding Status	Metabolite	Adjusted Difference in Inverse Normal Transformed Metabolite Level β (95% CI)	*P*	*P for interaction* between Feeding and *B. infantis*
		Any		-0.01 (-0.37, 0.36)	0.960	
	B. infantis Absent	None		Reference		
		Any		-0.10 (-0.23, 0.03)	0.147	
1-Year	Overall	None	C3:1	Reference		0.070
		Any		-0.09 (-0.20, 0.01)	0.083	
	B. infantis Present	None		Reference		
		Any		-0.30 (-0.61, -0.00)	0.049	
	B. infantis Absent	None		Reference		
		Any		-0.04 (-0.16, 0.07)	0.449	
1-Year	Overall	None	CE(18:1)	Reference		0.375
		Any		0.00 (-0.09, 0.10)	0.920	
	B. infantis Present	None		Reference		
		Any		0.17 (-0.12, 0.46)	0.245	
	B. infantis Absent	None		Reference		
		Any		-0.03 (-0.13, 0.08)	0.602	
1-Year	Overall	None	CE(18:3)	Reference		0.721
		Any		0.04 (-0.06, 0.14)	0.487	
	B. infantis Present	None		Reference		
		Any		0.04 (-0.27, 0.35)	0.802	
	B. infantis Absent	None		Reference		
		Any		0.01 (-0.10, 0.12)	0.927	
1-Year	Overall	None	DOPA	Reference		0.589
		Any		0.03 (-0.09, 0.14)	0.640	
	B. infantis Present	None		Reference		
		Any		0.09 (-0.26, 0.44)	0.608	
	B. infantis Absent	None		Reference		
		Any		0.00 (-0.13, 0.13)	0.963	
1-Year	Overall	None	Diacetylspermine	Reference		0.413
		Any		0.35 (0.23, 0.47)	0.000	
	B. infantis Present	None		Reference		
		Any		0.52 (0.19, 0.86)	0.002	
	B. infantis Absent	None		Reference		
		Any		0.32 (0.19, 0.46)	0.000	
1-Year	Overall	None	Glutamic acid	Reference		0.963
		Any		-0.25 (-0.37, -0.13)	0.000	
	B. infantis Present	None		Reference		
		Any		-0.10 (-0.46, 0.25)	0.558	
	B. infantis Absent	None		Reference		
		Any		-0.22 (-0.36, -0.09)	0.001	
1-Year	Overall	None	Hex3Cer(d18:1/18:0)	Reference		0.800
		Any		0.20 (0.11, 0.29)	0.000	
	B. infantis Present	None		Reference		
		Any		0.20 (-0.09, 0.48)	0.169	
	B. infantis Absent	None		Reference		
		Any		0.21 (0.10, 0.31)	0.000	
1-Year	Overall	None	Isopropanol	Reference		0.051
		Any		0.14 (0.01, 0.26)	0.028	
	B. infantis Present	None		Reference		
		Any		0.53 (0.16, 0.90)	0.005	
	B. infantis Absent	None		Reference		
		Any		0.07 (-0.06, 0.21)	0.300	
1-Year	Overall	None	Lysine	Reference		0.474
		Any		-0.54 (-0.66, -0.42)	0.000	

Timing of Feeding and *B. infantis* Measurement	Overall and Stratification by Presence/Absence of *B. infantis*	Human Milk Feeding Status	Metabolite	Adjusted Difference in Inverse Normal Transformed Metabolite Level β (95% CI)	*P*	*P for interaction* between Feeding and *B. infantis*
	B. infantis Present	None		Reference		
		Any		-0.39 (-0.71, -0.07)	0.017	
	B. infantis Absent	None		Reference		
		Any		-0.51 (-0.64, -0.38)	0.000	
1-Year	Overall	None	N1-Acetylspermidine	Reference		0.697
		Any		0.08 (-0.04, 0.20)	0.201	
	B. infantis Present	None		Reference		
		Any		0.14 (-0.22, 0.51)	0.436	
	B. infantis Absent	None		Reference		
		Any		0.12 (-0.02, 0.25)	0.088	
1-Year	Overall	None	Nicotinic-acid	Reference		0.820
		Any		0.04 (-0.08, 0.16)	0.544	
	B. infantis Present	None		Reference		
		Any		-0.05 (-0.44, 0.34)	0.793	
	B. infantis Absent	None		Reference		
		Any		0.08 (-0.06, 0.21)	0.268	
1-Year	Overall	None	PC ae C44:4	Reference		0.087
		Any		0.13 (0.02, 0.24)	0.019	
	B. infantis Present	None		Reference		
		Any		0.40 (0.06, 0.73)	0.022	
	B. infantis Absent	None		Reference		
		Any		0.07 (-0.05, 0.19)	0.241	

Estimates are based on mixed-effect linear regressions with metabolite levels transformed by rank-based inverse normal transformation as the outcome. SBP-related metabolites are identified by elastic net regression in the overall sample or within each stratum of feeding status (shown in eTable 5). Results with P for interaction<0.1 are further visualized in Figure 2. Models adjusted for maternal age at delivery (continuous), pre-pregnancy body mass index (continuous), educational achievement (high school or below, college and university level, graduate degree), hypertension (chronic, during pregnancy, no), antibiotic use (prenatal, intrapartum, no), delivery mode (vaginal, C-section with labor, C-section without labor), child sex (boy, girl), birth weight (continuous), exact age in months (continuous) at fecal sample collection, time between sample collection and freezing (hours), with study site (Edmonton, Toronto, Vancouver, Winnipeg) as a random intercept.

eTable 8. Associations between presence/absence of *B. infantis* and SBP-related fecal metabolites at 3 months and 1 year, overall and by human milk feeding status.

Timing of Feeding and *B. infantis* Measurement	Overall and Stratification by Human Milk Feeding Status	Presence/Absence of *B. infantis*	Metabolite	Adjusted Difference in Inverse Normal Transformed Metabolite Level β (95% CI)	*P*	*P for interaction* between Feeding and *B. infantis*
3 Months	Overall	*B. infantis* Absent	16:1SMOH	Reference		0.528
		B. infantis Present		0.18 (-0.08, 0.44)	0.171	
	Exclusive	*B. infantis* Absent		Reference		
		B. infantis Present		0.16 (-0.26, 0.57)	0.450	
	Mixed	*B. infantis* Absent		Reference		
		B. infantis Present		-0.07 (-0.49, 0.36)	0.764	
	None	*B. infantis* Absent		Reference		
		B. infantis Present		0.09 (-0.55, 0.73)	0.775	
3 Months	Overall	*B. infantis* Absent	2-Hydroxy-3-methylvaleric acid	Reference		0.619
		B. infantis Present		-0.05 (-0.32, 0.23)	0.747	
	Exclusive	*B. infantis* Absent		Reference		
		B. infantis Present		-0.22 (-0.66, 0.21)	0.304	
	Mixed	*B. infantis* Absent		Reference		
		B. infantis Present		-0.06 (-0.50, 0.39)	0.801	
	None	*B. infantis* Absent		Reference		
		B. infantis Present		0.42 (-0.37, 1.22)	0.292	
3 Months	Overall	*B. infantis* Absent	22:1SMOH	Reference		0.336
		B. infantis Present		0.05 (-0.22, 0.32)	0.710	
	Exclusive	*B. infantis* Absent		Reference		
		B. infantis Present		0.03 (-0.36, 0.43)	0.862	
	Mixed	*B. infantis* Absent		Reference		
		B. infantis Present		-0.21 (-0.67, 0.26)	0.379	
	None	*B. infantis* Absent		Reference		
		B. infantis Present		0.63 (-0.13, 1.39)	0.103	
3 Months	Overall	*B. infantis* Absent	22:2SMOH	Reference		0.927
		B. infantis Present		0.19 (-0.06, 0.45)	0.134	
	Exclusive	*B. infantis* Absent		Reference		
		B. infantis Present		0.04 (-0.34, 0.41)	0.855	
	Mixed	*B. infantis* Absent		Reference		
		B. infantis Present		0.17 (-0.23, 0.57)	0.405	
	None	*B. infantis* Absent		Reference		
		B. infantis Present		0.20 (-0.53, 0.93)	0.587	
3 Months	Overall	*B. infantis* Absent	3-Hydroxyisobutyric acid	Reference		0.853
		B. infantis Present		-0.22 (-0.48, 0.05)	0.107	
	Exclusive	*B. infantis* Absent		Reference		
		B. infantis Present		-0.25 (-0.63, 0.13)	0.196	
	Mixed	*B. infantis* Absent		Reference		
		B. infantis Present		-0.14 (-0.60, 0.31)	0.543	
	None	*B. infantis* Absent		Reference		
		B. infantis Present		-0.01 (-0.74, 0.71)	0.973	
3 Months	Overall	*B. infantis* Absent	4-Hydroxybenzoic acid	Reference		0.870
		B. infantis Present		-0.33 (-0.59, -0.08)	0.011	
	Exclusive	*B. infantis* Absent		Reference		
		B. infantis Present		-0.42 (-0.80, -0.03)	0.035	
	Mixed	*B. infantis* Absent		Reference		
		B. infantis Present		-0.38 (-0.80, 0.04)	0.079	
	None	*B. infantis* Absent		Reference		
		B. infantis Present		-0.29 (-1.00, 0.42)	0.420	
3 Months	Overall	*B. infantis* Absent		Reference		0.307
		B. infantis Present		-0.25 (-0.52, 0.02)	0.064	

Timing of Feeding and *B. infantis* Measurement	Overall and Stratification by Human Milk Feeding Status	Presence/Absence of *B. infantis*	Metabolite	Adjusted Difference in Inverse Normal Transformed Metabolite Level β (95% CI)	*P*	*P for interaction* between Feeding and *B. infantis*
	Exclusive	*B. infantis* Absent	4-Hydroxyphenylacetic acid	Reference		
		B. infantis Present		-0.28 (-0.63, 0.08)	0.127	
	Mixed	*B. infantis* Absent		Reference		
		B. infantis Present		0.04 (-0.40, 0.48)	0.853	
	None	*B. infantis* Absent		Reference		
		B. infantis Present		-0.66 (-1.48, 0.15)	0.110	
3 Months	Overall	*B. infantis* Absent	5-Hydroxylysine	Reference		0.851
		B. infantis Present		-0.08 (-0.35, 0.19)	0.545	
	Exclusive	*B. infantis* Absent		Reference		
		B. infantis Present		-0.06 (-0.43, 0.31)	0.763	
	Mixed	*B. infantis* Absent		Reference		
		B. infantis Present		0.13 (-0.32, 0.58)	0.564	
	None	*B. infantis* Absent		Reference		
		B. infantis Present		-0.04 (-0.75, 0.67)	0.910	
3 Months	Overall	*B. infantis* Absent	Agmatine	Reference		0.682
		B. infantis Present		0.04 (-0.23, 0.31)	0.758	
	Exclusive	*B. infantis* Absent		Reference		
		B. infantis Present		-0.09 (-0.49, 0.32)	0.669	
	Mixed	*B. infantis* Absent		Reference		
		B. infantis Present		0.06 (-0.38, 0.51)	0.782	
	None	*B. infantis* Absent		Reference		
		B. infantis Present		0.30 (-0.43, 1.03)	0.420	
3 Months	Overall	*B. infantis* Absent	Arabinose	Reference		0.103
		B. infantis Present		-0.04 (-0.35, 0.26)	0.778	
	Exclusive	*B. infantis* Absent		Reference		
		B. infantis Present		0.23 (-0.34, 0.79)	0.428	
	Mixed	*B. infantis* Absent		Reference		
		B. infantis Present		-0.50 (-0.99, -0.01)	0.046	
	None	*B. infantis* Absent		Reference		
		B. infantis Present		0.07 (-0.68, 0.81)	0.859	
3 Months	Overall	*B. infantis* Absent	C0	Reference		0.341
		B. infantis Present		0.39 (0.12, 0.66)	0.005	
	Exclusive	*B. infantis* Absent		Reference		
		B. infantis Present		0.32 (-0.06, 0.70)	0.094	
	Mixed	*B. infantis* Absent		Reference		
		B. infantis Present		0.32 (-0.14, 0.78)	0.165	
	None	*B. infantis* Absent		Reference		
		B. infantis Present		1.04 (0.24, 1.84)	0.012	
3 Months	Overall	*B. infantis* Absent	C12DC	Reference		0.830
		B. infantis Present		0.03 (-0.24, 0.30)	0.844	
	Exclusive	*B. infantis* Absent		Reference		
		B. infantis Present		0.12 (-0.24, 0.49)	0.506	
	Mixed	*B. infantis* Absent		Reference		
		B. infantis Present		0.20 (-0.27, 0.67)	0.394	
	None	*B. infantis* Absent		Reference		
		B. infantis Present		0.21 (-0.62, 1.03)	0.621	
3 Months	Overall	*B. infantis* Absent	C14	Reference		0.437
		B. infantis Present		0.07 (-0.19, 0.33)	0.585	
	Exclusive	*B. infantis* Absent		Reference		
		B. infantis Present		-0.07 (-0.50, 0.35)	0.728	
	Mixed	*B. infantis* Absent		Reference		
		B. infantis Present		0.08 (-0.33, 0.48)	0.704	
	None	*B. infantis* Absent		Reference		

Timing of Feeding and *B. infantis* Measurement	Overall and Stratification by Human Milk Feeding Status	Presence/Absence of *B. infantis*	Metabolite	Adjusted Difference in Inverse Normal Transformed Metabolite Level β (95% CI)	*P*	*P for interaction between Feeding and B. infantis*
		B. infantis Present		0.57 (-0.03, 1.17)	0.060	
3 Months	Overall	*B. infantis* Absent	C16OH	Reference		0.474
		B. infantis Present		0.30 (0.07, 0.52)	0.010	
	Exclusive	*B. infantis* Absent		Reference		
		B. infantis Present		0.27 (-0.10, 0.65)	0.155	
	Mixed	*B. infantis* Absent		Reference		
		B. infantis Present		0.28 (-0.07, 0.64)	0.120	
	None	*B. infantis* Absent		Reference		
		B. infantis Present		0.65 (0.05, 1.25)	0.033	
3 Months	Overall	*B. infantis* Absent	C18	Reference		0.329
		B. infantis Present		0.06 (-0.21, 0.33)	0.664	
	Exclusive	*B. infantis* Absent		Reference		
		B. infantis Present		-0.04 (-0.48, 0.40)	0.852	
	Mixed	*B. infantis* Absent		Reference		
		B. infantis Present		-0.08 (-0.52, 0.35)	0.699	
	None	*B. infantis* Absent		Reference		
		B. infantis Present		0.55 (-0.06, 1.17)	0.078	
3 Months	Overall	*B. infantis* Absent	C4OH	Reference		0.101
		B. infantis Present		-0.18 (-0.45, 0.09)	0.196	
	Exclusive	*B. infantis* Absent		Reference		
		B. infantis Present		-0.43 (-0.79, -0.07)	0.020	
	Mixed	*B. infantis* Absent		Reference		
		B. infantis Present		0.13 (-0.32, 0.57)	0.574	
	None	*B. infantis* Absent		Reference		
		B. infantis Present		0.39 (-0.49, 1.26)	0.381	
3 Months	Overall	*B. infantis* Absent	C5	Reference		0.778
		B. infantis Present		0.14 (-0.13, 0.41)	0.309	
	Exclusive	*B. infantis* Absent		Reference		
		B. infantis Present		0.12 (-0.21, 0.46)	0.467	
	Mixed	*B. infantis* Absent		Reference		
		B. infantis Present		0.28 (-0.16, 0.72)	0.210	
	None	*B. infantis* Absent		Reference		
		B. infantis Present		0.60 (-0.38, 1.57)	0.226	
3 Months	Overall	*B. infantis* Absent	C5OH	Reference		0.636
		B. infantis Present		0.23 (-0.04, 0.50)	0.096	
	Exclusive	*B. infantis* Absent		Reference		
		B. infantis Present		0.10 (-0.27, 0.46)	0.610	
	Mixed	*B. infantis* Absent		Reference		
		B. infantis Present		0.42 (-0.05, 0.89)	0.082	
	None	*B. infantis* Absent		Reference		
		B. infantis Present		0.56 (-0.24, 1.36)	0.169	
3 Months	Overall	*B. infantis* Absent	CE(15:1)	Reference		0.038
		B. infantis Present		0.09 (-0.11, 0.29)	0.395	
	Exclusive	*B. infantis* Absent		Reference		
		B. infantis Present		-0.18 (-0.46, 0.09)	0.195	
	Mixed	*B. infantis* Absent		Reference		
		B. infantis Present		0.32 (-0.04, 0.69)	0.079	
	None	*B. infantis* Absent		Reference		
		B. infantis Present		-0.01 (-0.62, 0.60)	0.973	
3 Months	Overall	*B. infantis* Absent	CE(18:0)	Reference		0.429
		B. infantis Present		-0.04 (-0.28, 0.19)	0.727	
	Exclusive	*B. infantis* Absent		Reference		
		B. infantis Present		0.03 (-0.33, 0.39)	0.853	

Timing of Feeding and *B. infantis* Measurement	Overall and Stratification by Human Milk Feeding Status	Presence/Absence of *B. infantis*	Metabolite	Adjusted Difference in Inverse Normal Transformed Metabolite Level β (95% CI)	*P*	*P for interaction* between Feeding and *B. infantis*
	Mixed	*B. infantis* Absent		Reference		
		B. infantis Present		0.04 (-0.36, 0.45)	0.832	
	None	*B. infantis* Absent		Reference		
		B. infantis Present		-0.38 (-1.08, 0.32)	0.286	
3 Months	Overall	*B. infantis* Absent	CE(18:1)	Reference		0.415
		B. infantis Present		-0.00 (-0.24, 0.23)	0.969	
	Exclusive	*B. infantis* Absent		Reference		
		B. infantis Present		0.02 (-0.33, 0.37)	0.918	
	Mixed	*B. infantis* Absent		Reference		
		B. infantis Present		0.13 (-0.26, 0.53)	0.498	
	None	*B. infantis* Absent		Reference		
		B. infantis Present		-0.31 (-1.00, 0.38)	0.377	
3 Months	Overall	*B. infantis* Absent	CE(20:0)	Reference		0.400
		B. infantis Present		0.00 (-0.21, 0.22)	0.981	
	Exclusive	*B. infantis* Absent		Reference		
		B. infantis Present		-0.17 (-0.49, 0.15)	0.296	
	Mixed	*B. infantis* Absent		Reference		
		B. infantis Present		0.08 (-0.28, 0.45)	0.652	
	None	*B. infantis* Absent		Reference		
		B. infantis Present		0.04 (-0.63, 0.71)	0.902	
3 Months	Overall	*B. infantis* Absent	Cer(d18:1/16:0)	Reference		0.515
		B. infantis Present		0.01 (-0.26, 0.28)	0.930	
	Exclusive	*B. infantis* Absent		Reference		
		B. infantis Present		-0.15 (-0.55, 0.25)	0.459	
	Mixed	*B. infantis* Absent		Reference		
		B. infantis Present		0.04 (-0.45, 0.54)	0.859	
	None	*B. infantis* Absent		Reference		
		B. infantis Present		0.41 (-0.37, 1.19)	0.300	
3 Months	Overall	*B. infantis* Absent	Cer(d18:1/24:0)	Reference		0.526
		B. infantis Present		-0.22 (-0.48, 0.05)	0.117	
	Exclusive	*B. infantis* Absent		Reference		
		B. infantis Present		-0.34 (-0.75, 0.06)	0.096	
	Mixed	*B. infantis* Absent		Reference		
		B. infantis Present		-0.21 (-0.67, 0.26)	0.383	
	None	*B. infantis* Absent		Reference		
		B. infantis Present		0.12 (-0.67, 0.91)	0.762	
3 Months	Overall	*B. infantis* Absent	Creatinine	Reference		0.093
		B. infantis Present		0.59 (0.35, 0.84)	0.000	
	Exclusive	*B. infantis* Absent		Reference		
		B. infantis Present		0.85 (0.49, 1.20)	0.000	
	Mixed	*B. infantis* Absent		Reference		
		B. infantis Present		0.46 (0.03, 0.89)	0.034	
	None	*B. infantis* Absent		Reference		
		B. infantis Present		0.41 (-0.25, 1.07)	0.223	
3 Months	Overall	*B. infantis* Absent	DG(16:1_20:0)	Reference		0.569
		B. infantis Present		0.19 (-0.04, 0.43)	0.111	
	Exclusive	*B. infantis* Absent		Reference		
		B. infantis Present		0.08 (-0.25, 0.41)	0.626	
	Mixed	*B. infantis* Absent		Reference		
		B. infantis Present		0.31 (-0.10, 0.73)	0.137	
	None	*B. infantis* Absent		Reference		
		B. infantis Present		0.02 (-0.67, 0.72)	0.951	
3 Months	Overall	*B. infantis* Absent	DOPA	Reference		0.268

Timing of Feeding and *B. infantis* Measurement	Overall and Stratification by Human Milk Feeding Status	Presence/Absence of *B. infantis*	Metabolite	Adjusted Difference in Inverse Normal Transformed Metabolite Level β (95% CI)	*P*	*P for interaction* between Feeding and *B. infantis*
		B. infantis Present		-0.04 (-0.27, 0.20)	0.769	
	Exclusive	*B. infantis* Absent		Reference		
		B. infantis Present		0.16 (-0.19, 0.50)	0.373	
	Mixed	*B. infantis* Absent		Reference		
		B. infantis Present		-0.16 (-0.54, 0.22)	0.414	
	None	*B. infantis* Absent		Reference		
		B. infantis Present		-0.10 (-0.83, 0.63)	0.785	
3 Months	Overall	*B. infantis* Absent	Guanidinopropionic acid	Reference		0.909
		B. infantis Present		0.40 (0.14, 0.67)	0.003	
	Exclusive	*B. infantis* Absent		Reference		
		B. infantis Present		0.36 (0.03, 0.69)	0.032	
	Mixed	*B. infantis* Absent		Reference		
		B. infantis Present		0.41 (-0.09, 0.91)	0.110	
	None	*B. infantis* Absent		Reference		
		B. infantis Present		0.57 (-0.27, 1.41)	0.179	
3 Months	Overall	*B. infantis* Absent	Hex2Cer(d18:1/14:0)	Reference		0.279
		B. infantis Present		0.25 (-0.02, 0.52)	0.068	
	Exclusive	*B. infantis* Absent		Reference		
		B. infantis Present		0.17 (-0.21, 0.55)	0.384	
	Mixed	*B. infantis* Absent		Reference		
		B. infantis Present		-0.07 (-0.56, 0.42)	0.790	
	None	*B. infantis* Absent		Reference		
		B. infantis Present		0.75 (0.04, 1.46)	0.038	
3 Months	Overall	*B. infantis* Absent	HexCer(d18:1/24:0)	Reference		0.276
		B. infantis Present		-0.03 (-0.30, 0.24)	0.837	
	Exclusive	*B. infantis* Absent		Reference		
		B. infantis Present		-0.22 (-0.61, 0.17)	0.272	
	Mixed	*B. infantis* Absent		Reference		
		B. infantis Present		-0.08 (-0.53, 0.36)	0.714	
	None	*B. infantis* Absent		Reference		
		B. infantis Present		0.61 (-0.11, 1.33)	0.096	
3 Months	Overall	*B. infantis* Absent	Histamine	Reference		0.990
		B. infantis Present		-0.04 (-0.31, 0.24)	0.784	
	Exclusive	*B. infantis* Absent		Reference		
		B. infantis Present		-0.15 (-0.58, 0.28)	0.485	
	Mixed	*B. infantis* Absent		Reference		
		B. infantis Present		-0.05 (-0.52, 0.41)	0.819	
	None	*B. infantis* Absent		Reference		
		B. infantis Present		0.06 (-0.68, 0.80)	0.870	
3 Months	Overall	*B. infantis* Absent	LYSOC14:0	Reference		0.058
		B. infantis Present		0.22 (-0.04, 0.49)	0.098	
	Exclusive	*B. infantis* Absent		Reference		
		B. infantis Present		0.28 (-0.09, 0.64)	0.137	
	Mixed	*B. infantis* Absent		Reference		
		B. infantis Present		-0.24 (-0.67, 0.18)	0.262	
	None	*B. infantis* Absent		Reference		
		B. infantis Present		0.69 (0.05, 1.34)	0.036	
3 Months	Overall	*B. infantis* Absent	LYSOC16:0	Reference		0.156
		B. infantis Present		0.40 (0.14, 0.67)	0.003	
	Exclusive	*B. infantis* Absent		Reference		
		B. infantis Present		0.43 (0.09, 0.77)	0.013	
	Mixed	*B. infantis* Absent		Reference		
		B. infantis Present		-0.05 (-0.43, 0.33)	0.788	

Timing of Feeding and *B. infantis* Measurement	Overall and Stratification by Human Milk Feeding Status	Presence/Absence of *B. infantis*	Metabolite	Adjusted Difference in Inverse Normal Transformed Metabolite Level β (95% CI)	*P*	*P for interaction* between Feeding and *B. infantis*
	None	*B. infantis* Absent		Reference		
		B. infantis Present		0.76 (0.13, 1.40)	0.019	
3 Months	Overall	*B. infantis* Absent	LYSOC28:0	Reference		0.361
		B. infantis Present		0.00 (-0.26, 0.26)	0.997	
	Exclusive	*B. infantis* Absent		Reference		
		B. infantis Present		-0.10 (-0.53, 0.33)	0.639	
	Mixed	*B. infantis* Absent		Reference		
		B. infantis Present		-0.11 (-0.55, 0.32)	0.605	
	None	*B. infantis* Absent		Reference		
		B. infantis Present		0.70 (0.05, 1.35)	0.035	
3 Months	Overall	*B. infantis* Absent	Methylmalonic acid	Reference		0.999
		B. infantis Present		-0.17 (-0.45, 0.10)	0.212	
	Exclusive	*B. infantis* Absent		Reference		
		B. infantis Present		-0.33 (-0.76, 0.11)	0.140	
	Mixed	*B. infantis* Absent		Reference		
		B. infantis Present		-0.29 (-0.75, 0.18)	0.226	
	None	*B. infantis* Absent		Reference		
		B. infantis Present		-0.27 (-0.91, 0.37)	0.408	
3 Months	Overall	*B. infantis* Absent	N-Acetyl-Asp	Reference		0.719
		B. infantis Present		-0.05 (-0.32, 0.23)	0.737	
	Exclusive	*B. infantis* Absent		Reference		
		B. infantis Present		-0.12 (-0.57, 0.34)	0.617	
	Mixed	*B. infantis* Absent		Reference		
		B. infantis Present		-0.16 (-0.60, 0.28)	0.483	
	None	*B. infantis* Absent		Reference		
		B. infantis Present		0.06 (-0.66, 0.77)	0.876	
3 Months	Overall	*B. infantis* Absent	N-Acetyl-His	Reference		0.197
		B. infantis Present		0.18 (-0.09, 0.46)	0.188	
	Exclusive	*B. infantis* Absent		Reference		
		B. infantis Present		0.17 (-0.24, 0.58)	0.416	
	Mixed	*B. infantis* Absent		Reference		
		B. infantis Present		-0.03 (-0.50, 0.43)	0.884	
	None	*B. infantis* Absent		Reference		
		B. infantis Present		0.73 (-0.04, 1.50)	0.064	
3 Months	Overall	*B. infantis* Absent	N-Acetyl-Met	Reference		0.925
		B. infantis Present		-0.19 (-0.46, 0.08)	0.166	
	Exclusive	*B. infantis* Absent		Reference		
		B. infantis Present		-0.22 (-0.57, 0.12)	0.208	
	Mixed	*B. infantis* Absent		Reference		
		B. infantis Present		-0.09 (-0.56, 0.37)	0.691	
	None	*B. infantis* Absent		Reference		
		B. infantis Present		-0.01 (-0.87, 0.85)	0.981	
3 Months	Overall	*B. infantis* Absent	N-Acetyl-Pro	Reference		0.194
		B. infantis Present		0.10 (-0.18, 0.37)	0.485	
	Exclusive	*B. infantis* Absent		Reference		
		B. infantis Present		-0.10 (-0.52, 0.31)	0.628	
	Mixed	*B. infantis* Absent		Reference		
		B. infantis Present		0.01 (-0.43, 0.45)	0.954	
	None	*B. infantis* Absent		Reference		
		B. infantis Present		0.90 (0.08, 1.72)	0.031	
3 Months	Overall	*B. infantis* Absent	N1-Acetyl-Lys	Reference		0.106
		B. infantis Present		0.04 (-0.24, 0.32)	0.783	
	Exclusive	*B. infantis* Absent		Reference		

Timing of Feeding and *B. infantis* Measurement	Overall and Stratification by Human Milk Feeding Status	Presence/Absence of *B. infantis*	Metabolite	Adjusted Difference in Inverse Normal Transformed Metabolite Level β (95% CI)	*P*	*P for interaction* between Feeding and *B. infantis*
		B. infantis Present		0.30 (-0.06, 0.66)	0.106	
	Mixed	*B. infantis* Absent		Reference		
		B. infantis Present		-0.14 (-0.61, 0.34)	0.574	
	None	*B. infantis* Absent		Reference		
		B. infantis Present		0.06 (-0.74, 0.87)	0.874	
3 Months	Overall	*B. infantis* Absent	N2-Acetyl-Orn	Reference		0.008
		B. infantis Present		0.29 (0.02, 0.56)	0.036	
	Exclusive	*B. infantis* Absent		Reference		
		B. infantis Present		0.71 (0.28, 1.15)	0.002	
	Mixed	*B. infantis* Absent		Reference		
		B. infantis Present		-0.06 (-0.47, 0.35)	0.769	
	None	*B. infantis* Absent		Reference		
		B. infantis Present		-0.10 (-0.89, 0.68)	0.793	
3 Months	Overall	*B. infantis* Absent	Oxalacetic acid	Reference		0.159
		B. infantis Present		0.00 (-0.26, 0.27)	0.979	
	Exclusive	*B. infantis* Absent		Reference		
		B. infantis Present		-0.14 (-0.54, 0.27)	0.514	
	Mixed	*B. infantis* Absent		Reference		
		B. infantis Present		-0.12 (-0.58, 0.35)	0.617	
	None	*B. infantis* Absent		Reference		
		B. infantis Present		0.62 (-0.09, 1.33)	0.087	
3 Months	Overall	*B. infantis* Absent	PC aa C42:2	Reference		0.276
		B. infantis Present		-0.05 (-0.30, 0.20)	0.711	
	Exclusive	*B. infantis* Absent		Reference		
		B. infantis Present		0.09 (-0.29, 0.48)	0.636	
	Mixed	*B. infantis* Absent		Reference		
		B. infantis Present		-0.21 (-0.64, 0.21)	0.325	
	None	*B. infantis* Absent		Reference		
		B. infantis Present		-0.28 (-0.96, 0.40)	0.412	
3 Months	Overall	*B. infantis* Absent	PC ae C30:1	Reference		0.638
		B. infantis Present		0.12 (-0.12, 0.36)	0.325	
	Exclusive	*B. infantis* Absent		Reference		
		B. infantis Present		0.12 (-0.25, 0.49)	0.522	
	Mixed	*B. infantis* Absent		Reference		
		B. infantis Present		-0.08 (-0.51, 0.34)	0.698	
	None	*B. infantis* Absent		Reference		
		B. infantis Present		0.31 (-0.37, 0.99)	0.365	
3 Months	Overall	*B. infantis* Absent	PC ae C36:2	Reference		0.520
		B. infantis Present		0.23 (-0.04, 0.49)	0.098	
	Exclusive	*B. infantis* Absent		Reference		
		B. infantis Present		0.20 (-0.19, 0.58)	0.313	
	Mixed	*B. infantis* Absent		Reference		
		B. infantis Present		-0.09 (-0.58, 0.39)	0.699	
	None	*B. infantis* Absent		Reference		
		B. infantis Present		0.35 (-0.28, 0.97)	0.277	
3 Months	Overall	*B. infantis* Absent	PC ae C40:4	Reference		0.356
		B. infantis Present		0.17 (-0.07, 0.41)	0.167	
	Exclusive	*B. infantis* Absent		Reference		
		B. infantis Present		0.33 (-0.05, 0.72)	0.091	
	Mixed	*B. infantis* Absent		Reference		
		B. infantis Present		-0.12 (-0.50, 0.27)	0.551	
	None	*B. infantis* Absent		Reference		
		B. infantis Present		0.28 (-0.38, 0.95)	0.401	

Timing of Feeding and *B. infantis* Measurement	Overall and Stratification by Human Milk Feeding Status	Presence/Absence of *B. infantis*	Metabolite	Adjusted Difference in Inverse Normal Transformed Metabolite Level β (95% CI)	*P*	*P for interaction between Feeding and B. infantis*
3 Months	Overall	*B. infantis* Absent	PC38:0AA	Reference		0.724
		B. infantis Present		0.04 (-0.20, 0.27)	0.766	
	Exclusive	*B. infantis* Absent		Reference		
		B. infantis Present		-0.10 (-0.48, 0.27)	0.586	
	Mixed	*B. infantis* Absent		Reference		
		B. infantis Present		0.07 (-0.32, 0.47)	0.722	
	None	*B. infantis* Absent		Reference		
		B. infantis Present		-0.12 (-0.77, 0.53)	0.710	
3 Months	Overall	*B. infantis* Absent	Succinic acid	Reference		0.106
		B. infantis Present		-0.31 (-0.58, -0.04)	0.023	
	Exclusive	*B. infantis* Absent		Reference		
		B. infantis Present		-0.57 (-0.94, -0.19)	0.003	
	Mixed	*B. infantis* Absent		Reference		
		B. infantis Present		-0.37 (-0.80, 0.07)	0.096	
	None	*B. infantis* Absent		Reference		
		B. infantis Present		0.05 (-0.73, 0.84)	0.893	
3 Months	Overall	*B. infantis* Absent	TG(16:0_37:3)	Reference		0.341
		B. infantis Present		-0.17 (-0.35, 0.02)	0.078	
	Exclusive	*B. infantis* Absent		Reference		
		B. infantis Present		-0.30 (-0.54, -0.06)	0.016	
	Mixed	*B. infantis* Absent		Reference		
		B. infantis Present		-0.04 (-0.37, 0.28)	0.808	
	None	*B. infantis* Absent		Reference		
		B. infantis Present		-0.11 (-0.74, 0.52)	0.720	
3 Months	Overall	*B. infantis* Absent	TG(16:0_38:3)	Reference		0.158
		B. infantis Present		-0.15 (-0.35, 0.06)	0.155	
	Exclusive	*B. infantis* Absent		Reference		
		B. infantis Present		-0.34 (-0.66, -0.03)	0.031	
	Mixed	*B. infantis* Absent		Reference		
		B. infantis Present		0.04 (-0.30, 0.38)	0.815	
	None	*B. infantis* Absent		Reference		
		B. infantis Present		-0.10 (-0.69, 0.50)	0.743	
3 Months	Overall	*B. infantis* Absent	trans-4-Hydroxyproline	Reference		0.125
		B. infantis Present		0.23 (-0.04, 0.50)	0.091	
	Exclusive	*B. infantis* Absent		Reference		
		B. infantis Present		-0.06 (-0.43, 0.32)	0.774	
	Mixed	*B. infantis* Absent		Reference		
		B. infantis Present		0.15 (-0.31, 0.61)	0.512	
	None	*B. infantis* Absent		Reference		
		B. infantis Present		0.80 (0.00, 1.60)	0.049	
1-Year	Overall	*B. infantis* Absent	2-Hydroxyphenylacetic acid	Reference		0.820
		B. infantis Present		-0.07 (-0.22, 0.07)	0.310	
	Any	*B. infantis* Absent		Reference		
		B. infantis Present		-0.02 (-0.20, 0.16)	0.842	
	None	*B. infantis* Absent		Reference		
		B. infantis Present		-0.05 (-0.33, 0.23)	0.724	
1-Year	Overall	*B. infantis* Absent	Argininic acid	Reference		0.419
		B. infantis Present		-0.14 (-0.29, 0.01)	0.063	
	Any	*B. infantis* Absent		Reference		
		B. infantis Present		-0.14 (-0.33, 0.06)	0.162	
	None	*B. infantis* Absent		Reference		
		B. infantis Present		-0.03 (-0.32, 0.25)	0.819	
1-Year	Overall	*B. infantis* Absent	C2	Reference		0.865

Timing of Feeding and *B. infantis* Measurement	Overall and Stratification by Human Milk Feeding Status	Presence/Absence of *B. infantis*	Metabolite	Adjusted Difference in Inverse Normal Transformed Metabolite Level β (95% CI)	*P*	*P for interaction* between Feeding and *B. infantis*
		B. infantis Present		0.10 (-0.05, 0.25)	0.201	
	Any	*B. infantis* Absent		Reference		
		B. infantis Present		0.13 (-0.07, 0.33)	0.217	
	None	*B. infantis* Absent		Reference		
		B. infantis Present		0.15 (-0.12, 0.42)	0.279	
1-Year	Overall	*B. infantis* Absent	C3:1	Reference		0.070
		B. infantis Present		-0.07 (-0.20, 0.05)	0.262	
	Any	*B. infantis* Absent		Reference		
		B. infantis Present		-0.15 (-0.31, 0.01)	0.069	
	None	*B. infantis* Absent		Reference		
		B. infantis Present		0.15 (-0.10, 0.39)	0.244	
1-Year	Overall	*B. infantis* Absent	CE(18:1)	Reference		0.375
		B. infantis Present		0.05 (-0.07, 0.17)	0.379	
	Any	*B. infantis* Absent		Reference		
		B. infantis Present		0.10 (-0.06, 0.25)	0.209	
	None	*B. infantis* Absent		Reference		
		B. infantis Present		-0.05 (-0.28, 0.17)	0.635	
1-Year	Overall	*B. infantis* Absent	CE(18:3)	Reference		0.721
		B. infantis Present		0.20 (0.07, 0.32)	0.002	
	Any	*B. infantis* Absent		Reference		
		B. infantis Present		0.17 (0.01, 0.33)	0.036	
	None	*B. infantis* Absent		Reference		
		B. infantis Present		0.20 (-0.03, 0.43)	0.090	
1-Year	Overall	*B. infantis* Absent	DOPA	Reference		0.589
		B. infantis Present		0.06 (-0.09, 0.20)	0.430	
	Any	*B. infantis* Absent		Reference		
		B. infantis Present		0.10 (-0.09, 0.29)	0.299	
	None	*B. infantis* Absent		Reference		
		B. infantis Present		-0.02 (-0.29, 0.25)	0.891	
1-Year	Overall	*B. infantis* Absent	Diacetylspermine	Reference		0.413
		B. infantis Present		0.10 (-0.05, 0.25)	0.173	
	Any	*B. infantis* Absent		Reference		
		B. infantis Present		0.03 (-0.16, 0.23)	0.735	
	None	*B. infantis* Absent		Reference		
		B. infantis Present		-0.06 (-0.34, 0.21)	0.644	
1-Year	Overall	*B. infantis* Absent	Glutamic acid	Reference		0.963
		B. infantis Present		-0.23 (-0.38, -0.08)	0.003	
	Any	*B. infantis* Absent		Reference		
		B. infantis Present		-0.18 (-0.38, 0.02)	0.083	
	None	*B. infantis* Absent		Reference		
		B. infantis Present		-0.19 (-0.46, 0.08)	0.169	
1-Year	Overall	*B. infantis* Absent	Hex3Cer(d18:1/18:0)	Reference		0.800
		B. infantis Present		0.07 (-0.05, 0.18)	0.258	
	Any	*B. infantis* Absent		Reference		
		B. infantis Present		-0.01 (-0.17, 0.15)	0.937	
	None	*B. infantis* Absent		Reference		
		B. infantis Present		0.05 (-0.15, 0.25)	0.631	
1-Year	Overall	*B. infantis* Absent	Isopropanol	Reference		0.051
		B. infantis Present		0.12 (-0.04, 0.27)	0.145	
	Any	*B. infantis* Absent		Reference		
		B. infantis Present		0.18 (-0.02, 0.38)	0.084	
	None	*B. infantis* Absent		Reference		
		B. infantis Present		-0.21 (-0.49, 0.08)	0.159	

Timing of Feeding and *B. infantis* Measurement	Overall and Stratification by Human Milk Feeding Status	Presence/Absence of *B. infantis*	Metabolite	Adjusted Difference in Inverse Normal Transformed Metabolite Level β (95% CI)	*P*	*P for interaction* between Feeding and *B. infantis*
1-Year	Overall	*B. infantis* Absent	Lysine	Reference		0.474
		B. infantis Present		-0.40 (-0.55, -0.26)	0.000	
	Any	*B. infantis* Absent		Reference		
		B. infantis Present		-0.22 (-0.39, -0.04)	0.018	
	None	*B. infantis* Absent		Reference		
		B. infantis Present		-0.38 (-0.67, -0.10)	0.008	
1-Year	Overall	*B. infantis* Absent	N1-Acetylspermidine	Reference		0.697
		B. infantis Present		-0.23 (-0.39, -0.08)	0.003	
	Any	*B. infantis* Absent		Reference		
		B. infantis Present		-0.24 (-0.45, -0.04)	0.021	
	None	*B. infantis* Absent		Reference		
		B. infantis Present		-0.29 (-0.56, -0.01)	0.040	
1-Year	Overall	*B. infantis* Absent	Nicotinic-acid	Reference		0.820
		B. infantis Present		-0.11 (-0.26, 0.05)	0.171	
	Any	*B. infantis* Absent		Reference		
		B. infantis Present		-0.13 (-0.32, 0.07)	0.194	
	None	*B. infantis* Absent		Reference		
		B. infantis Present		-0.11 (-0.40, 0.17)	0.445	
1-Year	Overall	*B. infantis* Absent	PC ae C44:4	Reference		0.087
		B. infantis Present		0.14 (0.01, 0.27)	0.042	
	Any	*B. infantis* Absent		Reference		
		B. infantis Present		0.20 (0.03, 0.38)	0.024	
	None	*B. infantis* Absent		Reference		
		B. infantis Present		-0.09 (-0.34, 0.15)	0.447	

Estimates are based on mixed-effect linear regressions with metabolite levels transformed by rank-based inverse normal transformation as the outcome. SBP-related metabolites are identified by elastic net regression in the overall sample or within each stratum of feeding status (shown in eTable 5). Results with P for interaction<0.1 are further visualized in eFigure 3. Models adjusted for maternal age at delivery (continuous), pre-pregnancy body mass index (continuous), educational achievement (high school or below, college and university level, graduate degree), hypertension (chronic, during pregnancy, no), antibiotic use (prenatal, intrapartum, no), delivery mode (vaginal, C-section with labor, C-section without labor), child sex (boy, girl), birth weight (continuous), exact age in months (continuous) at fecal sample collection, time between sample collection and freezing (hours), with study site (Edmonton, Toronto, Vancouver, Winnipeg) as a random intercept.

eTable 9. Associations of human milk feeding status with indolelactic acid and short chain fatty acids at 3 months and 1 year, overall and by presence/absence of *B. infantis*.

Timing of Feeding and *B. infantis* Measurement	Overall and Stratification by Presence/Absence of *B. infantis*	Human Milk Feeding Status	Indolelactic Acid and Short Chain Fatty Acids	Adjusted Difference in Inverse Normal Transformed Metabolite Level β (95% CI)	*P*	*P for interaction* between Feeding and *B. infantis*
3 Months	Overall	None	Acetic acid	Reference		0.149
		Mixed		0.07 (-0.21, 0.35)	0.623	
		Exclusive		-0.12 (-0.43, 0.18)	0.423	
	B. infantis Present	None		Reference		
		Mixed		0.13 (-0.90, 1.15)	0.803	
		Exclusive		0.71 (-0.35, 1.78)	0.182	
	B. infantis Absent	None		Reference		
		Mixed		0.04 (-0.25, 0.34)	0.768	
		Exclusive		-0.27 (-0.60, 0.07)	0.115	
3 Months	Overall	None	Butyrate	Reference		0.828
		Mixed		-0.40 (-0.64, -0.15)	0.002	
		Exclusive		-0.81 (-1.09, -0.54)	0.000	
	B. infantis Present	None		Reference		
		Mixed		0.47 (-0.43, 1.37)	0.293	
		Exclusive		-0.36 (-1.30, 0.57)	0.435	
	B. infantis Absent	None		Reference		
		Mixed		-0.37 (-0.63, -0.11)	0.005	
		Exclusive		-0.77 (-1.06, -0.48)	0.000	
3 Months	Overall	None	Indolelactic acid	Reference		0.820
		Mixed		0.51 (0.26, 0.76)	0.000	
		Exclusive		0.51 (0.26, 0.76)	0.000	
	B. infantis Present	None		Reference		
		Mixed		0.03 (-0.84, 0.91)	0.940	
		Exclusive		0.30 (-0.58, 1.17)	0.497	
	B. infantis Absent	None		Reference		
		Mixed		0.48 (0.21, 0.74)	0.000	
		Exclusive		0.43 (0.16, 0.70)	0.002	
3 Months	Overall	None	Isobutyric acid	Reference		0.172
		Mixed		-0.30 (-0.55, -0.06)	0.016	
		Exclusive		-0.48 (-0.75, -0.21)	0.001	
	B. infantis Present	None		Reference		
		Mixed		-0.27 (-1.25, 0.70)	0.572	
		Exclusive		-0.13 (-1.15, 0.89)	0.799	
	B. infantis Absent	None		Reference		
		Mixed		-0.29 (-0.56, -0.03)	0.030	
		Exclusive		-0.55 (-0.84, -0.25)	0.000	
3 Months	Overall	None	Isovaleric acid	Reference		0.097
		Mixed		-0.67 (-0.91, -0.44)	0.000	
		Exclusive		-1.05 (-1.31, -0.78)	0.000	
	B. infantis Present	None		Reference		
		Mixed		-0.14 (-1.11, 0.83)	0.767	
		Exclusive		-0.38 (-1.40, 0.63)	0.449	
	B. infantis Absent	None		Reference		
		Mixed		-0.75 (-1.00, -0.50)	0.000	
		Exclusive		-1.18 (-1.46, -0.90)	0.000	
3 Months	Overall	None	Propionate	Reference		0.786
		Mixed		-0.21 (-0.45, 0.04)	0.104	
		Exclusive		-0.89 (-1.16, -0.62)	0.000	
	B. infantis Present	None		Reference		
		Mixed		-0.17 (-1.30, 0.97)	0.768	
		Exclusive		-0.94 (-2.13, 0.24)	0.113	

Timing of Feeding and *B. infantis* Measurement	Overall and Stratification by Presence/Absence of *B. infantis*	Human Milk Feeding Status	Indolelactic Acid and Short Chain Fatty Acids	Adjusted Difference in Inverse Normal Transformed Metabolite Level β (95% CI)	*P*	*P for interaction* between Feeding and *B. infantis*
	B. infantis Absent	None		Reference		
		Mixed		-0.18 (-0.44, 0.08)	0.181	
		Exclusive		-0.87 (-1.17, -0.58)	0.000	
3 Months	Overall	None	Valerate	Reference		0.639
		Mixed		-0.12 (-0.38, 0.14)	0.380	
		Exclusive		-0.67 (-0.96, -0.38)	0.000	
	B. infantis Present	None		Reference		
		Mixed		-0.02 (-0.96, 0.92)	0.966	
		Exclusive		-0.82 (-1.80, 0.17)	0.100	
	B. infantis Absent	None		Reference		
		Mixed		-0.16 (-0.45, 0.12)	0.264	
		Exclusive		-0.69 (-1.01, -0.37)	0.000	
1 Year	Overall	None	Acetic acid	Reference		0.773
		Any		0.04 (-0.09, 0.17)	0.538	
	B. infantis Present	None		Reference		
		Any		-0.05 (-0.44, 0.34)	0.805	
	B. infantis Absent	None		Reference		
		Any		0.03 (-0.11, 0.18)	0.638	
1 Year	Overall	None	Butyrate	Reference		0.566
		Any		0.03 (-0.09, 0.16)	0.605	
	B. infantis Present	None		Reference		
		Any		-0.01 (-0.41, 0.39)	0.964	
	B. infantis Absent	None		Reference		
		Any		0.05 (-0.09, 0.19)	0.470	
1 Year	Overall	None	Indolelactic acid	Reference		0.007
		Any		0.36 (0.24, 0.48)	0.000	
	B. infantis Present	None		Reference		
		Any		0.79 (0.45, 1.12)	0.000	
	B. infantis Absent	None		Reference		
		Any		0.19 (0.06, 0.32)	0.003	
1 Year	Overall	None	Isobutyric acid	Reference		0.557
		Any		0.05 (-0.07, 0.16)	0.422	
	B. infantis Present	None		Reference		
		Any		0.19 (-0.14, 0.52)	0.263	
	B. infantis Absent	None		Reference		
		Any		0.03 (-0.10, 0.16)	0.639	
1 Year	Overall	None	Isovaleric acid	Reference		0.886
		Any		-0.19 (-0.31, -0.06)	0.003	
	B. infantis Present	None		Reference		
		Any		-0.18 (-0.54, 0.18)	0.318	
	B. infantis Absent	None		Reference		
		Any		-0.17 (-0.31, -0.03)	0.016	
1 Year	Overall	None	Propionate	Reference		0.203
		Any		-0.03 (-0.16, 0.10)	0.607	
	B. infantis Present	None		Reference		
		Any		-0.25 (-0.63, 0.13)	0.200	
	B. infantis Absent	None		Reference		
		Any		0.01 (-0.13, 0.16)	0.855	
1 Year	Overall	None	Valerate	Reference		0.432
		Any		-0.08 (-0.21, 0.04)	0.195	
	B. infantis Present	None		Reference		
		Any		-0.26 (-0.60, 0.09)	0.142	
	B. infantis Absent	None		Reference		

Timing of Feeding and *B. infantis* Measurement	Overall and Stratification by Presence/Absence of *B. infantis*	Human Milk Feeding Status	Indolelactic Acid and Short Chain Fatty Acids	Adjusted Difference in Inverse Normal Transformed Metabolite Level β (95% CI)	*P*	*P for interaction* between Feeding and *B. infantis*
		Any		-0.06 (-0.20, 0.08)	0.429	

Estimates are based on mixed-effect linear regressions with metabolite levels transformed by rank-based inverse normal transformation as the outcome. Models adjusted for maternal age at delivery (continuous), pre-pregnancy body mass index (continuous), educational achievement (high school or below, college and university level, graduate degree), hypertension (chronic, during pregnancy, no), antibiotic use (prenatal, intrapartum, no), delivery mode (vaginal, C-section with labor, C-section without labor), child sex (boy, girl), birth weight (continuous), exact age in months (continuous) at fecal sample collection, time between sample collection and freezing (hours), with study site (Edmonton, Toronto, Vancouver, Winnipeg) as a random intercept. Abbreviation: CI, confidence interval.

eTable 10. Associations of presence/absence of *B. infantis* with indolelactic acid and short chain fatty acids at 3 months and 1 year, overall and by human milk feeding status.

Timing of Feeding and *B. infantis* Measurement	Overall and Stratification by Human Milk Feeding Status	Presence/Absence of *B. infantis*	Indolelactic Acid and Short Chain Fatty Acids	Adjusted Difference in Inverse Normal Transformed Metabolite Level β (95% CI)	*P*	*P for interaction* between Feeding and *B. infantis*
3 Months	Overall	*B. infantis* Absent	Acetic acid	Reference		0.149
		B. infantis Present		0.17 (-0.14, 0.49)	0.284	
	Exclusive	*B. infantis* Absent		Reference		
		B. infantis Present		0.55 (-0.01, 1.11)	0.053	
	Mixed	*B. infantis* Absent		Reference		
		B. infantis Present		0.01 (-0.51, 0.54)	0.956	
	None	*B. infantis* Absent		Reference		
		B. infantis Present		-0.15 (-0.89, 0.60)	0.697	
3 Months	Overall	*B. infantis* Absent	Butyrate	Reference		0.828
		B. infantis Present		-0.47 (-0.76, -0.18)	0.002	
	Exclusive	*B. infantis* Absent		Reference		
		B. infantis Present		-0.42 (-0.97, 0.12)	0.128	
	Mixed	*B. infantis* Absent		Reference		
		B. infantis Present		-0.28 (-0.71, 0.14)	0.188	
	None	*B. infantis* Absent		Reference		
		B. infantis Present		-0.51 (-1.23, 0.22)	0.167	
3 Months	Overall	*B. infantis* Absent	Indolelactic acid	Reference		0.820
		B. infantis Present		0.69 (0.42, 0.96)	0.000	
	Exclusive	*B. infantis* Absent		Reference		
		B. infantis Present		0.62 (0.19, 1.04)	0.005	
	Mixed	*B. infantis* Absent		Reference		
		B. infantis Present		0.57 (0.10, 1.03)	0.017	
	None	*B. infantis* Absent		Reference		
		B. infantis Present		0.66 (-0.03, 1.35)	0.060	
3 Months	Overall	*B. infantis* Absent	Isobutyric acid	Reference		0.172
		B. infantis Present		-0.02 (-0.31, 0.26)	0.868	
	Exclusive	*B. infantis* Absent		Reference		
		B. infantis Present		0.50 (0.09, 0.90)	0.016	
	Mixed	*B. infantis* Absent		Reference		
		B. infantis Present		-0.11 (-0.56, 0.34)	0.629	
	None	*B. infantis* Absent		Reference		
		B. infantis Present		-0.37 (-1.22, 0.47)	0.383	
3 Months	Overall	*B. infantis* Absent	Isovaleric acid	Reference		0.097
		B. infantis Present		-0.03 (-0.33, 0.26)	0.818	
	Exclusive	*B. infantis* Absent		Reference		
		B. infantis Present		0.43 (-0.08, 0.94)	0.099	
	Mixed	*B. infantis* Absent		Reference		
		B. infantis Present		0.16 (-0.31, 0.62)	0.505	
	None	*B. infantis* Absent		Reference		
		B. infantis Present		-0.56 (-1.20, 0.08)	0.088	
3 Months	Overall	*B. infantis* Absent	Propionate	Reference		0.786
		B. infantis Present		-0.35 (-0.65, -0.05)	0.023	
	Exclusive	*B. infantis* Absent		Reference		
		B. infantis Present		-0.44 (-0.94, 0.05)	0.079	
	Mixed	*B. infantis* Absent		Reference		
		B. infantis Present		-0.15 (-0.62, 0.32)	0.538	
	None	*B. infantis* Absent		Reference		
		B. infantis Present		-0.45 (-1.09, 0.19)	0.169	
3 Months	Overall	*B. infantis* Absent	Valerate	Reference		0.639
		B. infantis Present		-0.05 (-0.36, 0.26)	0.738	
	Exclusive	*B. infantis* Absent		Reference		

Timing of Feeding and *B. infantis* Measurement	Overall and Stratification by Human Milk Feeding Status	Presence/Absence of *B. infantis*	Indolelactic Acid and Short Chain Fatty Acids	Adjusted Difference in Inverse Normal Transformed Metabolite Level β (95% CI)	*P*	*P for interaction* between Feeding and *B. infantis*
		B. infantis Present		0.00 (-0.50, 0.50)	0.999	
	Mixed	*B. infantis* Absent		Reference		
		B. infantis Present		0.16 (-0.35, 0.68)	0.532	
	None	*B. infantis* Absent		Reference		
		B. infantis Present		-0.19 (-0.95, 0.57)	0.617	
1 Year	Overall	*B. infantis* Absent	Acetic acid	Reference		0.149
		B. infantis Present		0.14 (-0.02, 0.30)	0.081	
	Any	B. infantis Absent		Reference		
		B. infantis Present		0.15 (-0.06, 0.37)	0.154	
	None	B. infantis Absent		Reference		
		B. infantis Present		0.15 (-0.14, 0.45)	0.311	
1 Year	Overall	*B. infantis* Absent	Butyrate	Reference		0.828
		B. infantis Present		0.02 (-0.14, 0.18)	0.845	
	Any	B. infantis Absent		Reference		
		B. infantis Present		0.00 (-0.21, 0.22)	0.994	
	None	B. infantis Absent		Reference		
		B. infantis Present		0.04 (-0.24, 0.32)	0.775	
1 Year	Overall	*B. infantis* Absent	Indolelactic acid	Reference		0.820
		B. infantis Present		0.51 (0.36, 0.66)	0.000	
	Any	B. infantis Absent		Reference		
		B. infantis Present		0.57 (0.39, 0.75)	0.000	
	None	B. infantis Absent		Reference		
		B. infantis Present		0.16 (-0.12, 0.45)	0.267	
1 Year	Overall	*B. infantis* Absent	Isobutyric acid	Reference		0.172
		B. infantis Present		0.04 (-0.11, 0.18)	0.591	
	Any	B. infantis Absent		Reference		
		B. infantis Present		0.05 (-0.13, 0.23)	0.566	
	None	B. infantis Absent		Reference		
		B. infantis Present		-0.07 (-0.35, 0.21)	0.615	
1 Year	Overall	*B. infantis* Absent	Isovaleric acid	Reference		0.097
		B. infantis Present		-0.13 (-0.28, 0.03)	0.108	
	Any	B. infantis Absent		Reference		
		B. infantis Present		-0.08 (-0.28, 0.13)	0.454	
	None	B. infantis Absent		Reference		
		B. infantis Present		-0.08 (-0.37, 0.21)	0.586	
1 Year	Overall	*B. infantis* Absent	Propionate	Reference		0.786
		B. infantis Present		-0.02 (-0.18, 0.14)	0.831	
	Any	B. infantis Absent		Reference		
		B. infantis Present		-0.10 (-0.30, 0.10)	0.336	
	None	B. infantis Absent		Reference		
		B. infantis Present		0.09 (-0.22, 0.39)	0.564	
1 Year	Overall	*B. infantis* Absent	Valerate	Reference		0.639
		B. infantis Present		-0.05 (-0.21, 0.10)	0.506	
	Any	B. infantis Absent		Reference		
		B. infantis Present		-0.05 (-0.24, 0.13)	0.576	
	None	B. infantis Absent		Reference		
		B. infantis Present		0.05 (-0.26, 0.35)	0.770	

Estimates are based on mixed-effect linear regressions with metabolite levels transformed by rank-based inverse normal transformation as the outcome. Models adjusted for maternal age at delivery (continuous), pre-pregnancy body mass index (continuous), educational achievement (high school or below, college and university level, graduate degree), hypertension (chronic, during pregnancy, no), antibiotic use (prenatal, intrapartum, no), delivery mode (vaginal, C-section with labor, C-section without labor), child sex (boy, girl), birth weight (continuous), exact age in months (continuous) at fecal sample collection, time between sample collection and freezing (hours), with study site (Edmonton, Toronto, Vancouver, Winnipeg) as a random intercept. Abbreviations: SBP, systolic blood pressure; CI, confidence interval.

eTable 11. Fecal metabolites in infancy that are associated with SBP percentile in childhood, overall and within each stratum of human milk feeding status, identified by assay-specific elastic net regression.

Timing of Metabolites and Feeding Measurement	Overall and Stratification by Human Feeding Status	Metabolite	Assay	Outcome	Elastic Net Coefficients
3 Months	Overall	Nicotinic-acid	NMR	SBP at 3 Years	1.29
	Exclusive	DG(14:0_18:1)	LC-MS/MS		1.39
		PC aa C28:1	LC-MS/MS		-1.69
		CE(22:1)	LC-MS/MS		-0.96
		PC38:0AA	LC-MS/MS		-0.76
		LYSOC18:2	LC-MS/MS		-0.29
	Mixed	Methylmalonic acid	LC-MS/MS		1.20
		Tyramine	LC-MS/MS		1.16
		PC aa C42:2	LC-MS/MS		1.02
		Hex2Cer(d18:1/24:1)	LC-MS/MS		0.98
		Ethanolamine	LC-MS/MS		0.76
		LYSOC26:1	LC-MS/MS		0.73
		PC aa C36:4	LC-MS/MS		0.65
		Cer(d18:0/24:1)	LC-MS/MS		0.28
		PC aa C42:6	LC-MS/MS		-0.88
	None	Formate	NMR		2.50
		3-Phenylpropionate	NMR		-2.47
		Ethanol	NMR		-1.86
3 Months	Overall	Succinic acid	LC-MS/MS	SBP at 5 Years	1.11
		CE(20:1)	LC-MS/MS		0.79
		C16:1	LC-MS/MS		0.76
		CE(20:0)	LC-MS/MS		0.60
		N-Acetyl-Asp	LC-MS/MS		0.55
		Creatinine	LC-MS/MS		0.44
		C0	LC-MS/MS		0.36
		4-Hydroxyphenylacetic acid	LC-MS/MS		0.34
		Uridine	LC-MS/MS		0.34
		CE(18:1)	LC-MS/MS		-1.22
		Histamine	LC-MS/MS		-0.65
		CE(18:0)	LC-MS/MS		-0.64
		Cer(d18:1/24:0)	LC-MS/MS		-0.46
	Exclusive	C16:1	LC-MS/MS		1.40
		Methanol	NMR		-0.68
		Isocitric acid	LC-MS/MS		-0.51
	Mixed	PC ae C34:2	LC-MS/MS		0.79
	None	Arabinose	NMR		2.09
		C0	LC-MS/MS		1.95
		C4OH	LC-MS/MS		1.95
		Guanidinopropionic acid	LC-MS/MS		1.75
		4-Hydroxyphenylacetic acid	LC-MS/MS		1.66
		CE(20:0)	LC-MS/MS		1.51
		Oxalacetic acid	LC-MS/MS		1.28
		Isocitric acid	LC-MS/MS		1.23
		CE(15:1)	LC-MS/MS		1.21
		DG(16:0_16:0)	LC-MS/MS		1.10
		N-Acetyl-His	LC-MS/MS		1.01
		Tartaric acid	LC-MS/MS		1.01
		N2-Acetyl-Orn	LC-MS/MS		0.97
		Cadaverine	NMR		0.95
		3-Hydroxyisobutyric acid	LC-MS/MS		0.95
		DG(14:0_18:1)	LC-MS/MS		0.88

Timing of Metabolites and Feeding Measurement	Overall and Stratification by Human Feeding Status	Metabolite	Assay	Outcome	Elastic Net Coefficients
		N-Acetyl-Tyr	LC-MS/MS		0.87
		N-Acetyl-Met	LC-MS/MS		0.37
		2-Hydroxy-3-methylvaleric acid	LC-MS/MS		0.23
		PC aa C38:5	LC-MS/MS		-1.73
		PC ae C42:5	LC-MS/MS		-1.66
		PC ae C36:2	LC-MS/MS		-1.55
		Tryptamine	LC-MS/MS		-1.30
		22:2SMOH	LC-MS/MS		-1.14
		16:1SMOH	LC-MS/MS		-0.84
		SM C26:0	LC-MS/MS		-0.75
		Histamine	LC-MS/MS		-0.74
		Methylamine	LC-MS/MS		-0.32
1 Year	Overall	PC ae C44:4	LC-MS/MS	SBP at 5 Years	1.02
		CE(18:1)	LC-MS/MS		0.78
		C2	LC-MS/MS		0.62
		DOPA	LC-MS/MS		0.58
		Arabinose	NMR		0.50
		PC36:6AA	LC-MS/MS		0.28
		Histidine	LC-MS/MS		0.23
		CE(18:3)	LC-MS/MS		-0.95
		Nicotinic-acid	NMR		-0.83
		Isopropanol	NMR		-0.58
		Lysine	LC-MS/MS		-0.51
		Diacetylspermine	LC-MS/MS		-0.42
		Glutaric acid	LC-MS/MS		-0.38
		Argininic acid	LC-MS/MS		-0.37
		N-Acetyl-Leu	LC-MS/MS		-0.37
		C3:1	LC-MS/MS		-0.31
		N-Methyl-Asp	LC-MS/MS		-0.30
		C7DC	LC-MS/MS		-0.23
		DG(17:0_18:1)	LC-MS/MS		-0.23
		HexCer(d18:1/18:0)	LC-MS/MS		-0.21
		N1-Acetylspermidine	LC-MS/MS		-0.19
		N2-Acetyl-Orn	LC-MS/MS		-0.18
	None	Argininic acid	LC-MS/MS		-1.04
		Nicotinic-acid	NMR		-0.71
		Glutamic acid	LC-MS/MS		-0.46

Significant metabolites are identified by assay-specific (i.e., NMR, LC-MS/MS) elastic net regression conducted overall and within each category of human milk feeding status assessed at the time of metabolite measurement. Models accounted for maternal age at delivery (continuous), pre-pregnancy body mass index (continuous), educational achievement (high school or below, college and university level, graduate degree), hypertension (chronic, during pregnancy, no), antibiotic use (prenatal, intrapartum, no), delivery mode (vaginal, C-section with labor, C-section without labor), child sex (boy, girl), birth weight (continuous), exact age in months (continuous) at fecal sample collection, time between sample collection and freezing (hours), and study site (Edmonton, Toronto, Vancouver, Winnipeg). Abbreviation: SBP, systolic blood pressure.

eTable 12. Differentially abundant fecal microbes at 3 month and 1 year in association with SBP percentiles at 3 and 5 years, identified by MaAsLin2.

Microbe	Timing of Microbiome Measurement	Outcome	Effect Size	Standard Error	*P*	*FDR-P*
Bacteroides uniformis SGB1836	3 Month	SBP at 3 years	0.12	0.13	0.352	0.691
		SBP at 5 years	0.25	0.12	0.040	0.225
	1 Year	SBP at 3 years	0.18	0.17	0.269	0.729
		SBP at 5 years	-0.08	0.17	0.625	0.907
Clostridiaceae bacterium SGB4269	1 Year	SBP at 3 years	-0.32	0.14	0.022	0.230
		SBP at 5 years	-0.05	0.14	0.720	0.931
Clostridium innocuum SGB4037	3 Month	SBP at 3 years	-0.03	0.11	0.784	0.935
		SBP at 5 years	-0.26	0.11	0.021	0.146
	1 Year	SBP at 3 years	0.05	0.17	0.772	0.944
		SBP at 5 years	-0.10	0.17	0.550	0.872
Clostridium sp C5 48 SGB4752	1 Year	SBP at 3 years	0.38	0.16	0.018	0.200
		SBP at 5 years	0.15	0.16	0.346	0.754
Eggerthella lenta SGB14809	3 Month	SBP at 3 years	-0.36	0.16	0.025	0.166
		SBP at 5 years	-0.35	0.16	0.028	0.177
	1 Year	SBP at 3 years	-0.13	0.12	0.254	0.718
		SBP at 5 years	-0.11	0.12	0.372	0.767
Staphylococcus aureus SGB7852	3 Month	SBP at 3 years	-0.21	0.08	0.006	0.055
		SBP at 5 years	0.02	0.08	0.786	0.922
Streptococcus parasanguinis SGB8071	3 Month	SBP at 3 years	-0.39	0.12	0.001	0.011
		SBP at 5 years	-0.05	0.12	0.684	0.880
	1 Year	SBP at 3 years	-0.03	0.11	0.784	0.946
		SBP at 5 years	0.03	0.10	0.754	0.941
Tyzzerella nexilis SGB4588	1 Year	SBP at 3 years	0.35	0.15	0.022	0.229
		SBP at 5 years	0.09	0.15	0.570	0.878
Veillonella dispar SGB6952	3 Month	SBP at 3 years	-0.31	0.13	0.015	0.112
		SBP at 5 years	-0.07	0.13	0.607	0.857
	1 Year	SBP at 3 years	0.20	0.12	0.109	0.527
		SBP at 5 years	0.29	0.12	0.020	0.193
Veillonella parvula SGB6939	3 Month	SBP at 3 years	-0.31	0.15	0.045	0.250
		SBP at 5 years	-0.47	0.15	0.002	0.022
	1 Year	SBP at 3 years	0.04	0.15	0.777	0.946
		SBP at 5 years	0.23	0.14	0.121	0.553

Models adjusted for maternal age at delivery (continuous), pre-pregnancy body mass index (continuous), educational achievement (high school or below, college and university level, graduate degree), hypertension (chronic, during pregnancy, no), antibiotic use (prenatal, intrapartum, no), delivery mode (vaginal, C-section with labor, C-section without labor), child sex (boy, girl), birth weight (continuous), and exact age in months (continuous) at fecal sample collection, with study site (Edmonton, Toronto, Vancouver, Winnipeg) as a random intercept. Abbreviations: SBP, systolic blood pressure; FDR, false discovery rate. Abbreviations: SBP, systolic blood pressure; FDR, false discovery rate.

eTable 13. Associations between relative abundances of differentially abundant microbes in infancy identified by MaAsLin2 and SBP percentile in childhood, overall and stratified by human milk feeding status.

Timing of Microbiome Measurement	Microbe	Overall and Stratification by Human Milk Feeding Status	Adjusted Difference in SBP Percentile β (95% CI)	*P*	*P for interaction* between Feeding and Microbes
3 Months	*Bacteroides uniformis SGB1836*	Overall	0.26 (-0.02, 0.55)	0.068	0.517
		Exclusive	0.14 (-0.24, 0.51)	0.471	
		Mixed	0.23 (-0.33, 0.79)	0.426	
		None	0.74 (-0.05, 1.53)	0.067	
3 Months	*Clostridium innocuum SGB4037*	Overall	-0.19 (-0.45, 0.07)	0.159	0.249
		Exclusive	0.08 (-0.41, 0.57)	0.745	
		Mixed	-0.40 (-0.85, 0.05)	0.084	
		None	-0.50 (-1.03, 0.03)	0.062	
3 Months	*Eggerthella lenta SGB14809*	Overall	-0.26 (-0.46, -0.05)	0.016	0.007
		Exclusive	-0.01 (-0.29, 0.27)	0.956	
		Mixed	-0.54 (-0.95, -0.12)	0.011	
		None	-0.91 (-1.48, -0.34)	0.002	
3 Months	*Staphylococcus aureus SGB7852*	Overall	-0.27 (-0.65, 0.11)	0.160	0.753
		Exclusive	-0.37 (-0.82, 0.07)	0.100	
		Mixed	0.02 (-0.84, 0.89)	0.959	
		None	-0.67 (-2.59, 1.26)	0.496	
3 Months	*Streptococcus parasanguinis SGB8071*	Overall	-0.29 (-0.55, -0.02)	0.035	0.656
		Exclusive	-0.24 (-0.59, 0.11)	0.184	
		Mixed	-0.35 (-0.90, 0.20)	0.216	
		None	-0.69 (-1.40, 0.01)	0.053	
3 Months	*Veillonella dispar SGB6952*	Overall	-0.23 (-0.52, 0.06)	0.120	0.539
		Exclusive	-0.28 (-0.65, 0.08)	0.127	
		Mixed	-0.10 (-0.67, 0.46)	0.716	
		None	-0.75 (-1.69, 0.18)	0.114	
3 Months	*Veillonella parvula SGB6939*	Overall	-0.27 (-0.48, -0.06)	0.012	0.431
		Exclusive	-0.20 (-0.46, 0.06)	0.139	
		Mixed	-0.64 (-1.11, -0.16)	0.009	
		None	-0.34 (-0.95, 0.28)	0.282	
1 Year	*Clostridiaceae bacterium SGB4269*	Overall	-0.18 (-0.40, 0.05)	0.122	0.046
		Any	0.09 (-0.24, 0.42)	0.595	
		None	-0.37 (-0.68, -0.07)	0.018	
1 Year	*Clostridium sp C5 48 SGB4752*	Overall	0.22 (-0.00, 0.44)	0.053	0.929
		Any	0.24 (-0.09, 0.58)	0.149	
		None	0.23 (-0.08, 0.54)	0.148	
1 Year	*Tyzzerella nexilis SGB4588*	Overall	0.18 (-0.01, 0.37)	0.059	0.143
		Any	0.43 (0.12, 0.74)	0.007	
		None	0.09 (-0.17, 0.34)	0.515	
1 Year	*Veillonella dispar SGB6952*	Overall	0.36 (0.09, 0.63)	0.009	0.026
		Any	0.11 (-0.26, 0.47)	0.563	
		None	0.76 (0.35, 1.18)	0.000	

Estimates are from mixed-effect linear regression with centered log ratio transformed relative abundances of each microbe as the exposure, and systolic blood pressure (SBP) percentile measured at 3 and 5 years modeled as a repeated outcome. Microbes shown here are chosen because of significant associations identified by MaAsLin2 when SBP percentiles at 3 and 5 years are modeled separately (results shown in eTable 11). Models adjusted for maternal age at delivery (continuous), pre-pregnancy body mass index (continuous), educational achievement (high school or below, college and university level, graduate degree), hypertension (chronic, during pregnancy, no), antibiotic use (prenatal, intrapartum, no), delivery mode (vaginal, C-section with labor, C-section without labor), child sex (boy, girl), birth weight (continuous), and exact age in months (continuous) at fecal sample collection, with study site (Edmonton, Toronto, Vancouver, Winnipeg) as a random intercept. Abbreviations: SBP, systolic blood pressure; CI, confidence interval.

eTable 14. Correlation matrix between SBP-related microbes and SBP-related metabolites at 3 months and 1 year.

3 Months																				
	22:1SMOH	C14	C16OH	C18	CE(18:0)	CE(18:1)	CE(20:0)	Cer(d18:1/24:0)	Creatinine	DG(16:1_20:0)	DOPA	Histamine	LYSOC28:0	N-Acetyl-Asp	N-Acetyl-Met	N-Acetyl-Pro	PC ae C30:1	PC ae C40:4	Succinic acid	TG(16:0_38:3)
Bacteroides uniformis SGB1836	-0.05	-0.09	-0.07	0.00	0.02	-0.00	0.01	-0.02	-0.04	-0.01	-0.00	0.03	0.01	0.10	0.07	0.01	-0.07	-0.06	**0.15**	-0.04
Clostridium innocuum SGB4037	-0.08	**-0.15**	-0.05	**-0.17**	0.05	0.01	-0.03	0.04	-0.08	-0.02	0.09	-0.10	-0.00	**-0.13**	0.08	-0.02	0.04	-0.04	-0.08	0.00
Eggerthella lenta SGB14809	-0.08	**-0.37**	**-0.27**	**-0.21**	-0.01	-0.03	0.01	0.00	**-0.35**	-0.04	0.05	-0.02	-0.02	-0.04	0.08	0.08	-0.02	-0.10	-0.01	-0.03
Staphylococcus aureus SGB7852	0.12	0.10	0.01	0.09	0.06	**0.15**	0.11	0.11	0.06	0.02	-0.02	0.02	0.01	0.02	0.00	-0.05	0.04	0.03	0.07	0.11
Streptococcus parasanguinis SGB8071	-0.03	0.07	0.05	-0.04	0.05	-0.06	-0.06	0.02	-0.01	**0.13**	0.13	-0.05	0.02	-0.06	0.01	0.02	-0.07	0.02	0.02	0.06

Veillonella dispar SGB6952	**0.13**	0.09	-0.04	0.02	0.01	0.02	0.02	-0.04	**0.14**	0.04	**0.15**	**0.13**	0.04	-0.03	-0.03	-0.03	0.00	0.00	-0.01	0.05
Veillonella parvula SGB6939	0.06	-0.01	-0.01	-0.05	0.04	0.08	-0.01	-0.01	0.08	-0.02	0.08	0.10	0.04	**-0.19**	0.06	-0.00	0.03	0.05	**-0.19**	0.08
1 Year																				
	C2	C3:1	CE(18:1)	CE(18:3)	Diacetylspermine	DOPA	Isopropanol	Lysine	N1-Acetylspermidine	Nicotinic-acid	PC ae C44:4									
Clostridiaceae bacterium SGB4269	-0.06	0.02	-0.01	-0.02	**0.08**	0.10	-0.05	0.07	**0.21**	0.06	-0.02									
Clostridium sp C5 48 SGB4752	0.01	-0.02	0.01	0.03	-0.04	0.02	0.00	0.06	-0.01	0.03	-0.03									
Tyzzerella nexilis SGB4588	-0.01	**0.08**	-0.00	0.02	**-0.11**	0.02	0.02	**0.20**	-0.02	-0.01	-0.04									
Veillonella dispar SGB6952	0.07	**-0.10**	0.06	0.05	0.05	**0.14**	-0.04	**-0.11**	-0.05	**-0.10**	0.05									

Spearman correlation coefficients between microbes and metabolites associated with systolic blood pressure (SBP). Significant microbes were identified using MaAsLin2 (eTable 11), and significant metabolites were identified using elastic net regression (eTable 5). A visual representation of these correlations is provided in eFigure 4. Bolded values indicate statistically significant correlations after controlling for multiple comparisons using the Benjamini–Hochberg method (*FDR-P*<0.05).

eTable 15. Association between Shannon index in infancy and SBP percentile in childhood, overall and stratified by human milk feeding status.

Timing of Microbiome and Feeding Measurement	Overall and Stratification by Human Feeding Status	N	Adjusted Difference in SBP Percentile β (95% CI)	*P for interaction* between Shannon Index and Feeding
3 Months	Overall	1306	-0.85 (-2.62, 0.92)	0.804
	Exclusive	786	-0.84 (-3.14, 1.45)	
	Mixed	348	-2.57 (-6.16, 1.02)	
	None	172	-0.36 (-5.41, 4.69)	
1 Year	Overall	1283	-1.06 (-2.68, 0.56)	0.052
	Any	610	0.56 (-1.72, 2.84)	
	None	673	-2.67 (-5.04, -0.29)	

Estimates are from mixed-effect linear regression with SBP percentile measured at 3 and 5 years modeled as a repeated outcome. Models adjusted for maternal age at delivery (continuous), pre-pregnancy BMI (continuous), educational achievement (high school or below, college and university level, graduate degree), hypertension (chronic, during pregnancy, no), antibiotic use (prenatal, intrapartum, no), delivery mode (vaginal, C-section with labor, C-section without labor), child sex (when not tested as a modifier; boy, girl), birth weight (continuous), and exact age in months (continuous) at fecal sample collection, and breastfeeding status (when not tested as a modifier; exclusive, mixed, no at 3 months; yes, no at 1 year), with study site (Edmonton, Toronto, Vancouver, Winnipeg) as a random intercept. Abbreviations: SBP, systolic blood pressure; CI, confidence interval; BMI, body mass index.
