# Supplementary material for: Human Milk and Infant Gut Microbiome in Association With Infant Fecal Metabolome and Child Blood Pressure
Source: JAMA Netw Open. 2026 Feb 13;9(2):e2559385. doi: 10.1001/jamanetworkopen.2025.59385 (PMC12905658; doi:10.1001/jamanetworkopen.2025.59385)
Supplement: Supplement 2. — Data Sharing Statement [file jamanetwopen-e2559385-s002.pdf]

Data Sharing Statement

Liu. Human Milk and Infant Gut Microbiome in Association With Infant Fecal Metabolome and Child Blood Pressure. *JAMA Netw Open*. Published February 13, 2026.
doi:10.1001/jamanetworkopen.2025.59385

Data
Data available: Yes
Data types: Deidentified participant data
How to access data: noel.mueller@cuanschutz.edu
When available: With publication

Supporting Documents
Document types: None

Additional Information
Who can access the data: researchers whose proposed use of the data has been approved
Types of analyses: for a specified purpose
Mechanisms of data availability: without investigator support
